# A formative study exploring perceptions of physical activity and physical activity monitoring among children and young people with cystic fibrosis and health care professionals

**DOI:** 10.1186/s12887-018-1301-x

**Published:** 2018-10-23

**Authors:** James Shelley, Stuart J Fairclough, Zoe R Knowles, Kevin W Southern, Pamela McCormack, Ellen A Dawson, Lee E F Graves, Claire Hanlon

**Affiliations:** 1 Physical Activity Exchange, Research Institute for Sport and Exercise Sciences, Liverpool John Moores University, 62 Great Crosshall Street, Liverpool, L3 2AT England; 2 Department of Women’s and Children’s Health, University of Liverpool, Institute in the Park, Alder Hey Children’s Hospital, Eaton Road, L12 2AP Liverpool, England; 3 Respiratory Department, Alder Hey NHS Foundation Trust Children’s Hospital, Eaton Road, Liverpool, L12 2AP England; 4 Edge Hill University, St Helens Road, Ormskirk, Lancashire L39 4QP England

**Keywords:** Youth physical activity promotion, Fitbit, GENEActiv, ActiGraph, Qualitative

## Abstract

**Background:**

Physical activity (PA) is associated with reduced hospitalisations and maintenance of lung function in patients with Cystic Fibrosis (CF). PA is therefore recommended as part of standard care. Despite this, there is no consensus for monitoring of PA and little is known about perceptions of PA monitoring among children and young people with CF. Therefore, the research aimed to explore patients’ perceptions of PA and the acceptability of using PA monitoring devices with children and young people with CF.

**Methods:**

An action research approach was utilised, whereby findings from earlier research phases informed subsequent phases. Four phases were utilised, including patient interviews, PA monitoring, follow-up patient interviews and health care professional (HCP) interviews. Subsequently, an expert panel discussed the study to develop recommendations for practice and future research.

**Results:**

Findings suggest that experiences of PA in children and young people with CF are largely comparable to their non-CF peers, with individuals engaging in a variety of activities. CF was not perceived as a barrier per se, although participants acknowledged that they could be limited by their symptoms. Maintenance of health emerged as a key facilitator, in some cases PA offered patients the opportunity to *‘normalise’* their condition.

Participants reported enjoying wearing the monitoring devices and had good compliance. Wrist-worn devices and devices providing feedback were preferred. HCPs recognised the potential benefits of the devices in clinical practice.

Recommendations based on these findings are that interventions to promote PA in children and young people with CF should be individualised and involve families to promote PA as part of an active lifestyle. Patients should receive support alongside the PA data obtained from monitoring devices.

**Conclusions:**

PA monitoring devices appear to be an acceptable method for objective assessment of PA among children and young people with CF and their clinicians. Wrist-worn devices, which are unobtrusive and can display feedback, were perceived as most acceptable. By understanding the factors impacting PA, CF health professionals will be better placed to support patients and improve health outcomes.

**Electronic supplementary material:**

The online version of this article (10.1186/s12887-018-1301-x) contains supplementary material, which is available to authorized users.

## Background

Cystic Fibrosis (CF) affects approximately 11,000 individuals in the United Kingdom (UK), with median predicted survival reported as 45 years of age [1]. CF is an autosomal recessive disorder caused by mutation of the CF Transmembrane Conductance Regulator (*CFTR*) gene. The CFTR protein has an important role in co-ordinating transepithelial salt transport, which impacts on a number of important physiological functions [2]. Most importantly, the salt transport defect impairs mucociliary airway clearance by disrupting the airway surface liquid and predisposing the airway to a build-up of excess and viscous mucus. Subsequent chronic airway infection and inflammation lead to airway damage and eventual respiratory failure as the primary cause of early death [1]. In addition, the CF defect impacts on other epithelial surfaces, such as the sweat gland, pancreas and liver [3].

CF is also characterised by reduced exercise capacity [4] and, although the exact mechanisms are not yet fully understood, physical inactivity, pulmonary, cardiac, and peripheral skeletal muscle function all contribute [5]. Critically, higher aerobic fitness is associated with reduced mortality in patients with CF and therefore provides useful prognostic information [4]. Furthermore, physical activity (PA) is related to aerobic fitness, independent of sex, lung function, body size and muscle power [6], with higher PA associated with a slower decline in lung function [7] and fewer hospitalisations [8]. There is good evidence that PA has a positive impact on bone mineral density [9], glycaemic control [10] and mucociliary clearance [11], all of which contribute to wellbeing for a person with CF.

PA promotion and exercise prescription are currently recommended as part of standard CF care, alongside chest physiotherapy [12]. Despite the documented benefits and clinical recommendations there is some evidence to suggest that children with CF engage in less strenuous PA than age-matched controls [13]. The reduction in PA has been attributed to a number of perceived barriers including progressive lung function decline, symptoms of breathlessness, coughing and fatigue [14] as well as the burdensome treatment regimen associated with CF [15]. High treatment burden may influence the acceptability of additional measures such as PA monitoring. Additionally, patients with CF are experts in their condition and are typically very engaged in their medical care and self-care. They would need to value PA monitoring to make PA assessment feasible.

In order to provide guidance on PA, clinicians require knowledge of population specific barriers and facilitators for PA as perceived by children and young people with CF. Despite PA assessment and advice being perceived as important by clinicians, PA assessment is not common or consistent in CF centres [16]. Moreover, there is no consensus for monitoring or reporting of PA in CF [16]. There is little known about perceptions and acceptability of PA monitoring among children and young people with CF. Previous research has highlighted the need to better understand self-efficacy for PA and suggests that self-monitoring is central to all PA behaviours [17]. Though this research does not specifically refer to self-monitoring via monitoring devices it is possible that PA monitoring devices may feed into this self-monitoring process. Accordingly, exploring clinicians’ knowledge and perceptions of PA monitoring as well as those of the patients is needed to move towards the utilisation of PA monitoring devices as part of routine clinical care.

## Methods

### Ethical considerations

Ethical approval was sought and granted by South West Cornwall and Plymouth National Health Service (NHS) Research Ethics Committee and Liverpool John Moores University Ethics Committee prior to data collection. NHS Caldicott principles were strictly adhered to throughout, all data were anonymised and all personal details kept confidential. Parents/carers written consent and participants’ written assent were obtained. Parents/carers were also invited to be present during their child’s interviews.

The Medicines for Children Research Network Clinical Trials Network (MCRN) consultative group situated at Alder Hey Children’s NHS Foundation Trust, were consulted to appraise the ‘appropriateness’ of the language used in the study participant information sheets, consent forms and interview schedule. To ensure face validity, the interview schedule was reviewed during development, prior to phase one commencement.

### Aims

The overarching aim of the research was to explore the use of PA monitoring devices with children and young people with CF. As part of this, our objectives were to: (1) explore barriers, facilitators and perceptions of PA among children and young people with CF; (2) explore the acceptability of a range of PA monitoring devices; (3) explore clinicians’ existing knowledge and perceptions of PA monitoring, and; (4) explore the clinical application of PA monitoring as part of routine clinical care as well as identifying any disease specific limitations to PA monitoring.

### Design

An action research approach was utilised to achieve the study objectives whereby an iterative approach was used with findings from earlier research phases informing subsequent phases, of which there were four in total (see Additional file 1). The first included patient interviews to explore perceptions of PA. The second phase included the allocation of PA monitoring devices. Phase three had two components which ran simultaneously. The first included follow-up patient interviews informed by phases one and two. The second included health care professional interviews. Phase four included an adapted consensus approach, involving an expert panel to discuss the study findings, to develop recommendations and to inform future research directions. A researcher trained in qualitative research methods (CH) conducted all interviews and subsequent analysis.

### Participants

Participants were recruited from the CF clinic at Alder Hey Children’s Hospital. Potential participants were identified by members of the usual care team from the clinic database. Inclusion criteria included participants aged between 8 and 16 years, with a confirmed diagnosis of CF. Initially, potential participants were approached during their routine clinical appointments by a member of their CF care team, who briefly explained the purpose of the study. Parents/carers of children and young people who expressed an informal interest were later formally invited to participate in the study by a researcher via telephone. From 13 potential participants initially identified, 9 formally agreed to participate (5 female; mean age 12 ± 3 years). The remaining 4 either verbalised they were no longer willing to participate or the researcher was unable contact their parents/carers.

### Procedure

#### Phase 1

An open-ended, semi-structured interview protocol was devised using the principal enabling, reinforcing and predisposing factors of the Youth Physical Activity Promotion Model (YPAPM) [18]. The YPAPM is consistent with the socio-ecological model of health promotion and describes the hypothesised influences of diverse correlates on children’s and young people’s PA participation. Use of the YPAPM facilitated the development of a theory-driven interview schedule that would elicit beliefs and attitudes towards PA and reveal psychological enablers and barriers of PA as perceived by each individual, whilst allowing the researcher to explore individual nuances relating to the experience of PA in children and young people with CF.

Semi-structured interviews were conducted between children and young people with CF and the researcher (CH). It was felt that parent/carer presence would be reassuring for the participants, creating a positive and comfortable interview environment. In the interest of inclusivity some interview questions were directed specifically towards the parent/carer, however these questions were separated before analysis. Parents/carers were also asked, where appropriate, to prompt their child only to expand upon examples given when explaining their experiences of PA and not to respond directly for them. Interviews were arranged to take place at a time and place most convenient to the participants parents/carers, the majority were in the participants own home, with one at Alder Hey Children’s NHS Foundation Trust Hospital.

##### Data analysis

Participants were assigned a participant number (P1–P9) to protect participant confidentiality. Data analysis utilised a broad model of interpretative phenomenology as described by Fereday et al. [19]. Identification of emerging themes at the manifest level were discussed and coded between two researchers (CH & ZK) following each interview. Resultant themes were further discussed with reference to the interview data until final themes were determined and clustered around the YPAPM. As a result, no new themes were identified following interview with P9. Transcripts were deductively and inductively coded, illustrative quotes were extracted and clustered around emergent themes anchored according to the main factors of the YPAPM.

#### Phase 2

Phase 2 commenced midway through phase 1 and the two phases ran concurrently until the final participant interview was completed.

Each of the 9 participants were allocated PA monitoring devices to assess their acceptability in children and young people with CF. Allocation ensured that each participant received a research grade device alongside a consumer level device. Participants wore either a GENEActiv (ActivInsights Ltd., Cambs, UK) or ActiGraph GT3X+ (ActiGraph, Pensacola, FL) triaxial accelerometer on the non-dominant wrist or left hip, respectively. Both devices have demonstrated acceptable validity and reliability for use with children and young people [20]. In addition to the accelerometers participants were allocated either a Yamax Digiwalker pedometer (Yamax UK and Europe, Tasley UK) or one of two consumer level PA trackers; ‘Fitbit Flex’ or ‘Moves’ smartphone application (Table 2). Unfortunately, only one participant was able to trial the ‘Moves’ application, as it required participants to have a compatible smartphone. Participants were asked to wear the devices for seven consecutive days and were provided with instructions for how to wear the devices as well as an information sheet. Participants were asked to wear the devices during waking hours, unless engaging in water-based activity. Participants were instructed to clip the pedometer onto the waistband of their clothing or to wear the Fitbit on their wrist like a watch. The participant using the ‘Moves’ application was informed that the application is always running and that they should keep their phone on their possession throughout the day and to use it as usual. Additionally, participants allocated a Fitbit device were shown how to access the Fitbit dashboard, although were not given specific instructions about how to use the available features, as the researchers did not want to influence how participants explored such features.

After trialling each device participants were asked to complete a short questionnaire to obtain a self-reported measure of satisfaction of using the devices (see Additional file 2). This comprised of 10 statements, which required participants to rate their response along a 5-point Likert scale. The ‘Moves’ application questionnaire only comprised of 9 of the relevant statements and Likert scales, excluding a question relating to the comfort of wearing a device. The questionnaire served to provide preliminary information to inform subsequent interviews.

##### Data analysis

Both the GENEActiv and ActiGraph triaxial accelerometers were initialised to record data at a frequency of 100 Hz using GENEActiv PC software (version 2.2, Activinsights, Cambs, UK) and ActiLife software (version 6.11.0, ActiGraph corp, Pensacola, FL) respectively. The raw ActiGraph and GENEActiv data files were processed in R (http://cran.r-project.org) using the GGIR package (version 1.5–7) which autocalibrated the raw triaxial accelerometer signals [21]. Accelerometer wear time inclusion criteria were a minimum of 600 min∙day^− 1^ for at least any 3 days [22]. Non-wear was estimated on the basis of the standard deviation and value range of each accelerometer axis, calculated for moving windows of 60 min with 15 min increments [23], which has been applied previously in studies involving children [24]. For each 15 min period detected as non-wear time over the valid days, missing data were replaced by the mean value calculated from measurement on other days at the same time of day [21].

The self-report questionnaires were analysed to assess compliance with the remaining devices and to inform Phase 3. The questionnaire also provided information relating to the experience of wearing each device, which were explored further in phase 3.

#### Phase 3

Following the action research approach, phase 1 and 2 findings were formative to the methodology used during phase 3. Phase 3 consisted of patient follow-up interviews and health care professional (HCP) interviews.

##### Participant interviews

The remaining participants were invited to participate in the follow-up semi-structured interviews with the PA data collected in phase 2 reported back to patients as part of the interview. Interview questions focussed upon the experiences and opinions of each participant’s acceptability of the PA devices allocated during phase 2. Individualised interview schedules were designed for each participant, informed by participant’s individual responses to the phase 2 feedback questionnaires, and also their activity and sedentary levels as measured by their allocated accelerometry device. In addition, related themes of acceptability pertaining to the influence of others (family and peers) and change in PA (attitudes and behaviours) were explored.

##### Health care professional interviews

During phase 1 participants identified the Clinical Lead Consultant for CF (HCP-C), a Dietitian with special interest in CF (HCP-D) and a Specialist Physiotherapist in CF (HCP-P) as key members of the multi-disciplinary CF team. Therefore, during phase 3, three one-to-one semi-structured interviews were conducted to explore the clinicians’ perceptions of PA monitoring in children and young people with CF. The semi-structured interview schedule consisted of 15 open-ended questions to explore opinions, existing knowledge and experience of using PA monitoring devices, including, the perceived benefits and barriers of using PA monitoring devices and acceptability of using PA monitoring devices as part of routine clinical practice (see Additional file 3). In addition to the HCP interviews, an online survey was disseminated to Physiotherapists across the UK, via regional CF representatives. The survey consisted of 5 questions designed to explore issues concerning the promotion of PA and the feasibility of using PA monitoring in clinical practice. The results of which converged with data from the HCP interviews and are therefore discussed together throughout.

#### Phase 4

A single round consensus technique was used to examine the findings of previous phases, to develop evidence-based recommendations and to direct future research. To reflect on the findings in the context of a real-world clinical setting participation of the HCPs was essential. However, due to their clinical duties, it was not feasible to follow the iterative process representative of the Delphi consensus technique in its entirety, hence the single round technique was adopted. An expert focus group was developed, consisting of five expert-members including HCPs and researchers. The Clinical Lead Consultant and Specialist Physiotherapist in CF who participated in the earlier phases of the research participated in the focus group, unfortunately the Dietitian was unable to accept the invitation to participate in the focus group due to clinical commitments. Two researchers from within the PA field also participated in the focus group, one with expertise in PA monitoring and exercise prescription in children and a second with expertise as a Sports and Exercise Psychologist. The focus group was facilitated by an additional researcher (CH). In total, 5-expert-members attended the meeting, which was recorded using a Dictaphone and transcribed verbatim (Additional file 4).

Members were provided with a working document (see Additional file 5) in advance of the focus-group meeting. The document outlined the session structure and summarised the key findings from phases 1–3. Members independently reviewed the summary to formulate their own opinions concerning PA monitoring among children and young people with CF ahead of the meeting.

Principles of the nominal consensus technique were applied to develop a structured session for the expert-members panel (see Additional file 6). Drawing upon their respective expertise, each member was invited to present 2 or 3 relevant questions and/or statements which they perceived to be priority issues in the use of PA monitoring among children and young people with CF. Once each member had explicitly stated their ideas, a discussion between panel members was initiated to explore and expand upon the issues identified. Equal attention was given to areas of consensus and non-consensus, areas of agreement and non-agreement were each discussed. Priority issues were noted and organised into themes. Panel members also independently ranked each issue, according to their perceived priority significance within the relevant theme and collectively. This was completed after the meeting via email, which allowed members privacy to reflect upon and consider the key priorities and discussion points (see Additional file 7). This was returned to the facilitator who organised the emerging issues (Table 3).Qualitative data are reported following the consolidated criteria for reporting qualitative research (COREQ) checklist (see Additional file 8).

## Results

### Phase 1

Nine interviews were conducted, recorded using a Dictaphone and transcribed verbatim. Interviews lasted between 39 and 94 min (mean = 61.5 min), generating 247 pages (Arial font, size 12 double spaced) and 554 min of audio data. Barriers and facilitators to PA are outlined in (Table 1). Individuals with CF engage in a variety of activities and enjoy PA. Maintenance of health and improved fitness emerged as a key facilitator of PA, with participants recognising the prospective long-term benefits of PA. Participants acknowledged the reinforcing influence that parents/carers, peers and coaches have on PA engagement, in promoting family cohesiveness and normalising the condition. Further to this, the CF Physiotherapist was highlighted as being the most influential member of the CF team in reinforcing PA engagement.

**Table 1:** Illustrative quotes exploring PA perceptions among children and young people with CF

Physical activity participation	Enabling factors		Perceived barrier	Perceived enabler
			Limited PA facilities available locally	Community activities facilitated by private clubs (Thai boxing, football, dance, table tennis, gymnastics) and/or local authorities.
			*“A few more different clubs that do different sports that are around, because there isn’t many.” (P4, pg7, lines 306–307).*	*The swimming centre up the road, and out there is a big Astro Turf…so I’ll usually go there, and the other places are sports clubs and stuff.” (P2, pg7, lines 301–302).*
			Limited time available to introduce and explore new activities	Curricular physical education (PE) and additional non-structured activities such as walking to and from school and play during recess.
			*“Maybe if I’d seen a game or something on the telly, or one of my mates was going to somewhere and said that “it’s good”. “You should come and try it”. I might have a go, but I probably wouldn’t, because I don’t really have time…” (P7, pg10, lines 431–440).*	*“I don’t do any out of school, but in school, apart from PE I do football at breaks and dinners.” (P3, pg3, lines 120–121).*
				Autonomy promoted by independent travel
				*“I can do it [travel] on my own. It’s like I’m not dependent on everyone else to do it for me.” (P7, pg9, lines 361–362).*
				*“It’s either a lift or, because it’s across the [name of local] field across the road, so I can just walk over to that…” (P6, pg8, lines 326–327).*
	Predisposing factors	Am I able?	Participants attributed poor PA-related performance to CF related symptoms, such as breathlessness, fatigue and pain.	CF was not perceived as a barrier to PA per se.
			*“I can do it [PA], but not as good as other people…I get tired quicker than them, or out of breath more quicker. I can do it to a certain point, but then I have to stop.” (P3, pg13, lines 573–579).*	*“…there’s nothing really…wrong with me I can still do it…I’m not stuck like at home or in hospital. I’m out, like able to do anything, really.” (P1, pg12, lines 515–525).*
			For some this results in frustration, anger and boredom;	*“I know just because I’ve got* CF *doesn’t mean I can’t do it [PA].” (P3, pg23, lines 1020–1021).*
			*“Well, it’s a bit annoying, because they’re all doing it, and I’m sat at the side, and it winds me up that I should be able to do it, but I just can’t.” (P3, pg13, lines 584–585).*	*“… I have to do twice as much as my mate, to do what my mates do, so then when I can do what my mates are doing I just feel better, because I know that it doesn’t show that it’s affecting me, and I can keep up with my mates and just do all the exercise and everything.” (P6, pg20, lines 846–849).*
			CF- related illness can render participants incapable of engaging in PA.	Perceptions of current well-being
			*“…Like when I’m ill I feel like I can’t do anything. I’m sitting on the couch and watching TV, and I’m not doing much.” (P9, pg23, lines 1004–1005).*	*“…’cos I am generally quite well, I can do it.” (P7, pg16, line 702).*
				*“I tend to have quite a high lung function, and I don’t really get ill a lot…” (P7, pg17, line 707).*
		Is it worth it?	Some participants reported disliking the experience of pain, fatigue and breathlessness associated with PA;	All participants report enjoying PA. Enjoyment also appears to be inextricably linked to physical benefits gained through PA
			*“(I dislike) The way you get tired and out of breath sometimes.” (P3, pg23, line 1025).*	*“I like it [PA] because it helps my chest and stuff.” (P2, pg19, line 864)*
			*“That now and then it gives you the pains the next day. Like you’re dragging your legs up the stairs the next day.” (P9, pg35, lines 1517–1518).*	Participants also recognise health benefits associated with PA, both in the short and long term.
				*“…it [PA] keeps you active and your lungs clear, and instead of just sitting in hospital or something.” (P1, pg14, lines 631–632).*
				Engaging in PA becomes a normaliser;
				*“It’s just like you’re doing it because you can, and you want to. You kind of feel the same as everyone else for an hour and a half…” (p7, pg19, lines 803–804).*
	Reinforcing factors		Parental support can generate a negative affect;	Family support and encouragement
			*“I did a mile on the treadmill the other day, and Dad was like, “No, you’re going to do another one… (I feel like) I’m going to slap him. Push him off his bike. You do another mile!” (P7, pg12, lines 493–503).*	*...my Mum always like, not makes me go, but if like I’m just too tired, I don’t want to, she’d be like, “Oh no, come on, let’s get out or something.” (P1, pg14, lines 641–643).*
				Peer support
				*“…my friends who knock on for me, they are dead nice because they always ask if I’m ok if I’m out of breath and stuff when I’m out playing footy and stuff.” (P2, pg21, lines 942–944).*
				*“Like one of us wins a race or wins a game or something, I can go, “Oh yes, well, I’ve got* CF*”, and then it’s like pulling a* CF *card…I just find it funny, because they’re like, “Aaaaaaah! She’s done it again”…we have a laugh about it….” (P7, pg13–14, lines 565–574).*
				Significant coaches (conventional and novel, including PE teachers) influence
				*“Well, a mixture of everyone. There isn’t really anyone that influences me any more than someone else… Family, people in the* CF *team, my PE teachers.” (P4, pg11, lines 498–503).*
				The CF specialist physiotherapist was identified by participants (P2, 3 and 9) as the CF clinician who most encourages them to be physically active. Participants also perceive health-related information and advice to be trustworthy and reliable;
				*“I think that it’s good advice, and that I should take it.” (P1, pg11, line 482).*
				Family facilitating activity (e.g. driving to sports clubs or engaging in family activities)
				*“My Mum or Dad would usually take me.” (P4, pg7, line 279).*

Illustrative quotes anchored to the Youth Physical Activity Promotion Model [18]. *‘Enabling factors’* allow individuals to engage in PA, including environmental factors (access to facilities, weather and safety), levels of fitness and skill which are impacted by perceived competence. ‘*Predisposing factors*’ increase the likelihood of an individual engaging in PA: ‘*Is it worth it?* (Benefits and costs associated with PA) which includes attitudes and beliefs and interests and enjoyment of PA. ‘*Am I able?’* (Perceived competence and self-efficacy). ‘*Reinforcing factors*’ reinforce an individual’s PA behaviour (e.g. parents/carers, peers and coaches influence PA behaviour directly and indirectly).

### Phase 2

Participant 3 was hospitalised during the period that they were asked to wear the ActiGraph, and subsequently withdrew from the study. Due to data files being corrupted, data for P2, P5 and P6 was not available for analysis. Compliance and mean wear time were generally good (Table 2).

**Table 2:** Reporting participant characteristics, compliance and wear time from GENEActiv and ActiGraph accelerometers

P	Age Group (Years)	Gender	Allocated device(s)	Compliance (Days worn)	Valid days included (≥10 h·day)	Mean wear time (h)
1	≤10	Female	ActiGraph & Pedometer	6	4	12.5
2	> 10	Male	ActiGraph & Fitbit			
3	> 10	Male	ActiGraph & Pedometer			
4	> 10	Male	GENEActiv	7	6	16.3
5	≤10	Female	ActiGraph & Pedometer			
6	> 10	Male	GENEActiv & Smartphone			
7	> 10	Female	GENEActiv	6	6	13.3
8	≤10	Male	GENEActiv & Fitbit	7	4	23.3
9	> 10	Female	GENEActiv & Fitbit	7	6	14.8

The data presented are from the research devices which are able to provide objective data. A self-report questionnaire was used to assess compliance of the consumer level devices and acceptability, which is further explored in Phase 3

### Phase 3

#### Participant interviews

Eight interviews were conducted, recorded using a Dictaphone and transcribed verbatim. Interviews lasted between 27 min and 75 min (mean = 40.6 min), generating 130 pages and 324.5 min of audio data (Additional file 9). Transcripts were deductively and inductively coded, illustrative quotes were extracted and clustered around emergent themes.

Exploration of participant’s opinions, experiences and acceptability of the allocated devices resulted in the emergence of a number of themes including wear-ability, device feedback and compliance.

#### Wear-ability

Wear-ability of the research-based devices proved to be problematic for many participants. Participants reported finding the “watch-like” designs easier to wear, with some participants citing issues with the fitting, positioning and comfort of the ActiGraph hip-worn device.

*“The Fitbit was basically just like wearing a watch, but the ActiGraph kept getting in the way of when I’d be doing my PE and stuff” (P2, pg4, lines 145–146).*

*“Well, I liked wearing the wrist one because I just liked it more than the other [ActiGraph]…it didn’t have anything for me to do on it, but it kept falling down at my waist and then making my waist really itchy and stuff.” (P2, pg1, lines 36–45).*

*“because, do you know when you put it on the side, it hurts…it did hurt when I was doing some jogging” (P5, pg2–3, lines 62–70).*

Participants trialling the GENEActiv commented on the thickness of the wrist strap, some finding it interfered with activities

*“It was just a bit thick, and it got in my way of doing general activities I do every day, and I had to be careful that I didn’t knock it and stuff like that, and it was quite thick. People noticed it a lot more, and was asking me about it, thinking it was a watch, and then when they actually saw it there was no watch… I was just a bit like, oh...” (P9, pg1, lines 27–30).*

whilst others found it didn’t interfere with activities.

*“it didn’t get in the way of me doing anything, or didn’t prevent me from doing anything either…” (P4, pg1, lines 35–36).*

The visual appearance of the devices often attracted attention from others. This was not perceived to be an issue by some participants, though for some older participants this attention was unwanted, with one opting to tell inquisitive others that the device was a broken watch.

*“They just asked what it was and what it did, and someone asked what the time was with the GENEActiv, and I had to say, “Oh no, it’s not a watch, it’s a blah, blah, blah…” (P7, pg4, lines 180–182).*

*“They’d just ask for the time and I’d get my phone out, and they’d be like, “Why didn’t you just use your watch?” I’d be like, “because it’s this monitoring thing for the hospital”, or like my Mum said, “It’s broke.” (P6, pg5, lines 142–144).*

The consumer level Fitbit with a smaller rubber strap was perceived to be more comfortable than the research-based device and proved favourable.

*“It was a lot more comfortable, the Fitbit. It looks ‘slicker’, it looks smarter than a big bulky thing.” (P8, pg2, lines 74–75).*

P6 did not encounter any problems having their smartphone on their person whilst engaging in PA.

*“Well I usually carry my phone everywhere anyway, so it didn’t really affect me.” (P6, pg12, line 382).*

#### Device feedback

The LED display of the consumer devices was reported as a desirable feature, with participants enjoying the interactive nature of these devices.

*“It was fun to know that I could look back and see how much I was doing…how many calories I’ve burned, how many steps I took, which was good.” (P4, pg1, lines 25–31).*

The information provided by these devices also facilitated self-monitoring for some participants assuring them they had achieved adequate levels of PA.

*“I suppose it taught me that what I was doing, I was doing it right. Like playing table tennis, I was actually doing the right amount of activity that I should be” (P4, pg13, lines 566–567).*

Other participants found the level of information available and interactive dashboard to be overwhelming and complicated, with some opting not to use it at all.

*“It was a bit confusing with too much going on, so it was hard to use.” (P7, pg13, line 581).*

P6 reported that the smartphone based application provided sufficient PA-related information and was simple to use.

*“I didn’t really dislike anything, because it was so simple, and showed you what you wanted to see.” (P6, pg12, lines 376–377).*

The research-based devices do not provide participants with PA-related information or feature a display.

#### Compliance

Some compliance issues were highlighted when discussing results derived from the accelerometry data. Exploration of whether participants remembered to wear their devices or used any strategies to do so revealed that wearing the devices became integrated into their daily routines.

*“I just think it feels like a natural thing, like say you’re getting ready for school in a morning, it’s like you get your t-shirt on, and you put it on basically, like natural.” (P1, pg4, lines 107–108).*

Leaving their device close to where they slept acted as an adequate prompt for participants.

*“Well, I kept it next to where I sleep, so as soon as I woke up I’d put it on.” (P4, pg3, line 132).*

Although, one participant found that despite leaving it by their bedside, they forgot to wear their GENEActiv device.

*“I didn’t forget with the Fitbit, but I could just keep it on all the time…I think I forgot with the GENEActiv because you have to take it off at night, so I might forget to put it on in the morning.” (P7, pg4, lines 148–151).*

Participants opted to remove the devices during activities due to concerns about damaging the devices and/or causing injury to others.

*“Well, usually when we go on the field, we always just run after each other, like taking each other out…but I wouldn’t go too mad on doing that compared to what I normally do… If I take someone down, I won’t just damage whatever it is.” (P2, pg6, lines 255–258).*

*“so you actually might hurt someone else, and you might hurt yourself in gymnastics” (P7, pg4, lines 140–141).*

#### Summary

Overall, participants reported that they enjoyed testing the devices, stating a preference for wrist-worn devices and devices that allowed interaction and feedback. Compliance was generally good, suggesting that the devices were not a significant burden, although P5 did not provide sufficient wear time.

### Health care professional interviews

Three interviews were conducted, recorded using a Dictaphone and transcribed verbatim. Interviews lasted between 46 min and 55 min (mean = 51.3 min), generating 38 pages and 154 min of audio data. Transcripts were deductively and inductively coded, illustrative quotes were extracted and clustered around emergent themes. The online Physiotherapist survey had 30 respondents, 93% (*n* = 28) of respondents agreed that PA monitoring devices could influence their clinical practice. Qualitative data provided are consolidated and discussed with data obtained from the HCP interviews.

Exploration of HCP’s opinions, experiences and acceptability of the PA devices for PA monitoring also resulted in the emergence of a number of themes including, perceived benefits, perceived barriers, and patient and families’ acceptability.

#### Existing knowledge

None of the HCP’s had previously used PA monitoring as part of their clinical practice. HCP-C and HCP-P reported using consumer devices and applications to assess their personal PA levels, though HCP-P did acknowledge concerns about the validity of these devices.

PA is not formally measured as part of routine clinical practice, however an exercise test is administered as part of a patient’s annual review. Whilst the exercise test offers clinically relevant and prognostic information, it does not provide information about daily PA, which was perceived to be a limitation.

*“…the once a year test will prove what they do on that day, and it doesn’t tell us how they do their normal week, cope on a day-to-day week, so we don’t have any kind of measure about what they do, other than subjective, them telling us, “I do, and I’m fine”. You don’t have that information, so I suppose this just gives you over a period of time, if it’s a week or whatever, or two weeks if they were an in-patient, it would give us kind of better understanding of how they’re functioning on a day-to-day level.” (HCP-P, pg11, lines 411–416).*

PA was reported to be informally discussed on an individual basis and advice may be given accordingly although this is not standardised.

*“I will ask them what they do, what sports they do, and how active they are, particularly if we’re having problems with weight gain or weight loss, so say for example you have somebody present to you who’s maybe lost a significant amount of weight, so part of that discussion will be about are you doing more activity, have you joined any other groups, are you doing more planned activity, trying to look at reasons why they might have lost weight. We don’t formally measure how much activity they’ve done and what the energy cost of that is…” (HCP-D, pg4, lines 128–140).*

This reveals that, for these practitioners, current assessments are not sufficient in providing information about habitual PA, therefore individualised interventions to promote PA are limited to informal discussions and generic PA advice.

#### Perceived benefits

Although experience of using PA measurement devices in clinical practice was limited, there was consensus among HCP’s that PA monitoring devices offered a number of potential benefits.

It was perceived that PA monitoring could provide an *‘objective’* measure of PA, which would allow HCP’s and patients to track PA alongside markers of health over a given period.

*“I think, I suppose, trying to help young people to see that actually being physically inactive is potentially having an impact on their health and on their respiratory function, and helping them to see that if they can just increase their activity a bit, then that might have a positive impact on their respiratory health.” (HCP-D, pg5, lines 185–188).*

Objective PA measurement is often used to inform development and evaluation of interventions to promote PA [25, 26] and was highlighted by HCP-C as missing from current practice.

*“…I think we need a more consistent approach across the team, so it will help that. We can formalise more of our interventions with the patients, but we don’t know what those interventions should be, but we can be a bit more robust about it, a bit more sort of systematic, as opposed to just making it up as we go along, which is kind of what we do at the moment.” (HCP-C, pg9, lines 312–315).*

The potential for using the devices as an intervention in themselves was also acknowledged particularly in respect of a motivational aid.

*“I think it would be maybe even a realism about what they do, or how little they do, or it reassures them that they’re doing enough, if they’re doing a lot. Certainly from our experience of monitoring when they do their nebulisers, they have been very very, surprised when they know what they’ve done, compared to what they think they’ve done. So I think that the effect might be, “Oh my God, I thought I was doing lots more than that”, or it might motivate them to do better, to improve.” (HCP-P, pg8, lines 293–298).*

HCP-C concedes that informing a patient of their low PA may have a detrimental effect, rather than encourage PA.

*“…the ones we need to target are the ones who hate physical activity, who find gymnasiums an abomination. And we have a significant number of patients like that, and also quite a large proportion of our young women who are fourteen, fifteen, sixteen, exercise seems to be a non-cool thing for young girls particularly, to do. So those are the challenges we face. Now I’ve absolutely no hesitation to feel that our well-motivated patients will completely embrace this and will love it, and will get a lot out of it, and actually use it in their lives, and that’ll be great for them. But I have extreme anxieties how [sedentary patients] will not in any way be helped by this kind of monitoring. I don’t know. That might not be true. I don’t know what the answer is. If you monitor somebody and show them that the level of activity they’re doing is woeful, then that may motivate them to take a step forward themselves, and try and sort it out. I suspect not, but I don’t know.” (HCP-C, pg5, lines 151–161).*

#### Acceptability

Owing to the perceived benefits of the devices, there was strong acceptability amongst the HCP’s that devices could become part of their routine care, progressing the service and moving with innovation.

*“we could very easily fit it into what we do. We have a very good relationship with our families, a respectful relationship, and we have the capacity within our care programme to sort of slot it in. We have plenty of time. We see them regularly in clinic every two to three months. We do annual review, obviously every year, and we see them in between times. Our families are very engaged, they’re very empowered, they want to do things for the better, on the whole, and they’re very keen to take on, to embrace new developments and new technologies.*” *(HCP-C, pg4, lines 132–137).*

It was reported that use of the devices would be the responsibility of the CF Physiotherapist, (HCP-P, pg10, lines 386–390) and although some scepticism was anticipated from more established members of the CF team, it was felt that the importance of PA would supersede any resistance to the acceptability of the devices.

*“I think generally everyone can appreciate the importance of exercise, but there is some definite variability amongst the team as to how we do that, and how important people feel it is.” (HCP-C, pg11, lines 394–396).*

Aside from the acceptability among HCP’s, the importance of working in partnership with patient families was highlighted.

*“I think we would really need to have families on board in partnership, and it would be something that they would need to see as a routine part of their CF care rather than just something that is a bit different and a bit special.” (HCP-C, pg4, lines 116–119).*

Nevertheless, HCP-C felt that patients and families would embrace the integration of technology-based strategies to improve the management of CF, as demonstrated by the acceptance of other medical device such as the I-neb.

*“Well, I think that the relationship you have with your team, your CF team, is a very close relationship. It’s not always a relationship based on the greatest amount of honesty, and in those cases it’s just a bit tricky, but we’ve kind of dealt with a lot of these issues with a lot of our aerosol delivery devices, and are now able to incorporate data logging, so we have dealt with these issues with the i-neb, and we’ve been able to sort of sit down with the families and the young person in a very open way, and say...‘Just looking over the course of three months, we’ve seen you’ve gone from being a hundred per cent adherent to what you’re doing, to sort of just doing it once a day or whatever, and they’re going, “Yes, it’s a real struggle. Maybe we could go down to once a day”. So working in that open way has worked, I think, and we’ve been able to do some really good stuff in improving adherence in our patients with* CF*, and support them with treatments that they might be finding difficult or impossible.’” (HCP-C, pg10, lines 356–374).*

In order to maintain standards of patient care a number of considerations were suggested as needing to be addressed prior to the implementation of PA monitoring devices including; cost-effectiveness, available resources, staffing, cross-contamination and accuracy of the devices.

*“I think handling data is the key thing, and making sure that the data is readily available in a way that’s readily understood, both professionals and the family.” (HCP-D, pg11, lines 413–414).*

*“I think we need to know what monitors and which are the best…We need to decide what information we need from them as to what benefit they’re going to be. So I think that’s one of the key issues, and I think they also need to be relatively affordable…And cleanable, and from that aspect, so that they can be wiped down.” (HCP-P, pg11, lines 394–398).*

#### Summary

HCP’s acknowledged that overall their current practice does not include adequate measurement of PA or sufficient interventions to promote PA engagement. HCP’s recognised the potential benefits that PA monitoring devices could have in clinical monitoring, informing interventions and motivating patients on an individual basis. Acceptability among the CF team was good, with the Physiotherapist deemed as the individual most likely to be responsible for using the devices. A number of potential barriers will require consideration prior to use as part of routine care.

### Phase 4

Priority issues emerging from the focus group were organised into three themes, patient-related issues, clinical practice issues and research issues as outlined in Table 3.

**Table 3:** Displaying ranking of priority issues identified during phase 4

		Individual priority ranking				Sum total	Collective ranking
		HCP-C (P5)	HCP-P (P3)	Researcher 1 (P4)	Researcher 2 (P2)		
Part 1: Patient-Related Issues							
1	Lifestyle based approach to physical activity	5th	1st	2nd	1st	9	1st
5	Motivation is a key issue	2nd	2nd	1st	6th	11	2nd
6	Perceived importance of physical activity and the data retrieved by the devices	4th	5th	3rd	3rd	15	3rd
3	Experience of CF symptoms during physical activity	6th	3rd	4th	4th	17	4th
7	Importance of fitness over physical activity	3rd	7th	5th	2nd	17	5th
2	Decline of Physical activity	1st	4th	7th	8th	20	6th
4	Importance of clinical versus “field” testing of physical activity levels	7th	8th	6th	5th	26	7th
8	Structured vs. non-structured activity	8th	6th	8th	7th	29	8th
Part 2: Clinical Practice Issues							
2	Education (for practitioners, parents/carers and children and young people)	1st	1st	7th	2nd	11	1st
4	Importance of meaningful feedback	5th	5th	3rd	3rd	16	2nd
1	Role of feedback provided by devices	4th	6th	2nd	5th	17	3rd
9	Clinical barriers identified (cost, resources, time)	10th	2nd	4th	1st	17	4th
8	Issues of compliance raised	3rd	4th	5th	7th	19	5th
6	Sustainability of the physical activity engendered by the tool used in terms of:	2nd	7th	9th	6th	24	6th
7	Importance of accruing 7 days worth of physical activity data:	7th	8th	6th	4th	25	7th
10	Team message is important	6th	3rd	8th	8th	25	8th
3	Testing vs. Monitoring	8th	9th	1st	9th	27	9th
5	Distinction between the use of physical activity monitoring devices as a research tool vs. commercial tool	9th	10th	10th	10th	39	10th
Part 3: Research Issues							
1	Cost	5th	1st	1st	1st	8	1st
4	Children and young people involvement required to inform the research process	1st	2nd	3rd	4th	10	2nd
3	Type of data produced by research vs. commercial devices	2nd	3rd	2nd	5th	12	3rd
5	Literacy and understanding	3rd	4th	5th	3rd	15	4th
2	Issues of compliance	4th	N/A	4th	2nd	N/A	N/A

Expert members of the phase 4 focus group were asked to rank the issues discussed during the focus group meeting in order of priority

#### Patient-related issues

The theme of a ‘lifestyle based approach to PA’ centred on parental influences on PA through their reinforcement and facilitating of PA to promote PA engagement as a ‘*part of normal life’*. This was viewed by the expert members as an important patient-related issue, which should form part of any recommendations and may warrant further research, for example HCP-P noted;

*“Really important is to continue ongoing education on the exercise with the patients and the families, throughout all ages and stages of disease progression, with discussion concerning expectations of the patient, the family and the therapist, and to reinforce that exercise and activity as a lifestyle choice rather than prescription, in order to improve compliance.” (P3, pg3, lines 92–96)* ***.***

The ‘motivation is a key issue’ theme included issues relating to the use of goal setting to promote PA and the impact that device feedback may have on motivation, either positively or negatively.

*“You might get that instant feedback, but you don’t know how to interpret that information correctly. That could have a negative impact on your motivation, or a positive impact, or no impact.” (P4, pg18, lines 691–693).*

#### Clinical practice issues

‘Education (for practitioners, parents/carers and children and young people)’ was identified as a key clinical practice issue and included education around physical activity monitoring and promotion. It was also suggested that this education could encompass an element of counselling.

*“In terms of the education, I think that kind of counselling, it doesn’t have to be a psychologist. It can be, that type of support can be offered in different ways through different roles, through training, through educating parents. It’s not necessarily about parachuting a specific practitioner in. I guess it’s looking for that opportunity, capacity, and the willingness to sort of take on board some of that, in the same way that exercise professionals who work in gyms, they’re not exercise psychologists, but they are very much at the front end of applying principles of exercise psychology in order to make sure that the people they address, whether it’s the cardiac rehab plan or reducing obesity, whatever it is, they’re sort of applying the technique.” (P2, pg19, lines 698–706).*

It was also viewed as important that both clinicians and patients are able to view and interpret the feedback obtained from the devices. This will have implications for the choice of device used and the balance between research data and feedback from consumer devices.

#### Research issues

A priority research issue that emerged was ‘Cost’, with members of the focus group stating that funding would be required to support further research in the area and the clinical use of PA monitoring devices.

*“We need some funding to be able to do this.” (P2, pg23, line 855).*

‘Children and young people involvement required to inform the research processes’ emerged as a recommendation from the focus group discussion.

*“…they are very articulate and able to talk about these devices, and therefore in any future research we should make sure that it’s not just research on children, it’s research with, and alongside, and involve them in the design.” (P2, pg24, lines 896–898).*

#### Summary

The focus group discussion, emergent themes and prioritisation of key issues were consolidated into a number of agreed recommendations and areas for consideration concerning the application of PA monitoring in clinical practice.

#### Recommendations from phase 4

Interventions to promote PA engagement in children and young people with CF should be individualised and involve family members to promote PA as part of an active lifestyle, as opposed to that referred to as prescribed exercise.

Patients should receive education and support alongside the PA data obtained from monitoring devices.

The process of developing interventions and their subsequent evaluations should involve patients and their families throughout the research process who, from the outcomes of this study, are well informed and able to contribute to such matters effectively.

#### Future research considerations

Consideration should be given to the impact that device feedback may have on motivation. It is acknowledged that patients and their parents/carers may respond to device feedback on an individual basis with some having a negative response.

The choice of device will influence a number of key issues identified including, motivation, meaningful feedback, cost and clinical application.

## Discussion

### Phase 1

We employed a socio-ecological model (YPAPM) [17] to facilitate a theory-driven, comprehensive approach to understanding the perceived enablers and barriers among this cohort. Collectively, findings indicate that children and young people with CF engage in a variety of physical activities comparable to their non-CF peers. CF was not perceived as a barrier per se, although reduced exercise capacity and exercise related symptoms were reported to have a detrimental impact on PA engagement, as previously reported in children and young people with CF [27]. As in previous research, there was no evidence of PA avoidance as a result of CF related symptoms in the current study [19]. Rather, PA engagement offered an opportunity to *‘normalise’* their condition by attaining PA levels similar to their non-CF peers. Whilst social comparison in this way can encourage PA engagement it can also be detrimental to PA engagement depending on the level of self-monitoring and perception of differences/similarities in PA ability [17].

Key facilitators of PA engagement were health and improved fitness. Key reinforcing factors were the influence of parents/carers, peers and HCPs who influence PA directly and indirectly. Direct influences include facilitating engagement through pursuing activities together, transport to activities or encouragement [17]. Indirect influences may include affecting predisposing factors, such as attitudes and beliefs (Is it worth it?) or perceived confidence and self-efficacy (Am I able?) [17]. Individuals who report positive PA experiences also report receiving family support and encouragement [14].

### Phase 2 and phase 3

Findings suggest that children and young people with CF are mostly compliant with wearing a range of PA monitoring devices, which is further explored in phase 3.

When determining the feasibility of using PA monitors in clinical practice, guidelines set by Bowen et al. [28] were utilised, the primary areas of evaluation were acceptability, implementation, practicality and integration. Qualitative data supported the acceptability of PA monitoring devices among both patients and clinicians. A number of issues relating to the visual appearance and comfort of some of the devices were identified, with better acceptability reported for wrist-worn devices. Qualitative data obtained from HCPs suggests that implementation of PA monitoring into routine clinical care may be feasible. The data collected from the current study suggests that PA monitoring in clinical care may be limited by practicality. The number of devices which did not provide data due to technical faults was high (4 out of 9), which may limit the feasibility of using these device in clinical practice. However, accelerometers are widely used in clinical research and typically device failure is much lower than reported in the current study [29]. Data from the five devices which did not experience any malfunctions provided usable PA data, demonstrating good participant compliance, as indicated by valid wear time. Demand was not specifically assessed as PA monitoring formed part of a wider researcher study, therefore it was not possible to determine if patients’ decision to not take part was a result of PA monitoring specifically or the study more broadly. Of the 13 participants screened only 9 (70%) were willing to participate, which may suggest moderate demand.

From the follow-up interviews three themes were identified; wear-ability, device feedback and compliance. The visual appearance and comfort of the devices was perceived as important, with wrist-worn devices reported to be more favourable. A combination of these factors may contribute to improving compliance to wrist-worn devices [24, 30]. Feedback from devices was also preferred, as it allowed self-monitoring and provided motivation. Monitoring devices may have a role in clinical practice as a motivation tool, and though there remains a lack of evidence to support clinical use [31], acceptability of the devices was high among HCPs who felt that devices have the potential to provide clinically relevant information which could improve current practice. The data from the current study does not allow comparisons to be made between the acceptability of the consumer level devices and the research grade devices, though only the research grade devices are supported by evidence of reliability and validity [16]. The CF Physiotherapist was identified as best placed to implement the use of PA monitoring devices, although the wider CF team also have a role in promoting PA and supporting monitoring. In addition to Physiotherapists, CF centres are increasingly employing members of staff specifically to deliver exercise testing and prescription [32, 33] and may have additional interest and/or qualifications in exercise-related fields, making them well placed to utilise PA monitoring in the clinical setting.

### Phase 4

Patient related priority issues included a lifestyle-based approach to increase PA and the importance of motivation and family involvement. Clinical priorities included education alongside PA monitoring (for clinicians, patients and parents/carers), and the requirement for meaningful feedback. A wrist-worn device capable of providing research level data, which can be interpreted and analysed by clinicians, whilst also featuring a display for time or user feedback if desired would appear to be acceptable amongst both patients and clinicians (e.g. the ActiGraph GT9X Link, although there are a number of devices available).

The cost of using monitoring devices in clinical practice was also identified as a priority issue. Consumer level devices may offer a lower cost alternative to research based devices and may be perceived to be more ‘stylish’ and less obtrusive. Although beyond the scope of the current paper, the clinimetric properties (reliability, validity and responsiveness) of research grade devices has been reviewed elsewhere [16], providing evidence for their use. Consumer level technology is constantly improving, and a number of devices have become commercially available since this research was conducted which may offer validity comparable to research grade devices, with further improvements anticipated in future [34]. Though agreement with research grade devices is good, validity of consumer devices remains highly variable, which may cause concern for clinical use [34]. A third party software package ‘Fitabase’ (Small Steps Labs LLC, San Diego, CA) has recently become available, which allows Fitbit data to be analysed in a similar manor to the research devices. The choice of device may ultimately be determined by the requirement for user feedback and whilst user feedback is avoided in observational research to control for the risk of participant reactivity, it may be appealing for PA interventions as users can view a range of variables (steps, activity, energy expenditure etc.) [34]. However, HCPs expressed concern that device feedback may be demotivating in some children and young people with CF, suggesting feedback would be best given as part of a package of care delivered from with the CF team, which also included education and support.

### Limitations

A number of limitations are present in the study. Whilst there is a small sample collected form a single centre, the study design allowed for the collection of in depth qualitative data taking a broad perspective with representatives of patients and clinicians. Participants were active and well at the time of recruitment and the self-selecting nature of recruitment perhaps resulted in a sample of patients already motivated to be physically active. It is not known if similar findings would be seen in patients with more severe disease or at the time of an exacerbation. However, HCPs are experienced working with a variety of patients through periods of fluctuating health, which will have informed their responses during the interviews and prioritisation of key issues. Not all participants approached agreed to partake in the study and we did not explore their reasons for refusal. A number of technical malfunctions occurred with the PA monitoring devices, although this is a risk associated with monitoring PA in free-living, the number of issues in the present study was greater than anticipated.

## Conclusions

PA monitoring devices appear to be an acceptable method for the objective assessment of PA among children and young people with CF and their clinical team, though further research is required to determine the validity and reliability of consumer level devices. Wrist-worn devices that are unobtrusive and can display feedback are most acceptable for patients and clinicians.

Experiences of PA among children and young people with CF are largely comparable to their non-CF peers, with individuals engaging in a variety of activities. CF was not perceived as a barrier per se, although participants acknowledged that they could be limited by their symptoms.

HCPs perceive monitoring devices to be a beneficial addition to routine clinical care, although education for clinicians, patients and families is required. Health professionals expressed enthusiasm for PA monitoring, but caution that the information is processed in a positive manner in partnership with the young people.

### Future considerations

This study highlights that whilst PA monitoring has potential as a clinical tool, future work is required to develop a programme of education and support to allow clinicians to utilise the devices, followed by support for patients and families with the aim to understand and use the data obtained for the devices to promote PA engagement as part of an active lifestyle.

A variety of PA monitoring devices are widely available, with each offering a range of different features. Careful consideration should be given to the purpose of monitoring PA in order to select an appropriate device. A device which can be bespoke for individual patients is required to control the use of feedback as a motivational tool to allow for self-monitoring whilst avoiding negative impact.

Further exploration of perceptions of PA throughout childhood and adolescents will improve understanding of how children and young people with CF conceptualise PA. By understanding the factors impacting on PA, CF health care professionals will be better placed to support patients and improve health outcomes. Further research exploring children and young people’s perceptions of PA monitoring devices being used as part of routine CF care may also be warranted.

## Additional files

Additional file 1: Study flow chart.pdf. (PDF 191 kb)

Additional file 2: Phase 2 – PA Monitor Feedback Questions.pdf. (PDF 71 kb)

Additional file 3: Phase 3 - Interview Questions – HCPs.pdf. (PDF 42 kb)

Additional file 4: Phase 4 - Expert meeting transcript.pdf. (PDF 226 kb)

Additional file 5: Phase 4 - Expert Panel Working Document.pdf. (PDF 307 kb)

Additional file 6: Phase 4 Delphi process.pdf. (PDF 57 kb)

Additional file 7: Phase 4 Ideas presented at expert meeting.pdf. (PDF 204 kb)

Additional file 8: COREQ checklist.pdf. (PDF 489 kb)

Additional file 9: Phase 3 - P1–9 Transcripts.pdf. (PDF 670 kb)

## Abbreviations

CF: Cystic fibrosis
CFTR: Cystic Fibrosis Transmembrane Conductance regulator
HCP: Health care professional
MCRN: Medicines for Children Research Network Clinical Trials Network
MVPA: Moderate-Vigorous physical activity
NHS: National Health Service
PA: Physical activity
UK: United Kingdom
YPAPM: Youth Physical Activity Promotion Model

## References

[1] Jeffery A, Charman S, Cosgriff R, Carr S. UK Cystic Fibrosis Registry Annual Data Report 2016; 2017. p. 80.

[2] Zielenski J (2000). Genotype and phenotype in cystic fibrosis. Respiration.

[3] Davies JC, Alton EWFW, Bush A (2007). Cystic fibrosis. BMJ.

[4] Nixon PA, Orenstein DM, Kelsey SF, Doershuk CF (1992). The prognostic value of exercise testing in patients with cystic fibrosis. N Engl J Med.

[5] Almajed A, Lands LC (2012). The evolution of exercise capacity and its limiting factors in cystic fibrosis. Paediatr Respir Rev.

[6] Hebestreit H, Kieser S, Rüdiger S, Schenk T, Junge S, Hebestreit A (2006). Physical activity is independently related to aerobic capacity in cystic fibrosis. Eur Respir J.

[7] Schneiderman JE, Wilkes DL, Atenafu EG, Nguyen T, Wells GD, Alarie N (2014). Longitudinal relationship between physical activity and lung health in patients with cystic fibrosis. Eur Respir J.

[8] Cox NS, Alison JA, Button BM, Wilson JW, Morton JM, Holland AE (2016). Physical activity participation by adults with cystic fibrosis: an observational study. Respirology.

[9] Tejero García S, Giráldez Sánchez MA, Cejudo P, Quintana Gallego E, Dapena J, García Jiménez R (2011). Bone health, daily physical activity, and exercise tolerance in patients with cystic fibrosis. Chest.

[10] Beaudoin N, Bouvet GF, Coriati A, Rabasa-Lhoret R, Berthiaume Y (2016). Combined exercise training improves glycemic control in adult with cystic fibrosis. Med Sci Sports Exerc.

[11] Dwyer TJ, Alison JA, McKeough ZJ, Daviskas E, Bye PTP (2011). Effects of exercise on respiratory flow and sputum properties in patients with cystic fibrosis. Chest.

[12] Swisher AK, Hebestreit H, Mejia-downs A, Lowman JD (2015). Exercise and habitual physical activity for people with cystic fibrosis : An Expert Consensus-Based Guide for Advising Patients. Cardiopulm Phys Ther J.

[13] Jantzen A, Opoku-Pare M, Bieli C, Ruf K, Hebestreit H, Moeller A (2016). Perspective on cystic fibrosis and physical activity: is there a difference compared to healthy individuals?. Pediatr Pulmonol.

[14] Moola FJ, Faulkner GE, Schneiderman J (2012). “No time to play”: perceptions toward physical activity in youth with cystic fibrosis. Adapt Phys Act Q.

[15] Prasad SA, Cerny FJ (2002). Factors that influence adherence to exercise and their effectiveness: application to cystic fibrosis. Pediatr Pulmonol.

[16] Bradley J, O’Neill B, Kent L, Hulzebos E, Arets B, Hebestreit H (2015). Physical activity assessment in cystic fibrosis: a position statement. J Cyst Fibros.

[17] Street R, Mercer J, Mills-Bennett R, O’Leary C, Thirlaway K (2014). Experiences of physical activity: a phenomenological study of individuals with fibrosis. J Health Psychol.

[18] Welk GJ (1999). The youth physical activity promotion model: a conceptual bridge between theory and practice. Quest.

[19] Fereday J, MacDougall C, Spizzo M, Darbyshire P, Schiller W (2009). “There’s nothing I can’t do I just put my mind to anything and I can do it”: a qualitative analysis of how children with chronic disease and their parents account for and manage physical activity. BMC Pediatr.

[20] Hildebrand M, Van Hees VT, Hansen BH, Ekelund U (2014). Age group comparability of raw accelerometer output from wrist-and hip-worn monitors. Med Sci Sports Exerc.

[21] Van Hees VT, Fang Z, Langford J, Assah F, Mohammad A, da Silva IC (2014). Autocalibration of accelerometer data for free-living physical activity assessment using local gravity and temperature: an evaluation on four continents. J Appl Physiol.

[22] Rich C, Geraci M, Griffiths L, Sera F, Dezateux C, Cortina-Borja M (2013). Quality control methods in accelerometer data processing: defining minimum Wear time. PLoS One.

[23] Van Hees VT, Gorzelniak L, Dean León EC, Eder M, Pias M, Taherian S (2013). Separating movement and gravity components in an acceleration signal and implications for the assessment of human daily physical activity. PLoS One.

[24] Fairclough SJ, Noonan R, Rowlands AV, Van Hees V, Knowles Z, Boddy LM (2016). Wear compliance and activity in children wearing wrist- and hip-mounted accelerometers. Med Sci Sports Exerc.

[25] Borde R, Smith JJ, Sutherland R, Nathan N, Lubans DR (2017). Methodological considerations and impact of school-based interventions on objectively measured physical activity in adolescents: a systematic review and meta-analysis. Obes Rev.

[26] Verloigne M, Bere E, Van Lippevelde W, Maes L, Lien N, Vik FN (2012). The effect of the UP4FUN pilot intervention on objectively measured sedentary time and physical activity in 10–12 year old children in Belgium: the ENERGY-project. BMC Public Health.

[27] Moola FJ (2011). “CF chatters”: the development of a theoretically informed physical activity intervention for youth with cystic fibrosis. Open J Prev Med.

[28] Bowen DJ, Kreuter M, Spring B, Linnan L, Weiner D, Bakken S (2010). How we design feasibility studies. Am J Prev Med.

[29] Rich C, Geraci M, Griffiths L, Sera F, Dezateux C, Cortina-borja M (2014). Quality Control Methods in Accelerometer Data Processing : Identifying Extreme Counts. PLoS One.

[30] Freedson PS, John D (2013). Comment on “estimating activity and sedentary behavior from an accelerometer on the hip and wrist”. Med Sci Sport Exerc.

[31] Bradley JM, Kent L, Elborn JS, O’Neill B, Kent L (2010). Motion sensors for monitoring physical activity in cystic fibrosis: what is the next step?. Phys Ther Rev.

[32] Stevens D, Oades PJ, Armstrong N, Williams CA (2010). A survey of exercise testing and training in UK cystic fibrosis clinics. J Cyst Fibros.

[33] Cystic Fibrosis Trust (2017). Standards of care and good clinical practice for the physiotherapy management of cystic fibrosis.

[34] Ferguson T, Rowlands AV, Olds T, Maher C. The validity of consumer-level, activity monitors in healthy adults worn in free-living conditions: a cross-sectional study. Int J Behav Nutr Phys Act. 2015;12:42. 10.1186/s12966-015-0201-9 PMC4416251 25890168

