# Supplementary material for: A formative study exploring perceptions of physical activity and physical activity monitoring among children and young people with cystic fibrosis and health care professionals
Source: BMC Pediatr. 2018 Oct 23;18:335. doi: 10.1186/s12887-018-1301-x (PMC6198445; doi:10.1186/s12887-018-1301-x)
Supplement: Supplementary file 1 — Study flow chart.pdf. (PDF 191 kb) [file 12887_2018_1301_MOESM1_ESM.pdf]

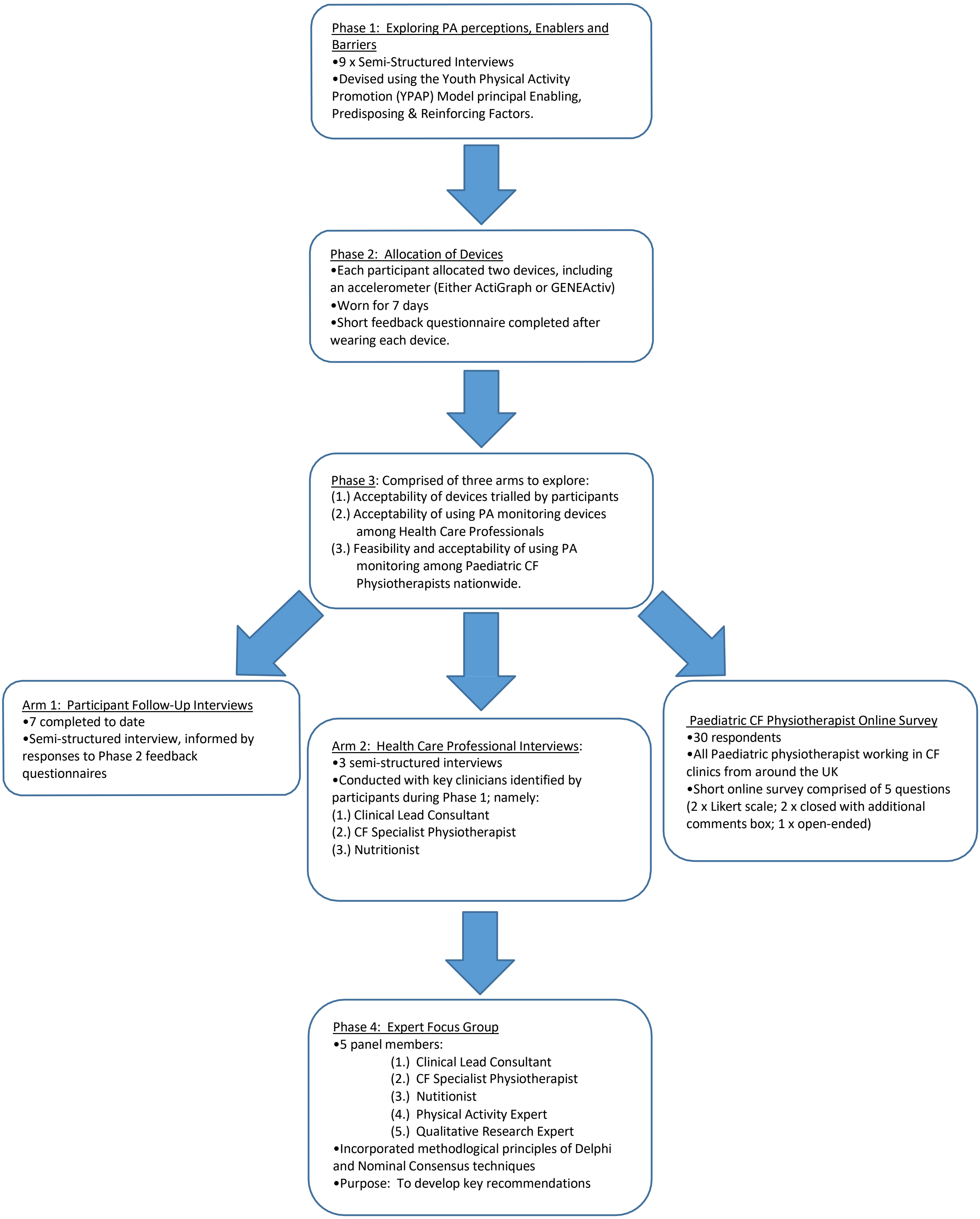
Phase 1: Exploring PA perceptions, Enablers and Barriers
•9 x Semi-Structured Interviews
•Devised using the Youth Physical Activity Promotion (YPAP) Model principal Enabling, Predisposing & Reinforcing Factors.
Phase 2: Allocation of Devices
•Each participant allocated two devices, including an accelerometer (Either ActiGraph or GENEActiv)
•Worn for 7 days
•Short feedback questionnaire completed after wearing each device.
Phase 3: Comprised of three arms to explore:
(1.) Acceptability of devices trialled by participants
(2.) Acceptability of using PA monitoring devices among Health Care Professionals
(3.) Feasibility and acceptability of using PA monitoring among Paediatric CF Physiotherapists nationwide.
Arm 1: Participant Follow-Up Interviews
•7 completed to date
•Semi-structured interview, informed by responses to Phase 2 feedback questionnaires
Arm 2: Health Care Professional Interviews:
•3 semi-structured interviews
•Conducted with key clinicians identified by participants during Phase 1; namely:
(1.) Clinical Lead Consultant
(2.) CF Specialist Physiotherapist
(3.) Nutritionist
Paediatric CF Physiotherapist Online Survey
•30 respondents
•All Paediatric physiotherapist working in CF clinics from around the UK
•Short online survey comprised of 5 questions (2 x Likert scale; 2 x closed with additional comments box; 1 x open-ended)
Phase 4: Expert Focus Group
•5 panel members:
(1.) Clinical Lead Consultant
(2.) CF Specialist Physiotherapist
(3.) Nutitionist
(4.) Physical Activity Expert
(5.) Qualitative Research Expert
•Incorporated methodlogical principles of Delphi and Nominal Consensus techniques
•Purpose: To develop key recommendations
