# Supplementary material for: A formative study exploring perceptions of physical activity and physical activity monitoring among children and young people with cystic fibrosis and health care professionals
Source: BMC Pediatr. 2018 Oct 23;18:335. doi: 10.1186/s12887-018-1301-x (PMC6198445; doi:10.1186/s12887-018-1301-x)
Supplement: Supplementary file 2 — Phase 2 – PA Monitor Feedback Questions.pdf. (PDF 71 kb) [file 12887_2018_1301_MOESM2_ESM.pdf]

LIVERPOOL JOHN MOORES UNIVERSITY

Alder Hey Children's NHS
NHS Foundation Trust

Physical Activity Monitor Feedback Questions

Please read the following statements and circle the answer you most agree with.
If you are unsure about what any of the statements mean, then please ask an adult to explain this to you.

1. I enjoyed wearing the physical activity monitor.	Strongly agree	Agree	Neither agree or disagree	Disagree	Strongly disagree
2. The physical activity monitor was comfy to wear.	Strongly agree	Agree	Neither agree or disagree	Disagree	Strongly disagree
3. The physical activity monitor got in the way of what I was doing.	Strongly agree	Agree	Neither agree or disagree	Disagree	Strongly disagree
4. Other people noticed that I was wearing a physical activity monitor.	Strongly agree	Agree	Neither agree or disagree	Disagree	Strongly disagree
5. Other people, like friends and family kept asking me how much activity I do while I was wearing the physical activity monitor.	Strongly agree	Agree	Neither agree or disagree	Disagree	Strongly disagree
6. I kept forgetting to put the physical activity monitor on.	Strongly agree	Agree	Neither agree or disagree	Disagree	Strongly disagree
7. I did more activity than I normally do when I was wearing the physical activity monitor.	Strongly agree	Agree	Neither agree or disagree	Disagree	Strongly disagree
8. I tried new activities while wearing the physical activity monitor.	Strongly agree	Agree	Neither agree or disagree	Disagree	Strongly disagree
9. I would like to wear a physical activity monitor again.	Strongly agree	Agree	Neither agree or disagree	Disagree	Strongly disagree
10. Getting some information that tells me how active I am each day would encourage me to do more.	Strongly agree	Agree	Neither agree or disagree	Disagree	Strongly disagree

Mobile App Feedback Questions

Please read the following statements and circle the answer you most agree with. If you are unsure about what any of the statements mean, then please ask an adult to explain this to you.					
1. I enjoyed using the physical activity app.	Strongly agree	Agree	Neither agree or disagree	Disagree	Strongly disagree
2. The physical activity app was easy to use.	Strongly agree	Agree	Neither agree or disagree	Disagree	Strongly disagree
3. Other people noticed that I was using a physical activity app.	Strongly agree	Agree	Neither agree or disagree	Disagree	Strongly disagree
4. Other people, like friends and family kept asking me how much activity I do while I was using the physical activity app.	Strongly agree	Agree	Neither agree or disagree	Disagree	Strongly disagree
5. I kept forgetting to look at the physical activity app.	Strongly agree	Agree	Neither agree or disagree	Disagree	Strongly disagree
6. I did more activity than I normally do when I was using the physical activity app.	Strongly agree	Agree	Neither agree or disagree	Disagree	Strongly disagree
7. I tried new activities while using the physical activity app.	Strongly agree	Agree	Neither agree or disagree	Disagree	Strongly disagree
8. I would like to use a physical activity app again.	Strongly agree	Agree	Neither agree or disagree	Disagree	Strongly disagree
9. Getting some information that tells me how active I am each day would encourage me to do more.	Strongly agree	Agree	Neither agree or disagree	Disagree	Strongly disagree
