# Supplementary material for: A formative study exploring perceptions of physical activity and physical activity monitoring among children and young people with cystic fibrosis and health care professionals
Source: BMC Pediatr. 2018 Oct 23;18:335. doi: 10.1186/s12887-018-1301-x (PMC6198445; doi:10.1186/s12887-018-1301-x)
Supplement: Supplementary file 3 — Phase 3 - Interview Questions – HCPs.pdf. (PDF 42 kb) [file 12887_2018_1301_MOESM3_ESM.pdf]

CF Project: Phase 3 Interview Questions – Health Professionals (Feasibility of approaches tested)

Opening Script
Hi my name is Claire and I'm here to get your perspective on the feasibility of using of physical activity monitoring devices among children and young people with cystic fibrosis within your clinical practice. There's no right or wrong answers as I'm interested in what you have to say. Please try and give as much information as possible and please feel to expand on any issues.

The interview will take around 40 minutes depending on your answers and I'll be recording our conversation on a Dictaphone. However the interview is anonymous and confidential and any identifying information will be removed from the transcript of our conversation.

Have you any questions before we start?

1. To begin with can you tell me your job title and what your main duties are with the CF patients?

Experience and Knowledge
Orientating Statement: Physical activity is defined as any bodily movement produced by skeletal muscles that results in energy expenditure and that of which is positively correlated with physical fitness. It is an umbrella term which encompasses different modes of physical activity, such as structured exercise, sport, play, active transport and lifestyle activities.

In this first section I'm going to explore your awareness of physical activity monitoring devices and any previous experience of using physical activity monitoring devices you may have had.

2. Can you tell me about any experience you have had of using physical activity monitoring devices?
   Prompt: Either within a clinical setting or even outside of work.
   What devices have you used before?
   What did you think about the physical activity monitoring devices you have used before?

3. How do you currently measure CF patient's physical activity?
   Follow on: What is good about measuring physical activity in this way?
   Can you tell me what is bad about measuring physical activity in this way?

4. How do you think the use of physical activity monitoring devices compares to the ways in which you currently evaluate CF patient's physical activity?

5. How do you think the use of physical activity monitoring devices could be implemented into your regular clinical practice?
   Follow-on: How practical do you think it is to use physical activity monitoring as part of your regular practice?
   Prompt: For example, in terms of resources?

6. How do you think physical activity monitoring devices could be used to promote physical activity among CF patients?

Perceived Benefits
Orientating Statement: In this next section I'm going to explore what you feel may be the benefits of using PA monitoring within your clinical practice.

7. How would implementing the use of physical activity monitoring devices benefit your clinic?
   Follow-on: Can you give me an example of how using physical activity monitoring devices would be beneficial?
   How about your colleagues, how would implementing physical activity monitoring devices benefit the wider CF team?

8. What effect would the use of physical activity monitoring devices have upon patient outcomes?
   Follow on: Can you tell me a bit more about that?

9. What do you feel is good about using physical activity monitoring devices?
   Follow on: How do you think this compares to what your CF patients think?

Perceived Barriers
Orientating Statement: Next, I'd like to ask you some questions about what you feel may or may not hinder the effective use of physical activity monitoring devices.

10. What do you feel would hinder the use of physical activity monitoring within your clinical practice? How about the rest of the CF team?

11. What do you feel would prevent your CF patients from using physical activity monitoring devices?

12. What do you think your CF patients will like about using physical activity monitoring devices?
    What do you think they will dislike about using physical activity monitoring devices?

Acceptability
Orientating Statement: Thank you for your answers so far, it's helping me to gain an understanding of the potential role physical activity monitoring could have within the service. In this last section I'm going to explore perception of the practicalities of implementing physical activity monitoring within the service.

13. How do you think physical activity monitoring could be implemented into your clinic?

14. Who do you feel should be responsible for the implementation of physical activity monitoring within the CF team?

15. What are the key issues that need to be considered before introducing the use of physical activity monitoring within the regular practice of the CF team?

Debriefing Statement: Thank you for your answers and taking the time to speak with me today. That's the end of the interview now. Is there anything you would like to add? Maybe something I've missed?

Ok then I'll end the interview now. Thank you again for speaking with me today.
