# Supplementary material for: A formative study exploring perceptions of physical activity and physical activity monitoring among children and young people with cystic fibrosis and health care professionals
Source: BMC Pediatr. 2018 Oct 23;18:335. doi: 10.1186/s12887-018-1301-x (PMC6198445; doi:10.1186/s12887-018-1301-x)
Supplement: Supplementary file 4 — Phase 4 - Expert meeting transcript.pdf. (PDF 226 kb) [file 12887_2018_1301_MOESM4_ESM.pdf]

CF Expert Meeting

P1 OK, so as you all know, my name's P1, and we're here to talk about the ideas that have been brought forward from the findings of the CF study. So if you'd each like to take a moment just to introduce yourselves. Maybe we'll start with you, P2.

P2 OK, so I'm P2. I'm here, although it says in terms of a qualitative research expert, I'd also like to offer some comment as a psychologist as well into the discussion, by nature of the results that have come out.

P3 I'm P3. I'm the clinical specialist physiotherapist working with the CF team, and involved a lot in looking at exercise with our CF patients.

P4 I'm P4. My capacity today is the physical activity expert, with a particular interest in children and young people and the measurement of physical activity.

P5 I'm P5. I'm a paediatric doctor, and I look after children and young people with cystic fibrosis.

P1 OK, thank you very much. So perhaps if we go back round in the same order, start off with P2. You've got your ideas.

P2 Right. OK, so as I say, I've looked at this from two perspectives really, from a qualitative methodology point of view, which is how the study in its entirety manifested, and also as a psychologist. Key points for me, to summarise in terms of research moving forward, is that I felt there was significant value to the fact that this study was by design a formative study, and that the outcomes would inform further studies, whether it be intervention or large scale studies. There was a limited recruitment, so in terms of being able to make statements around consensus from the group of patients, that was hindered by low levels of recruitment, but what we have got is rich in- depth information about individual experiences of the children and young people, so that is a strength. In employing both survey and interview methods, we're within the realms of method triangulation, so again in terms of confidence within the data, the opportunity for children and young people to complete the survey and then the interview happen subsequently afterwards to put some more detail on those responses adds confidence to the methods and the data, and also the role of parents. So in some of the interviews the parents were present and acknowledged, though they don't feature within the data set. That was, again from a confidence point of view for the parents and their consent, and my perception is that that provided good compliance and engagement, and we dealt with the data efficiently by way of what was offered by the parents, and almost cleaned the data to make sure it didn't contaminate the children and young people's responses. From a psychologist's point of view, I feel there is scope to look at, for any future interventions, some scope around looking at how the children and young people interpret negative symptoms of activity, and that some intervention by a specialist could be useful to support that. I also think that the devices that they used, that there seemed to be some feedback from the children and young people around the immediacy, the interactive nature, and also the individual nature of the responses, almost to the point where we might look at choice, if you're going to use these devices in the future that there could be a menu. And my last one now, sorry, and again, reiterating the point from the qualitative research about the role of others, whether it be the CF team or the parent, and

the sort of training and intervention work. There needs to be sort of a team approach, rather than typically we might look at interventions to target children and young people, and that for me is different to other paediatric interventions that we've done before.

P1 OK, thank you, P2. Would you like to go next, P3?

P3 Yes. I've just got some comments about the different points of the certain phases, and some of the way the subjects perceive themselves as being highly active. Again, I'd like to know a little more kind of how that maybe compares to reality. How did they define their own level of activity? Did they count how many football sessions they had a week, or was it organised activity or self-monitored things like that, and what was their expectation of themselves? Did they fulfil what they thought, or did they fall short? In point number four, you mentioned the unpleasant symptoms, and about breathlessness hinders capacity. This is interesting, and it kind of makes me wonder whether our children and adolescents identify breathlessness as part of having CF, as opposed to being normal, as a normal part related to exercise, and that shows me that some didn't cope very well with the feeling of being short of breath. Another interesting one was they perceive themselves as less able to do the same level of activity as their peers, and this is a bit disappointing to me because we do a lot of education and activity, and we promote it from diagnosis. So we personally would have a high expectation they'd have good excellent lung function, and therefore should have little impact on their ability to exercise. So I just wonder is that perception real to them, or is it something they learn, or is it an approach that they develop as having been given the diagnosis, and again, do they challenge themselves less maybe, or have lower expectations than others? And again, it kind of tells me that we need a better model of self-perception and education. Again, looking at the similarity between the parents and the participants, again, thinking back to that point six, is it, something that the parents' anxiety is projected onto the child. Again, looking at the results, we didn't relate anything back to age, and we had looked at primary and secondary age group, and is there anything we can pull out from that aspect. So, just to finish, in choosing the device, I think, like, P2 said, I think patient choice is a really important factor. They like the feedback, they like to see their results instant, preferably. The comfort of the device was obviously important to them, and in some cases it being discreet, although maybe trendy might be a more...A couple of words on the physio survey. I was just interested to see what people did around the UK, field tests.

P2 Yes. OK. Sorry.

P1 No, go on. That's fine.

P3 Exercise tests were uses, field tests were used in the majority, twenty-two out of twenty-six, and only four used the very specific CPET testing to use VO2 max. So I think best practice would be as a practical application as opposed to an annual test for individuals, which highlight concern for us. It would be really useful to support patients after an acute exacerbational following exercise tolerance test that shows poor deterioration tolerance, and it would inform us of the patient's actual level of activity, rather than relying on patient report.

P4 Where did you get that thing about the CPET testing?

P3 It's in the physio survey.

P4 Did I miss that?

P1 Oh no, that wasn't on there.

P2 Yes, you can have a look at those if you want. Those are the physio survey.

P4 Did I get those?

P2 No.

P4 All right. So you're talking about something I haven't seen. That's interesting. I'd like to have a look at the physio survey.

P3 Can I have two more sentences?

P1 Yes, of course.

P3 I think it's important to let the patient choose after being shown the alternatives. Really important is to continue ongoing education on the exercise with the patients and the families, throughout all ages and stages of disease progression, with discussion concerning expectations of the patient, the family and the therapist, and to reinforce that exercise and activity as a lifestyle choice rather than prescription, in order to improve compliance.

P1 Thank you.

P4 OK, well, the teacher in me couldn't resist producing some paper, so if you want to look at the stuff that I'm going to talk about, I'll shove it on there. You're very welcome to do so, and I'm going to resist the temptation to pick upon a lot of the things that you've mentioned, P3. I need to stick to what's on here. So I'm just going to go through each phase. So I guess the stuff on the first one was the small sample, and the kids describing themselves as being high active, so we've got to just be conscious of that perhaps there's a degree of kind of selection bias in the children that we're referring to. Maybe they're not typical. The second point is about this issue of the CF symptoms being exacerbated during physical activity, which hinders participants' capabilities to then be active in one of the points down here, and then there seems to be some normative comparisons with peers. So that kind of got me thinking a little bit about this thing about perceptions around competencies and self-efficacies, and some of the psychological backdrop to that. And then the last point there is provisions for activity to allow kids to socialise and experience relatedness with other kids. So there's some crossover there, so we've couched the research in this model at the top, promotion model, and these predisposing factors here are these kind of self-perception-related things which are very important for activity, and that kind of is borne out a little bit there, and that INAUDIBLE (11:46) self-determination theory kind of model where being related, or having relatedness in conceived competencies is an important part of that. So there's some kind of psych theory which is underpinned a little bit. This next little line diagram is about, well,

we've got this activity behaviour here which comes out of phase one, and we often think of activity as having these different domains. There's frequency, intensity, time and type, intensity being quite important. So there's some links in the results to being active related to being fitter, and having health and wellbeing, which is a positive outcome. To improve fitness we need to engage in vigorous physical activity. In the results it then kind of said, "Well, being fitter mitigates the symptoms of CF, but also says that having the symptoms hinders the participation of vigorous physical activity. So it's almost a chicken and egg scenario there, the way I kind of read that, and it'd be interesting to see thoughts on how we can maybe try and address that if that's possible. The next one is the second phase, which was some of the physical activity data. I could spend a lot of time on this. I'll just briefly say that in the literature there is not consensus on the best way to find physical activity from accelerometry, which these bar charts are from very often, and so there are a lot of factors around different protocols, different monitors, different placement of the monitors. So that meant that maybe those data we see from the kids in the study are not representative, that a mean of a hundred and ninety-two minutes over three hours per day is far too high to be realistic. There were some outliers here, so at the bottom right, three or four hundred minutes a day of physical activity, that's definitely an anomaly. The middle one and the one on the left are a bit more typical, actually. One's from a wrist-worn accelerometer, one's from a hip-worn one, probably a little more representative of what we'd expect to see. So we can't put a lot of trust in the data, if you like, just from the few children that wore the monitors in this particular...Right, I've got one more, which I shall just finish up on. Now this was about...There's some really good information here in phase three, and I've tried to summarise it a little bit. Two points for me. The health professional data and the physio data. Physical activity monitoring is used, the term is used in the same way as what we term exercise or fitness testing. So there seems to be some disparity there between understanding of what activity monitoring actually is, as opposed to testing for levels of performance or capability. So that's about a bit of an education from a professional's point of view, to kind of separate those processes, and the box at the bottom was just to think, "Well, what's the purpose of monitoring activity?" It might depend on who it is for, so as a motivational tool, which I guess from the participants, all the things that have been mentioned about, feedback, immediacy, interaction, aesthetic, comfort etc, which are quite different from a research point of view, where we're more concerned about being accurate and not being tampered with, and having lots of great data and then applying that. So it's maybe bridging the gap a little bit, and I think the clinical outcomes. That's just my perspective, maybe bridge those a little bit better, I guess, so involves talking about that in a bit more detail when we get chance, P2.

P2 Right, you're on.

P5 Super. Well, well done for completing the study. I don't think I've ever spoken for three minutes on any subject in my life ever, but I'll try.

P4 We've all failed so far.

P5 My impression was that you haven't selectively chosen children who were doing a lot of activity. I thought there's been a sort of purposive selection of some children who weren't, so I'm quite kind of buoyed with the amount of activity they were doing, and also with the engagement they had in the study. I think that was very good, and sort of encouraging, and lots of really encouraging things come out of this project. The way that the health professionals feel it's really important, and the physiotherapists say this is a really key element of what they're trying to do is physical activity monitoring, and we're not doing it in a very consistent or valid way. I find the whole thing has been really interesting, because we've been taken over by Nike and Apple and everybody, and this whole area has become sort of very trendy, very hippy, very American. I mean, I go to America, and I see all these guys wearing their devices all the time now, and that's really interesting in that there's been, because some of the statements talked about stigmata, and I think that's a really big thing for our patients, and it's been a bit upsetting in a way to read about, like they didn't like being asked about it, and I think that's a really key element for the patients. But actually, that's been overtaken, in that these devices are now going to become, well, are also quite trendy and a part of life. So that's kind of encouraging really, but it still makes this work very important. So the questions I have, and I think you've raised compliance, which is a key element. So what I have in my mind is a vision that all my patients do lots of exercise and they have a lovely time, but I'm obviously deluded, and we know that there are some who don't do any exercise, who actually find exercise quite abhorrent. So I have an anxiety about actually stigmatising them, making them feel guilty, making them do something that is a burden, a complete burden, and actually, if you make it something that they've got to do, even the children that like exercise will find it a burden. So I'm in a difficult place at the moment. I don't know the right way forward. I think this has given me a lot of pointers, but I think what the bespoke model that somebody talked about, about individually assessing individuals is really important, but on the other hand I don't want to discriminate, so I want these things to be available to everybody. So it's not just going to be our very well-off, well-to-do patients from mid Cheshire who have all got these bracelets and are doing the right things, and all our less well-off patients from more deprived areas are excluded and discriminated against. We've got to make sure that doesn't happen, but we've got to have a firm evidence base to be able to go to funders and say, "Look, we need to not only monitor the physical activity of the patients, but also their whole family", and I think, was it you that said something about that, P3? I think that's really interesting. I didn't quite understand what congruous between parental and participant. We can talk about that in the discussion. So have I gone over?

P1 Yes, just a bit.

P5 Oh, I think big brother as well. I haven't really got to the point where I understand how they feel about us knowing how much physical activity they're doing. I think we've made inroads, but I still don't know whether they're comfortable with that, and I think that's really key, because we've got to be working in partnership with them.

P1 Thank you very much. OK, so there's been quite a few key issues raised there, all interesting, some convergence, some not. Who'd like to start the discussion?

P5 What did that mean? Congruous between parental and participant physical activity-related was reported congruence.

P1 Yes, so they both agreed, in terms of...

P5 Physical activity-related beliefs. So if the parents had good beliefs with regard to the need, then the children were the same.

P1 Yes, they pretty much agreed. They both seemed to come from the same page, the parents and the patients.

P5 And did you find the other was well, that the parents who weren't so supportive, because we have quite a few of those, but maybe they weren't in...

P1 In the sample, no, I don't think. I think they were all quite positive, and all quite supportive of the kids', or the children's levels of physical activity, and quite encouraging, really.

P4 Yes, I think I think I might have had the parents demonstrate that positive perception.

P1 In what, sorry? I don't quite understand.

P4 So it could be, we might share a belief, but so then I might go off and I might be a perspective person, or it might be that I drive you to that sports club, or it might be that I give you lots of encouragement or feedback, or... did you get a sense of how that was manifested?

P1 Yes. So I got a sense that the parents did encourage the children and young people to take part in physical activity, that encouragement could be, and it was all, I suppose this is the point, isn't it? It's individual to each family. So you might have a family who you may describe as being a lifestyle...they've sort of embraced the whole physical activity lifestyle, so that as a family they'll go off and they'll go walking, or they'll go on days out which involve some sorts of activity like bike riding, or maybe go to the gym together, but then the other scale, you may have parents who aren't that keen on physical activity, but recognise the need for their child to be engaged in physical activity, so would encourage them by driving them to activities, encouraging them to go and play out, and even for people where, for whatever reason, structured activity may not be a particular feature in their physical activity profile, if you like, or what they engage in, it could be just, you know, "The weather's lovely out there, don't be sitting there on the Xbox, get outside, go and play football with your friends, go and knock on your friends, go for a walk", that type of thing. So there was the whole, I suppose, spectrum really.

P3 I think that's perfect. I mean, that resonates with what we find with interventions with families generally, is that there are parents who support physical activity, those that model it, those that go and engage in it and immerse themselves in it fully, and then those that are more passive and say, "Oh yes, I know it's good for you. I just can't be bothered, but I'll take you to swimming, or I'll take you to karate". So for me, one of the things that...

P5 And actually, quite a few people won't even take, because it's an effort on their part, so...

P3 Often cost as well, isn't it?

P2 Yes. But I think one of the things that came out for me from the whole study was that actually, a lot of what has been reported we see in mainstream interventions with non-clinical populations through schools. So we see exactly the same, and in the same way, it might be helpful for parents to be reinforced in that way, that that's perfectly normal. I guess they're taking their point, their normative reference from having a child with cystic fibrosis, but we see that in a lot of the studies that we're engaged in, that parents have this. You know, do they not get involved, or there are concerns around that, and helicopter parenting, and these sort of dance Mums, and all this type of thing, but there's lots of different ways to parent your child other than activity.

P5 But they're like the facilitators, aren't they, the sort of, we take them here, we take them there, join this club, join that club? But I think we're talking just more about the sort of parents showing doing the activity themselves, and the children looking at them as role models.

P4 Well, I guess there's one example. Yes, because it's this kind of indirect and direct kind of positive reinforcement of physical activity, and it's probably the more indirect kind of subtle messages which builds up those kind of competencies, those perceptions, which probably have more longevity in terms of having this effect, rather than Dad goes running, therefore it seems a good thing that I should do that, you know what I mean? So the strength of that kind of reinforcement isn't as good, as P2 said.

P5 ...Go out and do something. It never works. If they're on the Xbox and you go, "Go and do some activity".

P1 They're not going to do it.

P5 But if they see what is a normal part of your life is playing squash or cycling to work or going out running, then they'll go, "That's normal", and then eventually they will, when they're of an age where they're cogent, we see them sort of blossoming at about seventeen, eighteen, quite a lot of them sort of go, "This is something that I need to do".

P1 I think that, picking up on that point, there are a couple of families who do really stick out the way you're saying, issues of reinforcement, and it's those, I suppose modelling to a certain degree, the way some families where the parents, where they're particularly active and regularly engaged in activity of sort, again bike riding, going the gym, and even the young children, young people with CF, they would accompany them also. So they might all jog down to the gym together, and then go on a piece of equipment together. There was a couple of families.

P5 We encourage that, don't we?

P3 Yes, I was going to say. We've got families whose parent who won't be particularly active, but will join the gym and go swimming with their child, just so that...

P5 The driver is to engage the child, because that's interesting...

P3 That's right. And they're the ones that really have difficulty, and I do feel that each child has their own independent either joy or hatred of exercise anyway, whether they've got CF or not, just going by your own book, but actually, the ones that are quite inactive, you've got parents like that who will, you know, we have got them, maybe not in the study, but we've got them, that will actually go to that level to actually swim with them, or go to the gym so they can go swimming, or...

P5 We do have some where it goes actually to extremes, and we say to them, "Activity is a really key element of having a good and healthy and long life with CF", and some of them take that on with spades...

P3 And then actually...

P5 And then when they come to claim, you say, "What are you doing?" and you just go, "Oh."

P3 And their weight's gone down.

P1 Yes. So it sounds like you're saying it's a balance.

P5 That's not very often.

P3 But there are extremes.

P5 I do worry a bit about them, because sometimes they burn out, don't they, and teenage rebel, and then stop doing activity. We've had a few of those, who have been very, very active at junior school, and then stop doing any sport or...

P3 Yes, particularly GCSE year, kind of that year, because academic-wise, you just haven't got time, and we've seen lung function drop during that year.

P5 But the ones I worry about the most are the ones from really chaotic households, deprived circumstances, where people are coming in and out of the house. We can't even begin to imagine what their lives are like. We do have a lot of patients like that, and they can only exercise by sorting it out themselves, and so there is a real discrimination against them.

P1 Where do you see physical activity monitoring as it's been tested within the study helping those kind of people out?

P5 Well again, what you need to do is help their resources, to be able to structure doing stuff in their lives, and they quite often do go out and find things, don't they?

P3 Yes.

P4 It might be to the point where the extent to which they value that behaviour as being important to determine whether or not they will engage in any role in a form of monitoring. You could give someone a piece of equipment, but if they don't attach importance to that, then they're not generally motivated to wear it, and what maybe connects me a little bit is this whole idea of lifestyle approach work, and we see it a lot in our school-based kind of interventions, where we look at well, there's a school day, these are opportunities for

physical activity as opposed to certainly being seated, and they don't have to be structured or exercise, but they can be quite incidental, they can be quite social, and it's about building those in really, and it's almost, I mean, apart from the references to CF in some of the documentation here, we could almost be talking about any children really, in terms of some of the...

P5 I think there are a few key things in here, P4, that do worry me. Their perception, particularly what P3 raised, that they are not normal, and I am really shocked by that, and I don't know whether that was a common theme or just one or two people saying.

P3 See, we wondered that, whether that was across the board, or actually that was one person kind of...

P1 I think there was an acknowledgment that the young people are aware that they've got CF, and they know that it's a chronic condition, and it's something that they are aware that they have to, I suppose, assimilate into their lives, but not let it define them. That's what I got as a main theme. So there's a recognition there that they have a condition that does make them different maybe to some of their peers. However, it doesn't define them, if they're right, maybe clarifies it a bit better. So they're aware that running a hundred metres may be more difficult to them than their non-CF friends. However, it doesn't stop them from necessarily doing it. So it's almost like an acknowledgement, but not...

P5 The vast majority of our children, we would expect them to be as good, if not better than their friends, because we have encouraged them, and they have very good respiratory capacity. We have a few, we have a very small cohort, who have quite significant lung disease that does impact on their capacity to do cardio-pulmonary exercise, but they shouldn't hold them back from doing it. I mean, quite the opposite. In fact, some of them are so driven that they are actually very good at doing shuttle runs or whatever, despite the severity of their disease.

P4 Would it mainly manifest itself, depending on the intensity of the activity and the duration to which they were doing it for?

P5 P3 could answer that.

P3 Yes, I think it might, actually. Kind of the longer the activity, they get tired. You know when you do one of the exercise tests, the modified shuttle, it's not breathlessness that necessarily stops them. It's actually their legs are tired, and it's kind of working out really, whether they're unfit, deconditioned, or actually it's not always...I mean you've got some, obviously, but it's a very, very slow start to the test, very slow, so it's an endurance test, if you like, because thirty-five minutes, thirty minutes, I think it takes to complete, and we do have some that have completed it. We have that level of fitness, but a lot of them, the younger age group or mid age group, it's their legs, they're physically tired.

P4 Local muscular, kind of?

P3 Yes.

P4 Do you do any ratings of perceived exertion during the test as well?

P3 Yes, we do. We do the Borg scale, and we do the sliding, what do you call it, the...

P4 The Omni scale? One to ten?

P3 Yes. We do that as well.

P4 So they're getting, as you'd expect, they're getting when they drop at the test, when they finish the test, are they scoring...?

P3 High.

P4 Yes, so back to...

P3 Yes.

P4 OK.

P3 So we do the exertion, the dyspnoea and the linear scale at the bottom, yes.

P5 There are a couple of centres around the country that actually formally measure CF, don't they?

P3 Well, that's what I was interested in, and there's only the four there that do that.

P5 That's quite a few. I mean, and you can probably see why they do it, and for the engaged patients, they really love it, because they like to know what their VO2 max is, and how they could improve it.

P3 You know, in everyday life, I see the field tests as being more important, because you want it as their normal activity, what they're coping with functionally, which I think those, you know, the step tests, you've got stairs everywhere, and kind of the shuttle, I think they can be more realistic to me than looking at, unless you've got an athlete or you're looking at that. So I don't know, we haven't got that facility, and many places don't have the facility for VO2 max. I mean, we shortly will, won't we? And what we do with that, but the field tests are what the majority of centres have got, because you can do that in a very small space.

P1 How do you feel the patients will respond to the VO2 max?

P3 I don't know. I think the good ones will love it, because it's another test.

P5 It's quite an intensive thing for them to do. They are competitive.

P3 Yes, competitive, and I mean, we don't encourage competitive between others, but we do encourage competitiveness within themself. They will always, when you do the shuttle test, they will always say, "What did I do last year?" And their motivation was to do better than last year.

P2 I think that links in to this notion of feedback and immediacy of feedback. They strike me as a group that they get a lot of information about their condition, and they're very knowledgeable. I'm just looking at the literacy around in the transcripts of them talking about their condition. They're very articulate, and therefore that for me was something that came out, that they want that type of feedback, so they can do the VO2 max and get immediate feedback. The same from the devices. And I take P5's point about for those that maybe have lower scores, that it could demotivate them, and therefore perhaps there needs to be some individual counselling around employing things like this. In the same way, I guess as a parallel, things like GP referral schemes, that when people start exercise or cardiac rehab, and there is some behavioural support around that in order to ensure from a self-perception point of view, from a competence point of view, that that's reinforced, and they understand how to deal with that feedback, rather than it just being a number, that there's some interpretation of that, and I don't know whether from a capacity point of view, that that would be reasonable or practical to do from yourselves as clinicians.

P5 Oh God, yes. I mean, so you ask the question, there's a number of questions I have here relating to that, but also other things. So you asked about how they would assimilate the information from a VO2 max. I'm not even sure I know how they would assimilate the information from a physical activity monitoring from this, I don't think, yet. But you're going to have, you can set them goals, and we would set them goals, we're constantly setting them goals, but if you set them goals and they could achieve those goals, which is great. We set them more goals, and so on, or they could nearly achieve those goals and not do to badly, or they might fail completely because they're lazy, or they might fail completely because they haven't got the capacity and resources to actually do what they've set themselves. They might have set those goals themselves. So I don't know how each of those scenarios is going to affect them, benefit them, help us in the partnership with them. So that's a key element for me. I want to know that if we're going to use this in the formalised way, what impact that will have on the patients, all those different outcomes, and I think that's the key, really.

P2 I'd say from a parallel, in terms of GP referral, that's really important, the regular contact with somebody, an exercise specialist or a psychologist, that they can help with that when they don't meet milestones, and help them to deal with that. It's not just a case of setting them on a programme, and research suggests that when you add behavioural counselling in, rather than just exercise prescription or exercise monitoring, that the effects and the uptake and the sustainability into a lifestyle choice, and they become more autonomous, and that that really helps. So for me, I agree. We need to look at what the impact of that feedback. They can pick a device that gives them that type of feedback. They can pick a device that doesn't give them that type of feedback, if we had a menu of ones that are available, looking at different types of devices. But I guess it's an individual choice that requires some kind of counselling or support alongside that, so that they understand how to be able to deal with that, so what happens if we don't hit a milestone this time. Because certainly, throughout the interviews, they are very good at goal-setting for children and young people, so that's obviously coming through, that they're set goals, and they're very knowledgeable at understanding around that condition. So it seems that the foundations are there, but the choices around the devices, and how we deal with not hitting milestones.

P5 I think achieving the goals is almost harder than not achieving the goals, because then where do you go? Where's the next? I mean, not achieving the goals, then we can start to investigate why they're not, why their physical activity is not improved, why it's poor. We can look at the causative mechanisms. But if they are actually, they set themselves a goal and they achieve it, then where do you go from there? It's sustainability, and that's very hard. So...

P4 You'd expect activity to drop through those years anyway, so actually, maintenance and even decreasing the decline seems a positive outcome in terms of activity interventions. And I think stuff like the goal setting, and in one sense, it could be a number, so you have so many steps per day or so many minutes of activity, but some of the devices, if they look at patterning over the day, then you've got a bit of a better feel for where the activity's occurring, and then you're a bit more informed about how you can intervene or set goals. It might be a goal about what you do after school, for example, rather than a global be more active.

P2 Yes, it could be an active travel type of approach.

P3 Yes, like walk to school instead of getting in the car.

P4 So the level of detail from the device actually could be a mechanism by which you can actually provide a better level of feedback, and also conceptualise that in terms of, "Well, you've done X number of minutes of activity today. Actually, that compares nationally for mainstream children like this", because most children are relatively inactive, and so those low expectations which you referred to, P3, actually they might be over-egging that a little bit, because most kids aren't that active per se. Well, they might see themselves as being less active because of the condition, where the reality might be quite different, so it's almost like saying, "We've got some mainstream kids here. They wore the monitor. This is how active they were, this is how active you were", and the line might be, they might always think it'd be like that, but it might not be.

P1 Can I just take it a step back a minute? You mentioned, P3, about, and it ties in with what P2 was saying about letting the patient choose the device, and showing them the alternatives. You said you had a little bit more to say about that earlier on.

P2 Well, it's just, I think...I've forgotten what I was going to say.

P1 Oh, sorry if I put you on the spot.

P5 This sort of bespoke approach to sort of each individual patient developing their own individual programme is very sensible, isn't it? And it's kind of what you do outside of CF that is what would be recommended, isn't it? So...

P3 They've got to be interested, and it's got to be, because, I mean, you have families who'll go through every sport, and you think, well actually, the child will choose what they want to do, and you'll get motivation to continue, and sustainability with that, because they enjoy doing it, but if they make them swim or make them do something, and you'll find that some

families literally go through, you know, they've got tennis one night, and swimming the next. You know, there's kind of, you think, "Crumbs!"

P4 But then it's that flip in that, from an education point of view, of what is physical activity, and it's not just a sport model and a structured model and a coaching model and a performance model. Looking across that typical day, and those opportunities, and going back to that as a start point, and do you walk to school? Or what do you do at playtime, or when you get in from school, what do you do? And it can be those things, as simple as go and knock for your mate over the road, and because some kids will naturally gravitate more towards that, while some children will gravitate more towards a more kind of performance-orientated type of activity.

P2 I know what it was I was going to say. It was, you know your kind of, your organised and structured kind of activities? That period that they're at football training, or they might only have a short period of time they're actually really active within that, as opposed to cycling to their mate's house up a hill on a bike. You might actually find that they're actually doing more by doing that, rather than paying for education, and just looking at exactly...but you would see the intensity that they're doing through the devices, depending on what do you want to see from...how do you choose the device. You've got to have useful information that you can get off that.

P3 From a family point of view as well. So as well as sort of school-based interventions, we've looked at family interventions, and I think parents have that perception of, "Right, I want my kids to be more active. Right, they're going swimming to swimming lessons, they're going to go to rugby training on a Friday". Certainly a lot of the mainstream research, they don't understand the chief medical officer's guidelines on physical activity. They think it's you've got to be sweating and running round a field, not about, well, that could be accrued through discreet periods in the day, being more active at playtime, walking to school, playing out after school. So I think that there's some education around that from the parents as well, with this perception of normality, because they think that, as I say, they're just misinterpreting those guidelines and that individual nature to, ok, so let's look at you as a family. What opportunities are for you as a family from an intervention point of view? What can we do to support your family unit? And certainly other studies we're involved in is looking at physical activity monitoring for the whole family.

P5 I would love to do that, yes. But you have to have, if you're going to fund something like that, you know, we need twenty thousand pounds for the whole of our clinic probably, to provide them all with appropriate devices. You'd need to have a very good evidence base to support that sort of approach.

P1 So P4 mentions contextualising the physical activity, tying in with what you said about structured activities not necessarily achieving the intensity. Would there be any sort of benefit in that?

P3 Well yes, I think, as P2 said before about, throughout the day, school day, weekends, kind of spreading out...

P5 Using opportunities.

P4 From your point of view, is an improvement in absolute fitness very, very important, more so than improving general activity?

P5 The data from the adult studies suggests that an improvement in actual fitness is associated with better quality of life, better survival, formally measured, and that correlates with level of activity, so I see what you're saying?

P4 Well yes, because that's where the intensity element of sort of structured physical activity like the sports club in theory, if done well, should result in a higher intensity, whereas maybe the more incidental stuff is more probably moderate and light intensity, and so if an important outcome is to improve cardio INAUDIBLE (47:00) fitness in intensity, and the volume needs to reflect that, perhaps rather than an emphasis on more generic moderate and light activity, but I guess the trade off there is one of motivation, magnification and all those kind of things.

P3 I think, even the less intensity, the changing from an airway clearance point of view, changing body position, changing the way you breathe, that all has benefits as well, so in some of them that we are using trampoline particularly, you're using it in a different way to actually promote, because some might not use, they won't do their physio, they won't do their normal airway clearance, but they will do exercise. So actually, there are things that we can guide them into. You do change those ventilation rates with body position, and actually, that will help with their airway clearance, not necessarily their fitness.

P5 But what you're trying to say, P4, that maybe that can be concertinaed down into sort of five minutes really, really vigorous exercise.

P4 Well, there's high intensity interval training, stronger evidence base in adults, so a big debate whether that is really a public health kind of strategy in terms of physical activity and health, but there is some evidence in children that those approaches can be effective, and certainly, yes, those thirty second whatever it is, sprint or something very, very strenuous, short rest time, short repeats, there is some evidence to suggest that would have a beneficial effect on VO2 peak, for example, but it's not as strong as the more kind of commonly held moderate physical activity kind of messages.

P5 Yes, and you can always visualise that more in adults than in children. The data from Toronto that we've talked about before in CF children, showing that CF children who do exercise more formally have a better respiratory function. Did they measure parameters of VO2 max and that type of thing, or was it just the physical activity monitoring?

P1 I don't know, sorry.

P5 I can't remember, but that would be something important.

P1 OK, so we've been chatting a little bit longer than expected. I don't know whether you feel that you've come to a point of consensus, or whether you want to talk a little bit more over ideas and opinions. So the main themes that sort of came out for me was like there's lifestyle

approach, reinforcement through family, parental encouragement, and also what came out for me was parents being facilitated, and the patterns of physical activity levels, and as such, reflect those seen those among mainstream non-CF young people and children. An area of concern seems to be the point of stoppage, and again, this seems to reflect mainstream children and young people in the teenage years are quite critical. That could be because of academia or academic pressures, such as GCSE year being a prominent issue, rebelling against parents, sort of the parental encouragement almost backfires, but there is also, I suppose, that, I won't say forgotten group, that's not fair, but that other group, sort of the opposite of those who get a lot of parental encouragement, those young people and children who have chaotic lives, and people coming in and out of their lives, how would physical activity monitoring help them? Experience of CF symptoms was discussed, and we spoke about what emerged from the results, the themes that emerged, the motivation, and issues of people who maybe feel demotivated. How can we help them with physical activity monitoring? And this sort of again pushes towards a more tailored approach. What can we do to reinforce those children and young people? Would we bring in other approaches to help them, such as counselling and motivational interviewing and that type of thing, looking at cardiac models as well? The type of tests that are done, that was brought up, so short tests at the moment. There was a discussion about an issue of it's more of an endurance test, or is it a fitness test, and the importance of field tests was particularly highlighted. How can that measure or reflect what happens in the children or young people's real life, real setting as such, and there's a concern about how children and young people assimilate the knowledge and the feedback that the devices give to them, how they can assimilate that into their own sort of experience of physical activity and exercise. Hang on. I'm just trying to make sense of my own notes now. Again, there's some adult evidence with regards to the hit approach, adult evidence with regard to fitness being an indicator of longterm outcomes such as quality of life and longevity, and how this correlates with physical activity, and there was a discussion about hit approach, and also there was some discussion about structured activity. You can go to a structured activity, but it doesn't necessarily mean that the young person will be active during those times to a level or intensity that'll have health-related benefits. And, I suppose tied in with that, there was a discussion about perceptions of normality, in terms of interpreting the guidelines, and that feeds into parental knowledge really, in this family model. There seems to be quite an issue, a more prominent issue, parental understanding of physical activity, and parental uptake and initiation of an active lifestyle, and they were the main themes that came out for me.

P3 You've done well there, P1.

P2 Hasn't she? Excellent.

P1 Is there anything anybody wanted to add to that? Detract, take away?

P5 So just to finally just focus on the differences between the cohort that we've looked at and children without CF, so what would you say those are? Obviously this perception of maybe not being quite as able as children without CF to do exercise. So that's one point, and also a more pressurised situation in that we, if you like, have been prescribing exercise all their life,

so that can lead to some tension, possibly with the parents and with themselves, as they grow older and don't do the exercise. But those are probably generic problems. But I don't think a child without CF would have quite so much pressure on them to do exercise.

P1 No. Within the children and young people that were interviewed, I don't feel that it was necessarily, exercise and physical activity wasn't deemed as a chore.

P3 Good.

P1 I didn't get that. I really didn't. It was just something quite natural.

P3 We try not to. Yes, we try and take the emphasis kind of off the prescribing aspect, you know. Certainly in the adult world, it will be prescribed, but that's not the way we bring it up here.

P1 I always enjoyed as well that emphasis was brought across as well, so it wasn't like, "Oh, I've got to go and jump on the trampoline for ten minutes". It was, "Ooh, you know, I can go", like one person skated in her Mum's kitchen on a Saturday afternoon because it was fun, and her Mum didn't care, because they were getting exercise, you know, engaging in an activity. So it wasn't deemed as anything negative. In fact, in many respects it was deemed as an opportunity to socialise, and socialise with other people where condition isn't an issue, which kind of brings in physical activity being a normative, I suppose, escape, really, and as well there was that competitive element, "I can do what they can do", but I think within that, there was a recognition that there is an underlying condition there.

P2 I think one thing that we might need to look at is almost this particular project, if you're then taking money out of the equation, which seems a bit random, but in terms of what we've done with a group, that process could then operate at an individual level, so in the same way that we've conducted interviews with parents and children, and then given them the choice of devices, and then consolidated that with a survey. It almost feels like there's a possible model there that could be supported with some behavioural counsel. We didn't take them as far as looking through the results. I know some of them were very inquisitive about the field. What does it mean? And some of the devices give you that instant feedback and some don't, but there's almost s model there to be able to take them through, in terms of the activity monitoring and this kind of wraparound support. So rather than it be exercise prescription, that there is some support around that to help them to know what the data means, and supporting the family and that kind of intervention. We've stopped short of that, but I know it's a formative study, but actually the structure of that, if that could be brought into some kind of model that could be operationalised with the patients that you've seen in a consultancy model, it might have some benefits, albeit a protracted. It doesn't need to be over weeks and months like it was here, but I guess the main facets of that process would seem to be useful in bringing that into clinical practice maybe. Maybe I have a Utopian view, but is there anything that you think would be reasonable and practicable to look at?

P5 The people who are looking at adherence in CF are, there's a trial going on at the moment in adults coming out of Sheffield, which is looking at analysing each individual patient, the underpinning issues that cause their non-adherence, and then providing a bespoke tool, whatever that is, to improve their adherence, and you could argue for a similar sort of thing

for our patients with regard to assessing their physical activity monitoring, looking at their life, their lifestyle, and then providing them with a bespoke, but you don't like prescription, but sort of advice and get them setting their own goals.

P2 Yes.

P1 I suppose there could be an argument that by involving the patient as well, that might actually bruise adherence.

P3 So by doing it in a donation theory point of view.

P1 And doing it in a supportive way, rather than a chastising way, for want of a better word.

P5 Or a dictatorial sort of way, so they come in, we say, "You've got to do this". We're just then becoming like the parents, aren't we? Yes.

P2 Well, certainly some of the barriers, when you look at the barriers, when you look at the whole data set, are around chaotic lifestyle, other things that are controlled that need to have that family intervention in order to generate an effect. So you're almost setting them up to fail without having those foundations in place that, I guess, if you take the clinical population out and look at what we do with families generally in the community, that has to be the model that we operate has to be this kind of buy-in, and it has to be a whole family approach, and we're just recruiting on a study at the moment looking at whole families, from a monitoring point of view, and that's quite interesting. So yes, I think that that's something that could be taken forward. There's lots of inspiration from other programmes, whether it be children with obesity. In interventions with the child with obesity you don't just, for want of a better phrase, treat the child, you work with the family, from a multi-disciplinary approach. So without wanting to make too many parallels, there are models out there that say, "Well, if you just work with the child", we know if you just work with the child in school, and not look at the other...So maybe looking at the family, because it's often, from the data, parents can be barriers to this perception of normality. So the parents have a different perception of normality as to what a normal child at that age would do activity-wise, but also in terms of general physical activity themselves. So maybe re-addressing those perceptions may help the child's perceptions of low competence, when actually, they could be by virtue, normal.

P1 So I'm conscious that we've been chatting a little while, but we haven't really talked about the clinical aspects, and the results that emerged from the survey. So I don't know whether you fancy a five minute break. I'll list the ones that are more...and maybe....

P2 ...Counselling, testing and monitoring, in terms of trying to consolidate the point that there needs to be some education for practitioners, parents, children, and more or less like wraparound counselling around the physical activity, which as we know, comes at a cost, and then these notions, and it's not my area of expertise, but it's around testing, and what needs to be tested, that's relevant for lifestyle, as you said, P3, and then what we can get from the actual monitoring, and how valuable that's going to be, to look at discreet

opportunities for... The testing and the monitoring will provide us with information about intervention.

P4 Where do you see the distinction between testing and monitoring?

P2 So testing, as I see it. This isn't my area of expertise, but the testing would be like the VO2 max, then tests that go on here when the patients arrive for clinic. So the tests...

P3 The once a year...?

P2 Yes, so it's a one off.

P4 Could you not have that monitoring as an umbrella term, so the monitorings could be an ongoing thing in terms of the behaviours, and part of that is also monitoring the kind of products of that behaviour, which might be the exercise-type testing?

P2 OK.

P4 I just think, going back to the...Well, a few weeks ago there was a report called Generation Inactive, and so this body called UK active making what's lobbying calls, fitness testing with kids in schools, and it either pushes you that way or that way, really. But if you actually start to think about what monitoring evaluation of all that...

P5 Sorry, it pushes you...

P4 Well, it pushes you to, "Oh no, I'm dead against that", or "That's a great idea".

P5 Right, so it's quite...

P4 And it's quite a divisive kind of subject, unless you start to unpick really what it's about. It should be about monitoring and feedback and education, all those things. You could have in the banner of that is something around monitoring, or a less harsh term is testing. Testing infers pass or fail almost, and I'm just thinking about the language maybe, that we might want to use some of these things INAUDIBLE (tape 2, 02:32). Does that make sense?

P2 Yes.

P5 So a physical activity support package, as opposed to monitoring and testing.

P4 Yes.

P5 Activity facilitator. I don't know.

P4 Yes. The language is more supportive, rather than just mental...

P5 I mean, the companies that are doing it, Apple and Nike and so forth, they seem to be doing it in a very sort of, I don't know what the word is, but they seem to make it look a really lovely thing to do, marketing and so forth. So we could learn from them.

P2 I get the principle behind that is the instant feedback, isn't it?

P5 Yes.

P2 It's about those individuals who are looking at, they're not getting somebody who then helps them to say, "Well, ok..."

P4 You might get that instant feedback, but you don't know how to interpret that information correctly. That could have a negative impact on your motivation, or a positive impact, or no impact.

P2 Yes.

P4 It could just be another set of numbers you're given, and it's about having a supportive network to enable you to understand what that actually means.

P2

In terms of the education, I think that kind of counselling, it doesn't have to be a psychologist. It can be, that type of support can be offered in different ways through different roles, through training, through educating parents. It's not necessarily about parachuting a specific practitioner in. I guess it's looking for that opportunity, capacity, and the willingness to sort of take on board some of that, in the same way that exercise professionals who work in gyms, they're not exercise psychologists, but they are very much at the front end of applying principles of exercise psychology in order to make sure that the people they address, whether it's the cardiac rehab plan or reducing obesity, whatever it is, they're sort of applying the technique. So it doesn't necessarily mean another specialist individual into that kind of arrangement that you have as a clinic maybe.

P5 So what does the evidence, so that's one-on-one motivational coaching, but it's their evidence in your theory that just monitoring activity makes people do more activity.

P4 No, probably not strong enough to say that. I think...

P5 Sorry to interrupt, but you've got to motivate. If you've got somebody who wants to get fitter, then the physical activity monitoring will help them to do that.

P4 It will be positive, it'll be stronger, if there's information which will allow them to know how to do it, where to do it, who to do it with, so the information...

P5 It's a facilitator.

P4 Yes. Information beyond the, "You've done x number of minutes. There's your goal for next week". It's about, "Well, how do I do them? How is that facilitated? What are the mechanisms for me to go from there to there?"

P5 So what we're worried about is, and I know your data don't suggest this, because they all seem to have done a lot, but we're worried that our patients are not motivated, this is another burden to them, so is just giving them this going to make them motivated or just make them miserable? I don't know the answer to that question.

P1 I'd think, from what little, because obviously it was quite a small cohort, wasn't it, quite a small sample, from the information that was given and with regards to this place, I think it was sort of things like the Actigraphs and the accelerometers, they were received quite blandly, but they do look quite bland as well to the young people, and obviously the young people are engaged in the project, and wanted to know what level of activity they were doing, but they weren't able to get that information until after they'd worn it quite a long time.

P4 And that's the distinction between a research tool, because we don't necessarily want them to know how active they are, because then that will change their behaviour, against the wearable, consumer-led tool which has that at the forefront.

P5 And which is instant feedback gratification.

P4 Yes. But what's happening now is, there are more studies which are looking at the ability of those consumer tools to see how accurate they are, standing up against the research grade kind of...

P2 Because that is a push.

P4 Well, it is and it isn't in a sense, isn't it? I mean, if you've got maybe consistency of the tool or the time, so you know that is an increase or a decrease rather than an absolute level, maybe that's, from a motivational perspective, maybe that's more important than, because as I've shown on the other slide there, even with the research kit that we use, in the literature there's still lots of disagreement about how best to then interpret that information, and apply it in terms of public health messages.

P1 And I suppose one key thing to consider is, ok, like a commercial tool that gives feedback, but at what point does that feedback almost reach a saturation point where anybody'll look at it and go, "Oh, I've done ten thousand steps". When does that then stop becoming a motivational goal-set tool?

P4 Plus, I guess, there's risk there with the children in this study is that the Fitbit stuff, because it is very attractive, that the novelty of it might have been very positive for them initially, but do we know that would be sustained over time? And also there's those things which we've not been able to tap into which we know are risks the longer you put interventions in place.

P3 Can I add how useful it would be as a clinical team to have data on seven days wear time that you've got. Would that be something that would be relevant...

P2 Yes, because...

P5 Oh, it would be massively interesting. Whether it would do our patients any good is a different....

P2 Yes. It's whether you'll get them to do that. Or you might get...

P5 It would help us to identify problems, wouldn't it?

P2 Patterns and...Yes.

P3 And opportunities for intervention.

P4 There's two research devices here. One was this black box on the hip. One was a black box worn on the wrist. So some of the kit we've got now is a wrist-worn device which looks like a watch, tells the time, it'll tell you steps as well, but it also will do all the research grade stuff as well. So I guess since we did the data collection, the field has progressed a little bit to the point where they've got a bit of a halfway house, because some of them are quite attractive to use, and someone doesn't go, "What the hell's that on your wrist?" Well, it's a watch obviously, and there's time, but it'll still garner the information for the researchers, so typically what we sometimes give to schools and kids is, "OK, this is a snapshot day, this is 6 am, this is midnight, this is a trace of activity, this is moderate, this is light. What were you doing here?" And it is a connotation prompt. "This was in the morning, or this was playtime", and if nothing else, sometimes that's useful information. I could think of it in clinic for you guys is to set as a prompt, "Talk us through your day here", and you'd be surprised where these peaks and troughs actually occur.

P5 Is it measuring their heart rates as well?

P4 The kit we're using at the moment isn't. I mean, there's variations on this stuff all the time. So these are accelerometry-based, so they will measure accelerations. Yes, I mean, lots of different bits of kit out there obviously.

P1 I think a benefit to that type of kit as well would be it removes, as well as the feedback it gives to the young people, but it removes that stigma about, "Oh, can you tell me the time? Look at the state of your watch. It looks like an old man's watch almost".

P4 And they do actually look all right.

P2 We're finding that. So we're running some compliance studies with two age groups at the moment, some quite large sample sizes, around how we can increase compliance. So again, informative. So we're asking them what would make them comply, and then we're running the studies at the moment, and there are incentives. We incentivise as one of the, but there's also things like text reminders, immediate feedback, social conformity is a big one.

P5 This is all round exercise?

P2 Yes, this is around the device wearing. So one of the problems that we have is that we don't get incomplete data sets. So if you wanted a full seven day, say, it's important, in a similar way that we've done here, to ask children what's going to...We need to understand what will it take for you to be able to wear that? And not everything is about giving them twenty pounds worth of Amazon vouchers or whatever it is. Sometimes it is around reminders to put it on, take it off, in your diary, and social conformity. But then the other one that's coming out quite strongly is this notion of "We want to know what the data says. We want to know what the feedback is from the devices as well". So it isn't always money-driven.

There can be things that can be employed that can increase the chances of compliance, from what we're finding out with other studies with children and young people, so...

P5 And how successful has that been, that sort of approach?

P2 We haven't got the data back at the moment, yes, but we did a similar formative approach, and we asked them what would make you comply, and now we're running comparison groups with different incentives across two age groups, so the younger primary school and secondary school. There are variations, as you would expect, in terms of what incentives they want across two age groups. But I guess the data that you receive to be able to work with needs to be seven days full wear time in the same way that...It's frustrating when you get data back and it's not complete as well. That would only be of value to you in the same way that it would be from a research point of view.

P1 So I'm just thinking, I'm conscious of time as well. What do you feel the clinical barriers'd be to these devices?

P5 Cost principally, and...

P3 It's actually looking at the information. That's a time issue and resource, isn't it? Who's going to look at it?

P5 But it would involve the families, and they are able to download the data, and you're sort of sharing that responsibility. Yes, but that was the vision, wasn't it?

P3 Yes.

P5 We're very keen to sort of have this where it's more of a partnership thing, rather than us watching them. They're watching themselves, and we're chipping in and giving them support.

P1 Would trust be an issue?

P5 Trust?

P1 In the parents.

P5 Well, we haven't . P3's done a lot of monitoring with electronic data capture with a nebuliser device, and we've always done that in a very open way.

P3 Yes. And actually, as you said before, they want to see the data. They want to see, you know, they'll come back and say, "Show me what I did", and the perception of what they did is very different, in a positive way sometimes, not just negative. But they do, they want to see. So I don't think...

P5 And you're able to adjust things to help them.

P3 Yes.

P5 So again, like a bespoke thing, you're going, "Well, look..."

P3 That's right. It's tailored then.

P5 "You've not done one morning's nebuliser for the whole week. Let's get rid of that and focus on..."Then actually, when you do that they improve at doing the other types, so it's quite interesting.

P1 What would you identify to be the clinical facilitators of the devices? In that respect, and in terms of importance, who, where the responsibility lies? I'm looking at you.

P5 It's interesting that they perceive the physiotherapists as being the key, so with regard to exercise, because it should come from the whole of the team, shouldn't it? It should come from the dietician, the nurses. It should be like a team message coming across, but I guess...

P3 Well, I'm sure there is as well, but...

P2 Yes, they were asked about who, they were asked about an individual.

P3 Because we do the testing, so I think they will see, plus every clinic they see us. One of the questions is, "Tell me about what you've done the last couple of weeks, or since last clinic have you taken on a new sport?" That kind of...So I think they do definitely, because we do the most discussion about it.

P2 So it's because you're established in that role already, so it's...

P3 Yes. Either A or I, whoever's in clinic, we'll do the same.

P2 Almost pointless to try and create in somebody new, because the trust won't be there.

P3 Yes.

P1 Is there any other issues that anybody wants to discuss with regards to the healthcare professional findings, and where the monitoring devices fit in, in terms of clinical facilitative barriers, and the importance of the monitoring in terms of clinical perspective. OK, so what I got from the discussion then, again just to recap...

P2 Sorry, P1. Maybe we should just parallel that with research outcomes.

P1 Oh yes, of course, yes.

P2 Just in terms of what we feel are the sort of priorities are in terms of research, or facilitators will agree with the cost, I think would be...We need some funding to be able to do this.

P4 Cost and compliance work across all, both users, and I guess the biggest difference is the type of data that is produced, the research outcomes as opposed to the users, and I hope we can...I don't know whether it's appropriate for the same tools to be used for both kind of outcomes, because I think they're designed for different things, aren't they, in a lot of ways, and this one Actigraph would go some way towards some of this stuff. I'm sure as this field moves on it will all merge into one, I think, in terms of usability.

P5 I can't visualise an outcome that you would be interested in from a research perspective that patients wouldn't be interested in, because that would suggest to me that the research outcome isn't very important.

P4 Well, the data side from a wearable, that the user will see on, say, a Fitbit interface, would be so reduced down that in terms of from a research perspective, you'd need really to unpick that data to make sense of it, analyse it and interpret it in a more in-depth way.

P5 That's different to them not being interested. They'd still be interested, but what you're saying is it's too complicated to assimilate...

P4 Yes. There's a degree of, if you like, technical expertise required to deal with that data, interpret it and then apply it back again. I mean, ultimately I think we're about applying what we find to practice, and to the user. But I think at the minute the tools that are out there are designed for different groups for different purposes, although they're all telling you about physical activity.

P2 So in terms of research evidence, it would need to be the research grade devices that would be used to inform?

P4 Yes. Unless there's stronger evidence that the wear of the consumer tools actually stand up well enough against the established research tools.

P5 And so a validation of ....

P4 Yes. The Fitbits and what have you, yes. But those things are happening. Different groups are doing those already, so you find watch this space almost for that kind of thing.

P1 I suppose what came out for us was the cross-contamination issue, which didn't pose too much of an issue for us, but from a research perspective it may be an issue if you have a massive amount of children using a limited number of devices. We wouldn't want anybody to get poorly as a result.

P5 Cross-infection.

P1 Cross-infection, yes.

P5 Yes, we wouldn't. For this to become clinically available, we would need to have single use.

P2 Would it?

P5 Oh yes, definitely, yes. I think we can get away with it as a research tool, but not as a clinical tool, not any more. Everything that we have now is single person use. Everything we use in theatre, everything.

P1 So I suppose that ties into the costs as well, doesn't it?

P5 Yes.

P2 And I think, looking at some of the ages of the children and their conversations in the interviews and things, they are very articulate and able to talk about these devices, and therefore in any future research we should make sure that it's not just research on children, it's research with, and alongside, and involve them in the design in terms of...When we went to the young person's forum, and they were great, and we made some changes to what we did based on that, and it'd be great to work...

P5 You should include that in the paper.

P2 Yes, we are, yes. But we did make some changes, and we fed that back to the group as to what changes were made, and I think this type of research is really important to get the children and young people's voice all the way through, and that's what would make it different, I feel, than just a research project, you know, research on the children. Because certainly, their level of literacy and understanding of their condition comes through, but also comparatively, they were very, very insight led. I've done interviews with lots of children and young people, and the level of detail and the conversation was very, very good, and that may be as a consequence of the way that they engaged generally, talking about their conditions anyway, but it provides us with that contextual insight. When we ask them, "So what were you doing at twenty past two on a Friday afternoon?", they're able to articulate that, because some of the interviews were quite lengthy really, weren't they?

P1 Yes.

P2 Which is great. It wasn't restrictive in any way, so they're quite unique in that sense.

P5 I think that probably merits highlighting as well.

P2 OK, yes.

P5 Because we've talked about what the differences are between this cohort we're looking at and children without CF. I think that's really important, they are pretty institutionalised. They're quite confident with adults because they meet adults, they meet the CF team on a regular basis. We will talk to them as individuals right from a very early age, and that's not normal really. You don't get, apart from talking to your teachers, children don't get much exposure to adults.

P2 Well, no, and we tend to work with children about activity interventions in focus groups because it's easier to get them to talk in groups. These were individual interviews, and the level of detail and depth around some of the answers was great, so that really is a strength, and something to capitalise on in future research.

P3 Another difference was that breathlessness aspect too, the perception that it was bad with the CF, whereas that might be very normal if you talk to youngsters without CF.

P4 It might just be a reflection of general low fixed levels of...

P5 It shouldn't be, really.

P4 Well, it depends what we were doing, I guess.

P5 But are you saying more their perception that breathlessness...

P3 Is bad, and is part of the CF, so therefore, I think it was number six. Four. Unpleasant symptoms. Breathlessness hinders capability.

P5 So actually there that's quite a big culture shift. You need to be telling them that you need to be breathless from time to time.

P3 Everybody's out of breath.

P5 Everybody gets breathless.

P3 That's what we say to them when we do the exercise test. We are going to make you breathless, and that's the purpose of it. That's how they...

P5 And that's normal.

P3 And actually, you can stop a parent saying, "Stop!" Because they're seeing their child breathless, where we don't want them, we want to see how they cope with that, so...

P2 But parents of children at a normal activity whatever, multi-sports thing, would be saying the same. "Oh, he's looking a bit pink in the face. He's sweating a bit there. Perhaps you need to sit down and have a rest". That's typical parenting behaviour from somebody who's not got CF, and that's because of the way the parent understands the effects of physical activity, that that's ok, that's a normal healthy response, but they see it as being, "Oh, I don't want to see them sweating", and it's how they interpret it, so there could be some education around that as well.

P1 OK, so anybody got any more to add?

P5 Well, I think we could talk for ages, but I'm afraid I'm going to have to go, though. It's been very good.

P1 Yes. Brilliant.

P5 Where do we go from here?

P1 So could I just recap quickly the points that are raised, just to make sure I've not missed anything? So education from practitioner, parent, and also children and young person was highlighted, and it's important to look at the language used, testing versus monitoring, and make the distinction between the two. For example, looking at physical activity support package rather than testing, because that could be inferred as being judgemental. Feedback has again been highlighted as important, and supportive network, and again, looking at the principles, applying the principles to ensure that people progress, and looking at different ways that the feedback is offered, and also the roles of different people that may deliver that information doesn't necessarily have to be psychologist. It could be the physiotherapist, it could be somebody else. Also looking at the devices themselves, research tools versus

commercial tools. Some of the issues regarding research tools with regards to they are reactive, you don't necessarily want that feedback, because then people will react to that and sort of spike the physical activity levels, but then there's the question about the validity of commercial tools. The sustainability of the tool in terms of avoiding reaching a ceiling with regards to young people and children's physical activity. That's a consideration. Also, from a clinical perspective, the importance of seven days' worth of data. There was a few issues highlighted there. It's an ideal opportunity to identify any patterns of physical activity, and also identify opportunities for intervention. Issues with compliance was raised, the difference that it may make to different age groups. Some compliance again, the types of prompts that are use to boost compliance, and also clinical barriers were highlighted, costs, time, resources, working in partnership with parents. It's not perceived to be just a clinical tool. It's perceived to be, again, engaging the family, and tailoring advice and information given, and although the team message was highlighted as being important with regards to who's best placed to facilitate the physical activity monitoring, physiotherapist seems to be quite well placed for this role. And again, with regards to cost, it has to be highlighted it would have to be single-use devices only. So again, that has a cost implication. Research points of interest kind of mirror quite a lot of the clinical ones, so cost and funding, compliance, in terms of use, the type of data that's produced, so research outcome versus user-friendly. Is it appropriate to use the same tools for research versus the type of role or the purpose for having monitoring. Is that the same as research needs? Involving children and young people in the research process, that was highlighted as very important, making sure that the young person's voice... but that crosses again very much into a clinical perspective also, because you always take that position anyway. Children and young people engage from the get go in their own health and their own health outcomes. Yes, and just capitalise on that really, in terms of research. That was about it. That was what I got. Is there anything else anybody wanted to add? No? OK. Thank you.

End of Meeting
