# Supplementary material for: A formative study exploring perceptions of physical activity and physical activity monitoring among children and young people with cystic fibrosis and health care professionals
Source: BMC Pediatr. 2018 Oct 23;18:335. doi: 10.1186/s12887-018-1301-x (PMC6198445; doi:10.1186/s12887-018-1301-x)
Supplement: Supplementary file 5 — Phase 4 - Expert Panel Working Document.pdf. (PDF 307 kb) [file 12887_2018_1301_MOESM5_ESM.pdf]

Phase 4: Expert Panel Meeting

A Formative Study Exploring the Use of Technology-based Strategies to Reduce Sedentary Behaviour and to Promote Physical Activity among Children and Young People with Cystic Fibrosis.

`

Introduction
Thank you for agreeing to take in the expert panel meeting. Please take a moment to read the enclosed information below which provides some details about what the meeting will involve, and also some guidance on how to prepare for the meeting.

Purpose: To consider the evidence collected during Phase 1 through to Phase 3 of the BUPA seedcom grant funded LJMU/Alder Hey Cystic Fibrosis study. The aim of study is to determine the acceptability, feasibility and effectiveness of physical activity monitoring devices. Using an adapted consensus technique approaches, informed by the Delphi and nominal group consensus techniques common to both the medical and allied health professions, expert panel members are invited to examine the findings of each phase (presented below) and to consider the wider implications of physical activity monitoring among children and young people with CF.

Attendees: Five panel members in total will be present; namely a CF clinical lead consultant, CF specialist physiotherapist, a dietician, a physical activity expert and a qualitative research expert. In addition, a research assistant will be present to assist the qualitative research expert in facilitating the meeting.

Pre-Meeting Considerations: Please fully read this document. As an expert panel member, you are asked to consider the study findings, and to formulate your own opinions and ideas concerning the key issues surrounding physical activity monitoring in children and young people with CF. Please feel welcome to make notes, as you will have an opportunity informally present your perspective and ideas to other panellists at the meeting.

Meeting Structure: The meeting will be audio recorded and be structured as follows;

1. Having considered the results of the study prior to convening, each panel member will have a maximum of 3 minutes to informally present their ideas from their chairs that they have independently formulated to other panel members. Please note that other panel members will have read this document ahead of the meeting, therefore will also be familiar with the study findings to date.

2. Panel members will be invited to discuss the ideas presented as a group. During this facilitated discussion, panel members will have the opportunity to clarify and elaborate upon their ideas which they have presented. A maximum of 25 minutes will be allowed for this task.

3. Next, each panellist will be invited to spend the next 10 minutes independently ranking the importance of the ideas presented by the expert group.

4. The meeting will adjourn for 15 minutes. Following this short break, the facilitators will present a summary of the ranked ideas to the panel.

5. Panel members will be invited to discuss the ranking of the ideas and issues raised paying particular attention to those that are ambiguous or with which they may disagree, for the next 20 minutes.

6. Following this discussion, panel members will be asked to rank the ideas presented independently in order of perceived importance.

7. Lastly, the facilitator will summarise and present the results to the expert group.

Diagram 1: Flow Diagram Representing Phases 1 to Phase 3 of Study

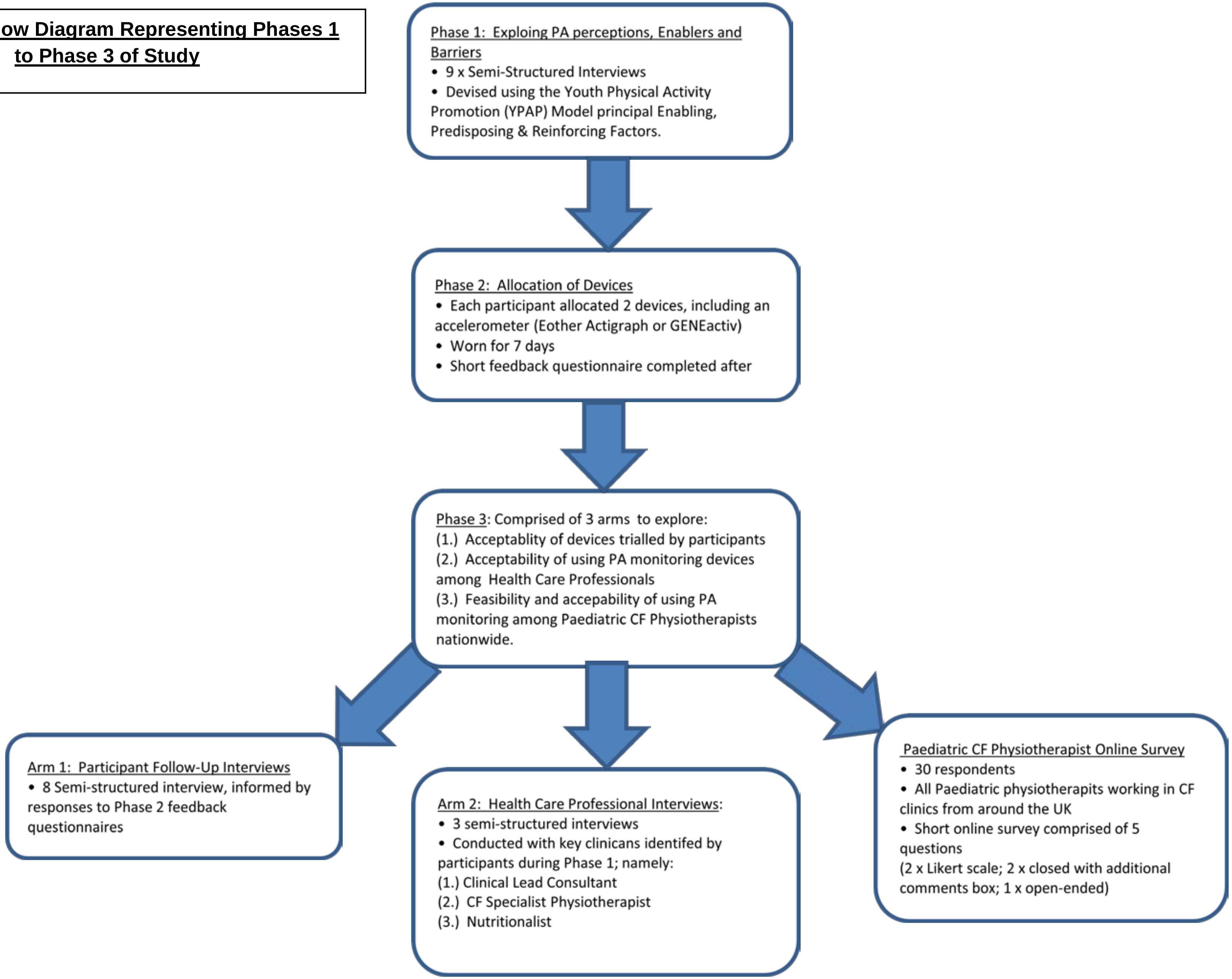

Study Findings

Phase 1 (n = 9): Key Findings
1. Most participants self-report as being high active, engaging in a diverse range of physical activities (e.g. football, table tennis and Thai boxing) and regularly engage in community-based physical activity facilitated by clubs/leisure centres.

2. Participants are aware of the physical activity facilities available in their local community.

3. Competing physical activity commitments and priorities pose a challenge for those wishing to try out new activities as highlighted by one participant;

> "*One of my mates has started going to a running cubs, and she's been saying that its good, but I do dance on the days that she goes, but if I didn't do them, and I didn't really do much, I'd probably have go and have a go at it." (Phase 1, P7, pg 10, lines 438-440).*

4. CF is not perceived as a barrier to physical activity per se, rather, unpleasant symptoms (e.g. breathlessness) exacerbated during physical activity hinders capability.

5. Poor physical activity performance was attributed by participants to the deleterious effects of CF symptoms.

6. Participants perceive themselves as less able to achieve the same level of physical activity attainment as their non-CF peers.

7. Perceptions of individuals who are active and inactive are consistent with related stereotypes.

8. Maintaining health and well-being, and improving fitness to mitigate unpleasant CF symptoms emerged as a key facilitator of physical activity.

9. Participation in physical activity provides participants an opportunity to socialise with friends and promotes a sense of peer cohesiveness.

10. Congruence between parental and participant PA-related beliefs was reported.

11. Physiotherapist's were predominantly identified as the most influential member of the CF team and PA- elated advice was regarded as trustworthy.

Phase 2 (n = 7): Key Findings
Accelerometer data revealed that, in the main, participants involved in the study exceeded the recommended 60 minutes of daily moderate-to-vigorous physical activity (MVPA) (M = 192 minutes per day). Notably, MVPA thresholds are monitor and protocol-specific. To date, there is no agreement within the field as to which thresholds most accurately reflect actual MVPA.

Phase 3: Key Findings
Participant Interviews (n = 8)

Overall, participants reported that they enjoyed wearing the physical activity monitoring devices that they were allocated to.

1. Pedometer – (P1; P5)
- Overall, this device was perceived as being a mediocre physical activity monitoring device by those who trialled it.
- Participants enjoyed being able to see how many steps they had achieved throughout the day on the visible step counter, which was perceived as being useful for goal setting, particularly among other CF youth.

- Whilst the pedometer did not interfere with participant’s ability to engage in physical activity, it would sometimes fall off during activity. For example, one participant reported on the strategy they used to prevent the pedometer from falling off during Football;

> *“...sometimes it'd fall off, so when I went to footie training I'd put it on my shorts, but then I'd put my underwear on there over it, and I'd tuck that part of my tee-shirt in so it wouldn't fall off…” (Phase 3, Pt 1 ,pg 2, lines 41-43).*

2. Actigraph (Waist-worn accelerometer) – (P1; P2; P5)

- Participants who trialled this device found the waist-worn elastic belt could become uncomfortable (e.g. digging in at the waist). Also, the elastic belt often became loosened throughout the day and required participants to readjust it to prevent it from “slipping down”. The inability of this device to instantly feedback to the wearer their physical activity levels meant that it was not received as well as other more interactive devices that displayed how active the wearer had been throughout the day.

3. GENEactiv (Wrist-worn accelerometer) – (P4; P6; P7; P8; P9)

- Whilst easy to wear, this device was widely perceived as being cumbersome and “clunky”, making it uncomfortable to wear.
- Unable to conceal the device, this device often attracted unwelcome attention from peers who often assumed it was a watch and asked participants the time. Similarly, some participants were asked to remove the device to avoid hurting either themselves or others when being active (e.g. PE lessons).
- The lack of feedback provided by this device meant that participants felt they were unable to self-monitor their physical activity levels or physical activity related set goals. Indeed, one participant described the device as being synonymous to a “blank piece of paper”.

4. Fitbit – (P2; P4; P7; P8; P9)

- Participants reported that they enjoyed the interactive nature of this device. Being able to monitor their physical activity using both the device itself (e.g. double tapping the device to monitor step target) and by using the interactive dashboard (e.g. number of calories burned, steps etc.) was positively received. It was felt the feedback provided by this device encouraged physical activity related goal setting and may promote and encourage sustained physical activity amongst others.
- This device was perceived as being comfortable to wear and did not attract unwanted attention from peers. Moreover, this device did not interfere with activity, however since it is not waterproof, participants were unable to wear it whilst bathing and when participating in water sports.
- However, its reliance upon being synced to a computer to access the dashboard facility posed some access difficulties for some participants as most relied upon use of their parents computer/laptop which restricted their ability to monitor their physical activity using the dashboard.

5. Moves app (P6)

- In facilitating physical activity monitoring, this device prompted self-monitoring of physical activity and was perceived to promote physical activity engagement.
- However this physical activity monitoring app requires the user to carry their mobile phone on their person to capture their activity levels. Whilst the participant who trialled this device reported that they typically carry their phone with them everywhere, they were unable to take their phone with them when they were swimming or playing football.
- The participant continues to utilise this app to monitor their physical activity.

Health Professional Interviews (n = 3)

- Current physical activity monitoring is limited to saturation monitoring, a modified shuttle test and self-reported physical activity. These approaches are perceived as being useful for understanding how CF youth cope with and how much physical activity they engage in.
- Whilst these measures provide a generalised indication of health and promote a discussion around physical activity among CF youth, the crudeness of the measure does not impact directly on the management of patient care and only facilitates generalised advice as opposed to individualised advice being delivered.
- Moreover, current assessment techniques are inadequate for those patients who are able to complete them.

- The use of technology-based physical activity monitoring was perceived as being potentially useful for establishing an understanding of patients' physical activity patterns, enabling them to be responsive to any subsequent changes that may occur.
- They may also enable clinicians to deliver tailored treatment to suit individual patient needs and to determine activity levels beyond the clinical environment.
- The CF specialist physiotherapists were perceived as being best positioned to assume responsibility for implementation and management of physical activity monitoring devices.

- Reported barriers to using physical activity monitoring include cross-contamination, cost-effectiveness of the devices, and resources including staff.
- The use of physical activity monitoring devices hinges on both the acceptability of the devices among CF youth and the reliability, type and usefulness of the data collated.
- Whilst physical activity monitoring feedback was perceived as a potential mechanism for motivating some CF youth and encouraging positive health-related choices, it was acknowledged that negative feedback may discourage inactive patients further.

Physiotherapist Survey (n = 30)

- Out of a total of 30 respondents, 26 report that they currently assess physical activity.
- A wide range of assessment techniques were reported including a modified shuttle test, 3 minute step test and cycle ergometry.
- Physical activity information is used in a variety of ways including as an outcome measure to evaluate treatment efficacy, to evidence patient exercise tolerance, to guide interventions and to deliver physical activity related education.
- The majority of respondents (n = 28) felt that physical activity monitoring could influence clinical practice and objective measures could provide more accurate assessment of patient physical activity tolerance, facilitating a more tailored approach to clinical care in the delivery of individualised advice and interventions given to their CF patients.
- Compliance was reported to be a perceived to be a potential barrier to physical activity monitoring implementation.
