# Supplementary material for: A formative study exploring perceptions of physical activity and physical activity monitoring among children and young people with cystic fibrosis and health care professionals
Source: BMC Pediatr. 2018 Oct 23;18:335. doi: 10.1186/s12887-018-1301-x (PMC6198445; doi:10.1186/s12887-018-1301-x)
Supplement: Supplementary file 6 — Phase 4 Delphi process.pdf. (PDF 57 kb) [file 12887_2018_1301_MOESM6_ESM.pdf]

Step 1 of Delphi-Nominal Hybrid Technique: Evidence Analysis

Step 1: Evidence Analysis
Expert members will be asked to review of key articles relevant current empirical research & methodological perspective of current study; Expert members will be asked to review of key articles relevant current empirical research & methodological perspective of current study;
(1.) Hulzebos et al., (2014): Measurement of Physical Activity in Patients with Cystic Fibrosis
Step 1: Evidence Analysis
Expert members will be asked to review of key

Step 1: Evidence Analysis
Presentation of key findings that emerged from earlier Phases of the project;
(1.) Phase 1: Children and young people perceptions of physical activity.
(2.) Phase 3: Children and young people's experience and opinions towards PA monitoring devices. Also, CF team clinicans perspective on the feasibility of monitoring approaches tested, further supported by the results a nationwide survey of physiotherapists.

Step 2 of Delphi-Nominal Hybrid Technique: Preparation of Research Relevant Questions and/or Statements

Step 2: Identification of key research priorities:
Drawing on their respective expertise, panel members will be asked to consider and synthesise the evidence presented at Step 1 in the preparation of two or three research-relevant questions and/or statements relating to the key priorities associated with the feasibility of using technology-based physical activity monitoring devices among children and young people with CF.

Step 3 of Delphi-Nominal Hyrbid Technique: Delphi-Based Ranking Exercise

Step 3: Ranking Exercise: Expert members will be asked to rank and answer the developed research-relevant questions and/or clinical statements in order of future research priorities. Areas of consensus and non-consensus would discussed and preliminary recommendations for future research suggested.

Step 4 of Delphi-Nominal Hybrid Technique: Nominal-Based Group Meeting

Step 4: Nominal-Based Group Meeting: Following a nominal format, panel experts will discuss the preliminary recommendations developed during Step 3. Areas of consensus and non-consensus will be discussed and recommendations for future research would be proposed. In the interest of transparency and the sustainability of future research within the field, the purpose of each key research priority identified will be explicitly stated. Similarly, equal attention will be paid to divergent opinion.

Step 5 of Delphi-Nominal Hybrid Technique: Formalisation of Future Research Recommendations

Step 5: Future Research Recommendations: Drawing upon Step 4 findings, Project researchers will formalise and prioritise research recommendations identified. In the interest of transparency and sustainability of future research within the field the purpose of each research priority will be explicitly stated. Similarly, equal attention will be paid to the quality of evidence underpinning the ranking of key research priorities identified.

Step 6 of Delphi-Nominal Hybrid Technique: Review & Revision

Step 6: Review & Revision: Finalised recommendations will be sent to each expert member to review independently. Suggested changes will be taken under consideration by the project researchers. Amendments made will be informed by the strength of evidence provided by the expert.
