# Supplementary material for: A formative study exploring perceptions of physical activity and physical activity monitoring among children and young people with cystic fibrosis and health care professionals
Source: BMC Pediatr. 2018 Oct 23;18:335. doi: 10.1186/s12887-018-1301-x (PMC6198445; doi:10.1186/s12887-018-1301-x)
Supplement: Supplementary file 7 — Phase 4 Ideas presented at expert meeting.pdf. (PDF 204 kb) [file 12887_2018_1301_MOESM7_ESM.pdf]

Expert-Panel Meeting
(HCP-C)

Part 1: Patient-Related Issues

1. **Lifestyle based approach to physical activity**
   i. Parents as facilitators, including parental encouragement and the effects of direct and indirect positive reinforcement (e.g. being a role model by being involved in physical activity themselves) thus promoting physical activity engagement as a “normal part of life”.
   ii. A lot of what is reported in terms of their perceptions of physical activity by the CF children and young people is seen in “mainstream interventions” among the non-CF population.

2. **Decline of Physical activity**
   i. Teenage years seen as a pivotal point for onset of physical activity decline or adoption.
   ii. Pressures of academia, including time pressures, perceived as a barrier to physical activity.
   iii. Patients rebelling against parents, such that their encouragement for physical activity “backfires”.
   iv. Chaotic lives of some children can influence their physical activity.

3. **Experience of CF symptoms during physical activity**
   i. Children and young people perceived as being as capable as regards physical activity to that of non-CF children and young people.
   ii. Experience of symptoms should not be interpreted as a reason to stop or reduce physical activity.

4. **Importance of clinical versus “field” testing of physical activity levels**
   i. Importance of shuttle test highlighted.
   ii. Importance of “field” tests highlighted to provide an idea of patient capabilities in a more realistic setting.

5. **Motivation is a key issue**
   i. Importance of goal setting related to physical activity.
   ii. Relationship between feedback from monitoring devices and subsequent impact on motivation highlighted
   iii. The role of monitoring device feedback, and its potential negative effects, for those not motivated to engage in physical activity.

6. **Perceived importance of physical activity and the data retrieved by the devices**
   i. The perceived value of physical activity may be an important determinant of whether or not they will engage in physical activity monitoring.
   ii. Whilst CF children are exposed to information concerning their condition from a very early age, the question of how CF children and young people will make sense of/interpret physical activity related data retrieved from the devices and assimilate this information into their everyday lives was raised.

7. **Importance of fitness over physical activity**
   i. Evidence from adult-based research emerging to suggest that fitness is related to improved outcomes among CF patients
   ii. Does fitness correlate with physical activity level in children and young people with CF?
   iii. HIT (High Intensity Training) approach – does this translate for children/young people with CF?

8. **Structured vs. non-structured activity**
   i. Perceptions of what "constitutes" normal activity/normal intensity physical activity in this clinical population.
   ii. Evidence from the mainstream population highlights the importance of parents understanding the physical activity guidelines, and being aware that physical activity is not just a case of engaging in structured or organised exercise, but can be accrued through discreet periods during the day (e.g. playtime, walking to school).
   iii. Similarly, a patient may engage in organised physical activity (e.g. football training), however they may only be active for a short period of time within that coached session as opposed to those who cycle to their friends house or play out with their friends.

Part 2: Clinical Practice Issues

1. **Role of feedback provided by devices**
    i. Analysis of data will highlight issues of non-adherence.
    ii. Provide a means of conceptualising physical activity (e.g. you've completed X steps, this compares nationally for mainstream children like this).
    iii. Provides a means of "prescribing" physical activity at a bespoke level.

2. **Education**
    i. For the practitioner
    ii. For the parent
    iii. For the children and young people.

3. **Testing vs. Monitoring**
    i. Distinction between the two is needed.
    ii. The importance of language used;
        1. For example, implementing a physical activity support package as opposed to testing, as the latter could be inferred as judgemental (e.g. patients may infer that they either "pass" or "fail" when their physical activity is monitored).

4. **Importance of meaningful feedback**
    i. It is important that clinicians and patients are able to interpret the feedback delivered by the physical activity devices used.
    ii. A supportive network could be in place to facilitate the effective delivery of the feedback to patients
        1. For example, it does not have to be a clinician, it could be offered by different people in different roles (e.g. counsellors).

5. **Distinction between the use of physical activity monitoring devices as a research tool vs. commercial tool**
    i. Feedback to the patient is not required when using the devices for research.

6. **Sustainability of the physical activity engendered by the tool used in terms of:**
    i. Achieving physical activity goals
    ii. Maintaining physical activity levels, or even preventing a decline in physical activity once physical activity goals have been achieved may be a positive outcome in terms of an activity intervention.

iii. Is there a physical activity ceiling amongst children and young people with CF?

7. **Importance of accruing 7 days worth of physical activity data:**
    i. To help identify patients patterns of physical activity
    ii. To help identify intervention opportunities.

8. **Issues of compliance raised**
    i. What would make different groups of children and young people with CF comply (e.g. inactive/active)?
    ii. What prompts/incentives would be needed to increase compliance?

9. **Clinical barriers identified;**
    i. Cost implication involved as single use physical activity monitoring devices would need to be provided to children and young people with CF to prevention cross-infection.
    ii. Resources
    iii. Time
    iv. Educated, working in partnership of families would be needed.

10. **Team message is important**
    i. Although the physiotherapist is well-placed to deliver and facilitate monitoring, the clinical team needs to have input also.

Part 3: Research Issues

1. **Cost –** funding is needed to support further research in the area.

2. **Issues of compliance** – compliance needed to obtain meaningful data.

3. **Type of data produced by research vs. commercial devices**
    i. i.e. research outcome vs. user friendly
    ii. Further evidence is needed to validate commercial devices.

4. **Children and young people involvement required to inform the research process**
    i. Emphasising research with, as opposed to research on, children and young people.

5. **Literacy and understanding**
    i. CF children and young people are experienced with working with adults from a young age
    ii. Capitalise on this experience in research to gain better understanding of the issues affecting children and young people with CF.

Kevin’s priorities

Patient issues
2,5,7,6,1,3,4,8

Clinical practice issues
2,6,8,1,4,10,7,3,5,9

Research issues
4,3,5,2,1

Expert-Panel Meeting
(HCP-P)

Part 1: Patient-Related Issues

1. **Lifestyle based approach to physical activity**
   i. Parents as facilitators, including parental encouragement and the effects of direct and indirect positive reinforcement (e.g. being a role model by being involved in physical activity themselves) thus promoting physical activity engagement as a "normal part of life".
   ii. A lot of what is reported in terms of their perceptions of physical activity by the CF children and young people is seen in "mainstream interventions" among the non-CF population.

2. **Motivation is a key issue**
   i. Importance of goal setting related to physical activity.
   ii. Relationship between feedback from monitoring devices and subsequent impact on motivation highlighted
   iii. The role of monitoring device feedback, and its potential negative effects, for those not motivated to engage in physical activity
   iv.

3. **Experience of CF symptoms during physical activity**
   i. Children and young people perceived as being as capable as regards physical activity to that of non-CF children and young people.
   ii. Experience of symptoms should not be interpreted as a reason to stop or reduce physical activity.

4. **Decline of Physical activity**
   i. Teenage years seen as a pivotal point for onset of physical activity decline or adoption.
   ii. Pressures of academia, including time pressures, perceived as a barrier to physical activity.
   iii. Patients rebelling against parents, such that their encouragement for physical activity "backfires".
   iv. Chaotic lives of some children can influence their physical activity.

5. **Perceived importance of physical activity (and the data retrieved by the devices I think these are two separate points)**
   i. The perceived value of physical activity may be an important determinant of whether or not they will engage in physical activity monitoring.

ii. Whilst CF children are exposed to information concerning their condition from a very early age, the question of how CF children and young people will make sense of/interpret physical activity related data retrieved from the devices and assimilate this information into their everyday lives was raised

6. **Structured vs. non-structured activity**
   i. Perceptions of what "constitutes" normal activity/normal intensity physical activity in this clinical population.
   ii. Evidence from the mainstream population highlights the importance of parents understanding the physical activity guidelines, and being aware that physical activity is not just a case of engaging in structured or organised exercise, but can be accrued through discreet periods during the day (e.g. playtime, walking to school).
   iii. Similarly, a patient may engage in organised physical activity (e.g. football training), however they may only be active for a short period of time within that coached session as opposed to those who cycle to their friends house or play out with their friends.
   iv.
7. **Importance of clinical (lab based tests including V02 max) versus "field" testing of physical activity levels**
   i. Importance of shuttle test highlighted which is a field test
   ii. Importance of "field" tests highlighted to provide an idea of patient capabilities in a more realistic setting

8. **Importance of fitness over physical activity**
   i. Evidence from adult-based research emerging to suggest that fitness is related to improved outcomes among CF patients
   ii. Does fitness correlate with physical activity level in children and young people with CF?
   iii. HIT (High Intensity Training) approach – does this translate for children/young people with CF?

Part 2: Clinical Practice Issues

1. **Education**
   i. For the practitioner
   ii. For the parent
   iii. For the children and young people.

2. **Clinical barriers identified;**
   i. Cost implication involved as single use physical activity monitoring devices would need to be provided to children and young people with CF to prevention cross-infection.
   ii. Resources

iii. Time
iv. Educated, working in partnership of families would be needed.

3. **Team message is important**
    i. Although the physiotherapist is well-placed to deliver and facilitate monitoring, the clinical team needs to have input also.

4. **Issues of compliance raised**
    i. What would make different groups of children and young people with CF comply (e.g. inactive/active)?
    ii. What prompts/incentives would be needed to increase compliance?

5. **Importance of meaningful feedback**
    i. It is important that clinicians and patients are able to interpret the feedback delivered by the physical activity devices used.
    ii. A supportive network could be in place to facilitate the effective delivery of the feedback to patients
    iii. For example, it does not have to be a clinician, it could be offered by different people in different roles (e.g. counsellors).

6. **Role of feedback provided by devices**
    i. Analysis of data will highlight issues of non-adherence.
    ii. Provide a means of conceptualising physical activity (e.g. you've completed X steps, this compares nationally for mainstream children like this).
    iii. Provides a means of "prescribing" physical activity at a bespoke level.

7. **Sustainability of the physical activity engendered by the tool used in terms of:**
    i. Achieving physical activity goals
    ii. Maintaining physical activity levels, or even preventing a decline in physical activity once physical activity goals have been achieved may be a positive outcome in terms of an activity intervention.
    iii. Is there a physical activity ceiling amongst children and young people with CF?

8. **Importance of accruing 7 days worth of physical activity data:**
    i. To help identify patients patterns of physical activity
    ii. To help identify intervention opportunities.

9. **Testing vs. Monitoring**
    i. Distinction between the two is needed.
    ii. The importance of language used;
        For example, implementing a physical activity support package as opposed to testing, as the latter could be inferred as judgemental (e.g. patients may infer that they either “pass” or “fail” when their physical activity is monitored).

10. **Distinction between the use of physical activity monitoring devices as a research tool vs. commercial tool**
    i. Feedback to the patient is not required when using the devices for research.

Part 3: Research Issues

1. **Cost –** funding is needed to support further research in the area.

2. **Children and young people involvement required to inform the research process**
    i. Emphasising research with, as opposed to research on, children and young people.

3. **Issues of compliance** – compliance needed to obtain meaningful data.

4. **Type of data produced by research vs. commercial devices**
    i. i.e. research outcome vs. user friendly
    ii. Further evidence is needed to validate commercial devices.

5. **Literacy and understanding**
    i. CF children and young people are experienced with working with adults from a young age
    ii. Capitalise on this experience in research to gain better understanding of the issues affecting children and young people with CF.

Collective ranking

1. **Lifestyle based approach to physical activity**
    i. Parents as facilitators, including parental encouragement and the effects of direct and indirect positive reinforcement (e.g. being a role model by being involved in physical activity themselves) thus promoting physical activity engagement as a "normal part of life".
    ii. A lot of what is reported in terms of their perceptions of physical activity by the CF children and young people is seen in "mainstream interventions" among the non-CF population.

2. **Education**
    i. For the practitioner
    ii. For the parent
    iii. For the children and young people.

3. **Motivation is a key issue**
    i. Importance of goal setting related to physical activity.
    ii. Relationship between feedback from monitoring devices and subsequent impact on motivation highlighted
    iii. The role of monitoring device feedback, and its potential negative effects, for those not motivated to engage in physical activity

4. **Clinical barriers identified;**
    i. Cost implication involved as single use physical activity monitoring devices would need to be provided to children and young people with CF to prevention cross-infection.
    ii. Resources
    iii. Time
    iv. Educated, working in partnership of families would be needed.

5. **Team message is important**
    i. Although the physiotherapist is well-placed to deliver and facilitate monitoring, the clinical team needs to have input also.

6. **Experience of CF symptoms during physical activity**
    i. Children and young people perceived as being as capable as regards physical activity to that of non-CF children and young people.

ii. Experience of symptoms should not be interpreted as a reason to stop or reduce physical activity.

7. **Decline of Physical activity**
   i. Teenage years seen as a pivotal point for onset of physical activity decline or adoption.
   ii. Pressures of academia, including time pressures, perceived as a barrier to physical activity.
   iii. Patients rebelling against parents, such that their encouragement for physical activity "backfires".
   iv. Chaotic lives of some children can influence their physical
      1. activity.

8. **Issues of compliance raised**
   i. What would make different groups of children and young people with CF comply (e.g. inactive/active)?
   ii. What prompts/incentives would be needed to increase compliance?

9. **Perceived importance of physical activity**
   i. The perceived value of physical activity may be an important determinant of whether or not they will engage in physical activity monitoring.
   ii. Whilst CF children are exposed to information concerning their condition from a very early age, the question of how CF children and young people will make sense of/interpret physical activity related data retrieved from the devices and assimilate this information into their everyday lives was raised

10. **Importance of meaningful feedback**
   i. It is important that clinicians and patients are able to interpret the feedback delivered by the physical activity devices used.
   ii. A supportive network could be in place to facilitate the effective delivery of the feedback to patients
   iii. For example, it does not have to be a clinician, it could be offered by different people in different roles (e.g. counsellors).

11. **Structured vs. non-structured activity**
   i. Perceptions of what "constitutes" normal activity/normal intensity physical activity in this clinical population.
   ii. Evidence from the mainstream population highlights the importance of parents understanding the physical activity guidelines, and being aware that physical activity is not just a case of engaging in structured or organised exercise, but can be accrued through discreet periods during the day (e.g. playtime, walking to school).

iii. Similarly, a patient may engage in organised physical activity (e.g. football training), however they may only be active for a short period of time within that coached session as opposed to those who cycle to their friends house or play out with their friends.

12. **Cost –** funding is needed to support further research in the area.

This was very hard for me to rank. I have ranked it lower although I think the need for robust studies is really important. I feel some questions can be answered as part of daily clinical practice (not supported by funding) .

13. **Children and young people involvement required to inform the research process**
    i. Emphasising research with, as opposed to research on, children and young people.

14. **Role of feedback provided by devices**
    i. Analysis of data will highlight issues of non-adherence.
    ii. Provide a means of conceptualising physical activity (e.g. you've completed X steps, this compares nationally for mainstream children like this). Provides a means of "prescribing" physical activity at a bespoke level

15. **Sustainability of the physical activity engendered by the tool used in terms of:**
    i. Achieving physical activity goals
    ii. Maintaining physical activity levels, or even preventing a decline in physical activity once physical activity goals have been achieved may be a positive outcome in terms of an activity intervention.
    iii. Is there a physical activity ceiling amongst children and young people with CF?

16. **Type of data produced by research vs. commercial devices**
    i. i.e. research outcome vs. user friendly
    ii. Further evidence is needed to validate commercial devices.

17. **Literacy and understanding**
    i. CF children and young people are experienced with working with adults from a young age
    ii. Capitalise on this experience in research to gain better understanding of the issues affecting children and young people with CF.

18. Importance of accruing 7 days worth of physical activity data:

i. To help identify patients patterns of physical activity
ii. To help identify intervention opportunities.

19. Testing vs. Monitoring

i. Distinction between the two is needed.
ii. The importance of language used;
iii. For example, implementing a physical activity support package as opposed to testing, as the latter could be inferred as judgemental (e.g. patients may infer that they either "pass" or "fail" when their physical activity is monitored).

20. Importance of fitness over physical activity

i. Evidence from adult-based research emerging to suggest that fitness is related to improved outcomes among CF patients
ii. Does fitness correlate with physical activity level in children and young people with CF?
iii. HIT (High Intensity Training) approach – does this translate for children/young people with CF?

21. Distinction between the use of physical activity monitoring devices as a research tool vs. commercial tool

i. Feedback to the patient is not required when using the devices for research.

22. Importance of clinical (lab based tests including V02 max) versus "field" testing of physical activity levels

i. Importance of shuttle test highlighted which is a field test
ii. Importance of "field" tests highlighted to provide an idea of patient capabilities in a more realistic setting

Expert-Panel Meeting
(Researcher 1)

SECTION RANKINGS

Part 1: Patient-Related Issues

1. **Motivation is a key issue**
   - **i.** Importance of goal setting related to physical activity.
   - **ii.** Relationship between feedback from monitoring devices and subsequent impact on motivation highlighted
   - **iii.** The role of monitoring device feedback, and its potential negative effects, for those not motivated to engage in physical activity.
2. **Lifestyle based approach to physical activity**
   - i. Parents as facilitators, including parental encouragement and the effects of direct and indirect positive reinforcement (e.g. being a role model by being involved in physical activity themselves) thus promoting physical activity engagement as a "normal part of life".
   - ii. A lot of what is reported in terms of their perceptions of physical activity by the CF children and young people is seen in "mainstream interventions" among the non-CF population.
3. **Perceived importance of physical activity and the data retrieved by the devices**
   - **i.** The perceived value of physical activity may be an important determinant of whether or not they will engage in physical activity monitoring.
   - **ii.** Whilst CF children are exposed to information concerning their condition from a very early age, the question of how CF children and young people will make sense of/interpret physical activity related data retrieved from the devices and assimilate this information into their everyday lives was raised.
4. **Experience of CF symptoms during physical activity**
   - **i.** Children and young people perceived as being as capable as regards physical activity to that of non-CF children and young people.
   - **ii.** Experience of symptoms should not be interpreted as a reason to stop or reduce physical activity.
5. **Importance of fitness over physical activity**
   - i. Evidence from adult-based research emerging to suggest that fitness is related to improved outcomes among CF patients
   - ii. Does fitness correlate with physical activity level in children and young people with CF?
   - iii. HIT (High Intensity Training) approach – does this translate for children/young people with CF?
6. **Importance of clinical versus "field" testing of physical activity levels**
   - **i.** Importance of shuttle test highlighted.
   - **ii.** Importance of "field" tests highlighted to provide an idea of patient capabilities in a more realistic setting.

7. **Decline of Physical activity**
    i. Teenage years seen as a pivotal point for onset of physical activity decline or adoption.
    ii. Pressures of academia, including time pressures, perceived as a barrier to physical activity.
    iii. Patients rebelling against parents, such that their encouragement for physical activity “backfires”.
    iv. Chaotic lives of some children can influence their physical activity.
8. **Structured vs. non-structured activity**
    i. Perceptions of what “constitutes” normal activity/normal intensity physical activity in this clinical population.
    ii. Evidence from the mainstream population highlights the importance of parents understanding the physical activity guidelines, and being aware that physical activity is not just a case of engaging in structured or organised exercise, but can be accrued through discreet periods during the day (e.g. playtime, walking to school).
    iii. Similarly, a patient may engage in organised physical activity (e.g. football training), however they may only be active for a short period of time within that coached session as opposed to those who cycle to their friends house or play out with their friends.

Part 2: Clinical Practice Issues

1. **Testing vs. Monitoring**
    i. Distinction between the two is needed.
    ii. The importance of language used;
        1. For example, implementing a physical activity support package as opposed to testing, as the latter could be inferred as judgemental (e.g. patients may infer that they either “pass” or “fail” when their physical activity is monitored).
2. **Role of feedback provided by devices**
    i. Analysis of data will highlight issues of non-adherence.
    ii. Provide a means of conceptualising physical activity (e.g. you’ve completed X steps, this compares nationally for mainstream children like this).
    iii. Provides a means of “prescribing” physical activity at a bespoke level.
3. **Importance of meaningful feedback**
    i. It is important that clinicians and patients are able to interpret the feedback delivered by the physical activity devices used.
    ii. A supportive network could be in place to facilitate the effective delivery of the feedback to patients
        1. For example, it does not have to be a clinician, it could be offered by different people in different roles (e.g. counsellors).
4. **Clinical barriers identified;**
    i. Cost implication involved as single use physical activity monitoring devices would need to be provided to children and young people with CF to prevention cross-infection.
    ii. Resources
    iii. Time
    iv. Educated, working in partnership of families would be needed.
5. **Issues of compliance raised**
    i. What would make different groups of children and young people with CF comply (e.g. inactive/active)?
    ii. What prompts/incentives would be needed to increase compliance?
6. **Importance of accruing 7 days worth of physical activity data:**
    i. To help identify patients patterns of physical activity
    ii. To help identify intervention opportunities.
7. **Education**
    i. For the practitioner
    ii. For the parent
    iii. For the children and young people.
8. **Team message is important**
    i. Although the physiotherapist is well-placed to deliver and facilitate monitoring, the clinical team needs to have input also.

9. **Sustainability of the physical activity engendered by the tool used in terms of:**
    i. Achieving physical activity goals
    ii. Maintaining physical activity levels, or even preventing a decline in physical activity once physical activity goals have been achieved may be a positive outcome in terms of an activity intervention.
    iii. Is there a physical activity ceiling amongst children and young people with CF?
10. **Distinction between the use of physical activity monitoring devices as a research tool vs. commercial tool**
    i. Feedback to the patient is not required when using the devices for research.

Part 3: Research Issues

1. **Cost –** funding is needed to support further research in the area.
2. **Type of data produced by research vs. commercial devices**
    i. i.e. research outcome vs. user friendly
    ii. Further evidence is needed to validate commercial devices.
3. **Children and young people involvement required to inform the research process**
    i. Emphasising research with, as opposed to research on, children and young people.
4. **Literacy and understanding**
    i. CF children and young people are experienced with working with adults from a young age
    ii. Capitalise on this experience in research to gain better understanding of the issues affecting children and young people with CF.

5. **Issues of compliance** – compliance needed to obtain meaningful data.

OVERALL RANKINGS

1. **Motivation is a key issue**
   - **i.** Importance of goal setting related to physical activity.
   - **ii.** Relationship between feedback from monitoring devices and subsequent impact on motivation highlighted
   - **iii.** The role of monitoring device feedback, and its potential negative effects, for those not motivated to engage in physical activity.
2. **Cost –** funding is needed to support further research in the area.

3. **Testing vs. Monitoring**
   - **i.** Distinction between the two is needed.
   - **ii.** The importance of language used;
     - **1.** For example, implementing a physical activity support package as opposed to testing, as the latter could be inferred as judgemental (e.g. patients may infer that they either "pass" or "fail" when their physical activity is monitored).
4. **Lifestyle based approach to physical activity**
   - i. Parents as facilitators, including parental encouragement and the effects of direct and indirect positive reinforcement (e.g. being a role model by being involved in physical activity themselves) thus promoting physical activity engagement as a "normal part of life".
   - ii. A lot of what is reported in terms of their perceptions of physical activity by the CF children and young people is seen in "mainstream interventions" among the non-CF population.
5. **Perceived importance of physical activity and the data retrieved by the devices**
   - **i.** The perceived value of physical activity may be an important determinant of whether or not they will engage in physical activity monitoring.
   - **ii.** Whilst CF children are exposed to information concerning their condition from a very early age, the question of how CF children and young people will make sense of/interpret physical activity related data retrieved from the devices and assimilate this information into their everyday lives was raised.
6. **Role of feedback provided by devices**
   - **i.** Analysis of data will highlight issues of non-adherence.
   - **ii.** Provide a means of conceptualising physical activity (e.g. you've completed X steps, this compares nationally for mainstream children like this).
   - **iii.** Provides a means of "prescribing" physical activity at a bespoke level.
7. **Importance of meaningful feedback**
   - **i.** It is important that clinicians and patients are able to interpret the feedback delivered by the physical activity devices used.

    ii. A supportive network could be in place to facilitate the effective delivery of the feedback to patients
        1. For example, it does not have to be a clinician, it could be offered by different people in different roles (e.g. counsellors).

8. **Type of data produced by research vs. commercial devices**
    i. i.e. research outcome vs. user friendly
    ii. Further evidence is needed to validate commercial devices.
9. **Children and young people involvement required to inform the research process**
    i. Emphasising research with, as opposed to research on, children and young people.
10. **Experience of CF symptoms during physical activity**
    i. Children and young people perceived as being as capable as regards physical activity to that of non-CF children and young people.
    ii. Experience of symptoms should not be interpreted as a reason to stop or reduce physical activity.
11. **Importance of fitness over physical activity**
    i. Evidence from adult-based research emerging to suggest that fitness is related to improved outcomes among CF patients
    ii. Does fitness correlate with physical activity level in children and young people with CF?
    iii. HIT (High Intensity Training) approach – does this translate for children/young people with CF?
12. **Clinical barriers identified;**
    i. Cost implication involved as single use physical activity monitoring devices would need to be provided to children and young people with CF to prevention cross-infection.
    ii. Resources
    iii. Time
    iv. Educated, working in partnership of families would be needed.
13. **Issues of compliance** – compliance needed to obtain meaningful data.
14. **Issues of compliance raised**
    i. What would make different groups of children and young people with CF comply (e.g. inactive/active)?
    ii. What prompts/incentives would be needed to increase compliance?
15. **Importance of accruing 7 days worth of physical activity data:**
    i. To help identify patients patterns of physical activity
    ii. To help identify intervention opportunities.
16. **Importance of clinical versus “field” testing of physical activity levels**
    i. Importance of shuttle test highlighted.
    ii. Importance of “field” tests highlighted to provide an idea of patient capabilities in a more realistic setting.
17. **Education**
    i. For the practitioner
    ii. For the parent
    iii. For the children and young people.
18. **Literacy and understanding**

i. CF children and young people are experienced with working with adults from a young age
ii. Capitalise on this experience in research to gain better understanding of the issues affecting children and young people with CF.

19. **Team message is important**
    i. Although the physiotherapist is well-placed to deliver and facilitate monitoring, the clinical team needs to have input also.
20. **Sustainability of the physical activity engendered by the tool used in terms of:**
    i. Achieving physical activity goals
    ii. Maintaining physical activity levels, or even preventing a decline in physical activity once physical activity goals have been achieved may be a positive outcome in terms of an activity intervention.
        1. Is there a physical activity ceiling amongst children and young people with CF?
21. **Decline of Physical activity**
    i. Teenage years seen as a pivotal point for onset of physical activity decline or adoption.
    ii. Pressures of academia, including time pressures, perceived as a barrier to physical activity.
    iii. Patients rebelling against parents, such that their encouragement for physical activity “backfires”.
    iv. Chaotic lives of some children can influence their physical activity.
22. **Structured vs. non-structured activity**
    i. Perceptions of what “constitutes” normal activity/normal intensity physical activity in this clinical population.
    ii. Evidence from the mainstream population highlights the importance of parents understanding the physical activity guidelines, and being aware that physical activity is not just a case of engaging in structured or organised exercise, but can be accrued through discreet periods during the day (e.g. playtime, walking to school).
    iii. Similarly, a patient may engage in organised physical activity (e.g. football training), however they may only be active for a short period of time within that coached session as opposed to those who cycle to their friends house or play out with their friends.
23. **Distinction between the use of physical activity monitoring devices as a research tool vs. commercial tool**
    i. Feedback to the patient is not required when using the devices for research.

Expert-Panel Meeting
(Researcher 2)

Part 1: Patient-Related Issues

- **Lifestyle based approach to physical activity**
  - Parents as facilitators, including parental encouragement and the effects of direct and indirect positive reinforcement (e.g. being a role model by being involved in physical activity themselves) thus promoting physical activity engagement as a "normal part of life".
  - A lot of what is reported in terms of their perceptions of physical activity by the CF children and young people is seen in "mainstream interventions" among the non-CF population.

Commented [A1]: Patient priority 1
Overall priority 2

- **Decline of Physical activity**
  - Teenage years seen as a pivotal point for onset of physical activity decline or adoption.
  - Pressures of academia, including time pressures, perceived as a barrier to physical activity.
  - Patients rebelling against parents, such that their encouragement for physical activity "backfires".
  - Chaotic lives of some children can influence their physical activity.

Commented [A2]: Priority 9

- **Experience of CF symptoms during physical activity**
  - Children and young people perceived as being as capable as regards physical activity to that of non-CF children and young people.
  - Experience of symptoms should not be interpreted as a reason to stop or reduce physical activity.

Commented [A3]: PRI Priority 4

- **Importance of clinical versus "field" testing of physical activity levels**
  - Importance of shuttle test highlighted.
  - Importance of "field" tests highlighted to provide an idea of patient capabilities in a more realistic setting.

Commented [A4]: PRI Priority 5

- **Motivation is a key issue**
  - Importance of goal setting related to physical activity.
  - Relationship between feedback from monitoring devices and subsequent impact on motivation highlighted
  - The role of monitoring device feedback, and its potential negative effects, for those not motivated to engage in physical activity.

Commented [A5]: PRI priority 6

- **Perceived importance of physical activity and the data retrieved by the devices**
    - The perceived value of physical activity may be an important determinant of whether or not they will engage in physical activity monitoring.
    - Whilst CF children are exposed to information concerning their condition from a very early age, the question of how CF children and young people will make sense of/interpret physical activity related data retrieved from the devices and assimilate this information into their everyday lives was raised.

Commented [A6]: PRI issue 3

- **Importance of fitness over physical activity**
    - Evidence from adult-based research emerging to suggest that fitness is related to improved outcomes among CF patients
    - Does fitness correlate with physical activity level in children and young people with CF?
    - HIT (High Intensity Training) approach – does this translate for children/young people with CF?

Commented [A7]: PRI Priority 2

- **Structured vs. non-structured activity**
    - Perceptions of what "constitutes" normal activity/normal intensity physical activity in this clinical population.
    - Evidence from the mainstream population highlights the importance of parents understanding the physical activity guidelines, and being aware that physical activity is not just a case of engaging in structured or organised exercise, but can be accrued through discreet periods during the day (e.g. playtime, walking to school).
    - Similarly, a patient may engage in organised physical activity (e.g. football training), however they may only be active for a short period of time within that coached session as opposed to those who cycle to their friends house or play out with their friends.

Commented [A8]: PRI priority 7

Part 2: Clinical Practice Issues

- **Role of feedback provided by devices**
  - Analysis of data will highlight issues of non-adherence.
  - Provide a means of conceptualising physical activity (e.g. you've completed X steps, this compares nationally for mainstream children like this).
  - Provides a means of "prescribing" physical activity at a bespoke level.

Commented [A9]: CPI priority 5

- **Education**
  - For the practitioner
  - For the parent
  - For the children and young people.

Commented [A10]: CPI priority 2

- **Testing vs. Monitoring**
  - Distinction between the two is needed.
  - The importance of language used;
    - For example, implementing a physical activity support package as opposed to testing, as the latter could be inferred as judgemental (e.g. patients may infer that they either "pass" or "fail" when their physical activity is monitored).

Commented [A11]: CPI priority 9

- **Importance of meaningful feedback**
  - It is important that clinicians and patients are able to interpret the feedback delivered by the physical activity devices used.
  - A supportive network could be in place to facilitate the effective delivery of the feedback to patients
    - For example, it does not have to be a clinician, it could be offered by different people in different roles (e.g. counsellors).

Commented [A12]: CPI Priority 3

- **Distinction between the use of physical activity monitoring devices as a research tool vs. commercial tool**
  - Feedback to the patient is not required when using the devices for research.

Commented [A13]: CPI priority 10

- **Sustainability of the physical activity engendered by the tool used in terms of:**
  - Achieving physical activity goals
  - Maintaining physical activity levels, or even preventing a decline in physical activity once physical activity goals have

been achieved may be a positive outcome in terms of an activity intervention.
    - Is there a physical activity ceiling amongst children and young people with CF?

- **Importance of accruing 7 days worth of physical activity data:**
    - To help identify patients patterns of physical activity
    - To help identify intervention opportunities.

- **Issues of compliance raised**
    - What would make different groups of children and young people with CF comply (e.g. inactive/active)?
    - What prompts/incentives would be needed to increase compliance?

- **Clinical barriers identified;**
    - Cost implication involved as single use physical activity monitoring devices would need to be provided to children and young people with CF to prevention cross-infection.
    - Resources
    - Time
    - Educated, working in partnership of families would be needed.

- **Team message is important**
    - Although the physiotherapist is well-placed to deliver and facilitate monitoring, the clinical team needs to have input also.

Commented [A14]: CPI priority 6

Commented [A15]: CPI Priority 4

Commented [A16]: CPI priority 7

Commented [A17]: CPI Priority 1
Overall priority 3

Commented [A18]: CPI priority 8

Part 3: Research Issues

- **Cost –** funding is needed to support further research in the area.
- **Issues of compliance** – compliance needed to obtain meaningful data.
- **Type of data produced by research vs. commercial devices**
  - i.e. research outcome vs. user friendly
  - Further evidence is needed to validate commercial devices.
- **Children and young people involvement required to inform the research process**
  - Emphasising research with, as opposed to research on, children and young people.
- **Literacy and understanding**
  - CF children and young people are experienced with working with adults from a young age
  - Capitalise on this experience in research to gain better understanding of the issues affecting children and young people with CF.

Commented [A19]: RO priority 1
Overall priority 1

Commented [A20]: RO priority 2

Commented [A21]: RO priority 5

Commented [A22]: RO priority 4

Commented [A23]: RO priority 3
