# Supplementary material for: A formative study exploring perceptions of physical activity and physical activity monitoring among children and young people with cystic fibrosis and health care professionals
Source: BMC Pediatr. 2018 Oct 23;18:335. doi: 10.1186/s12887-018-1301-x (PMC6198445; doi:10.1186/s12887-018-1301-x)
Supplement: Supplementary file 9 — Phase 3 - P1–9 Transcripts.pdf. (PDF 670 kb) [file 12887_2018_1301_MOESM9_ESM.pdf]

Phase 3 – P1

P1 So hi, P2. If you remember the last time we met, I spoke to you about when you're active and when you're not active, and then I asked you to try out two different activity monitors, and one was the Actigraph, the one that went round your waist, and the other one was the pedometer, and that clipped onto your belt. I'm here today to ask you thirty short questions about what you thought about the devices you tried out, like what you liked about them and what you disliked about them. There's no right or wrong answers, so don't worry. I'm interested in what you have to say, and may I ask for some examples if you can, because this'll help me to understand your experiences better. If you need to think for a little bit before answering a question that's ok. We won't try and answer for you. We'll just wait until you feel ready to answer, or if we move past a question and then you remember something that you wanted to say, that's fine too. You can add things at the end. That's not to exclude your Mum. There are some specific questions for your Mum also, so watch out for these. It should take around forty minutes, depending on your answers, and I'll be recording our conversation on the Dictaphone just to help me remember everything we say. Also I'll be ticking off the questions that I ask as we go along so I don't say the same question twice, or if you or I happen to mention someone's name as we go through the questions, or anything that might let someone else know who you are or other people, I'll be taking this out of the transcript of our conversation, and this is just a document where we write out the conversation. If you're happy to begin?

P2 Yes.

P1 OK. In this first section I'm going to ask you about the times and places that you wore the activity monitors we asked you to wear. On the feedback questionnaire that you completed after wearing each physical activity monitor, you said that you agreed that you enjoyed wearing the physical activity monitors. Can you tell me a bit more about that?

P2 Well, on the clip-on one, it was fun, because one night when I went to footie training, when I was running round I just kept looking at it, and it was like, I hadn't really done that many steps, so...

P1 So was that the pedometer, was it?

P2 Yes, the clip-on one.

P1 And how about the Actigraph, so the one that went round your waist on the belt?

P2 You couldn't see how many steps you did, and that one did like twist and everything, didn't it?

P1 Oh, right.

P2 So I didn't like that, so...

P1 So what did you like about wearing the activity monitors?

P2 Well, on the clip-on one you could see how many steps you did and stuff like that, and then on the other one it got all like all your twists in, so it was like cool.

P1 What was that, sorry?

P2 It was like cool, because if you like bend down, like they'd get that, wouldn't they?

Phase 3 – P1

P1 Yes, it measures three different types of movement.

P2 Yes.

P1 So it was quite cool. And what did you dislike about the activity monitors, about wearing them?

P2 Maybe on the clip-on one, sometimes it'd fall off, so when I went to footie training I'd put it on my shorts, but then I'd put my underwear on there over it, and I'd tuck that part of my tee-shirt in so it wouldn't fall off, and then maybe on the other one, I don't know, maybe with the strap, when you tightened it, it got like a bit loose, so you had to always try and tighten it again.

P1 Ah, so was that a bit annoying?

P2 Yes.

P1 Aaah. Thanks for that, P2. what physical activity monitor did you enjoy wearing the most?

P2 Maybe the clip-on one, because it was fast and easy to get off, and you could see how many steps you did and all that.

P1 Ah, right. But that was kind of my next question, What was it about the pedometer you enjoyed compared to the Actigraph? So you could see how many steps you had done.

P2 Yes.

P1 And also you could take it on and off quite easy. Brilliant. On the feedback questionnaire you said that you agreed that the Actigraph and pedometer were comfy to wear. What was it about the Actigraph and pedometer that you felt was comfy to wear?

P2 I'd say maybe the clip-on one was just comfy just because it clipped on, and then the strap one, I don't know. I just think it didn't dig into you if you had it in the right position, and stuff like that.

P1 Yes. What was uncomfy about them?

P2 Maybe if the strap one, if you were just sitting in the car and your seatbelt was pressing on it, and that was like digging into you, something like that, or I don't know.

P1 That's ok. That's a really good answer. So when you were wearing a seatbelt, sometimes it'd dig into you. How about the pedometer?

P2 The small one?

P1 Yes. Yes, the small one. What was uncomfy about that?

P2 I don't know really.

P1 That's ok. That's fine. Thanks. you said that you strongly disagreed that the Actigraph and pedometer got in the way of what you were doing. Can you tell me a bit more about that?

P2 On the clip-on one, that didn't get in the way and stuff like that, and the strap one didn't get in the way if I played football or anything like that.

P1 Yes, For football and stuff like that. And can you give me an example of when the monitor got in the way of what you were doing?

P2 I don't know. I didn't think it ever got in the way really.

P1 Ah, that's fine. Thank you. And what would you do to change the pedometer and Actigraph if you could do anything to make it better?

P2 I don't know. It's like pretty simple, like just put the strap on. The other one was just put the clip on. Maybe like the clip-on one, it wasn't just clips on the front, it was clips underneath as well. You had to cut a hole in your trousers, and then put it on that way and that way.

P1 Oh, right. So maybe changing the way it's clipped on for the pedometer.

P2 Yes.

P1 And how about the Actigraph? Would there be anything that you'd change for that to make it better, if you could change anything?

P2 Not really.

P1 No. And did you ever have to be told to remove the monitors?

P2 Well, when I went in the shower, obviously, or when I went swimming or something like that.

P1 When you went swimming. And who told you to remove the devices?

P2 My Mum.

P1 And what reasons did they give you to remove the devices?

P2 Say like, "Take it off. You're getting in the bath now."

P1 OK, so it doesn't get wet?

P2 Yes.

P1 Yes. Oh, that's brilliant. Thanks. Next I'd like to talk about other people like friends and family, and how they may have influenced you to wear the physical activity monitors. Did you ever forget to put the physical activity monitor on?

P2 Well, no. That one, the small one, I don't think I ever, no I didn't, but I think one day with the big one, I think one day I didn't when I went to Stoke, and then one day I was going to my friend's house, not house, we went to this place called Madeleine Mere, and it's like all muddy there, and you could get like across water and stuff like that, so I didn't put it on then.

P1 Ah, right. And how come you didn't put it on then, when you went to Madeleine Mere?

P2 Because if I fell in the water, or something like that.

Phase 3 – P1

P1 I see. And how about when you went to Stoke. You said that you forgot to put it on then? Can you tell me a little bit more about that?

P2 I don't know. I think I was just in a hurry, I just didn't get it on.

P1 Those things happen, don't they? I always forget things when I'm in a hurry. On the feedback questionnaire you said that you strongly disagreed that you kept forgetting to put the physical activity monitor on. How was it for you remembering to wear the monitor each day?

P2 I don't know. I just think it feels like a natural thing, like say you're getting ready for school in a morning, it's like you get your tee-shirt on, and you just put it on basically, like natural.

P1 Oh yes. Sounds like you're saying it was part of your routine in the morning putting the monitor on. How was it for you remembering to wear the monitor each day?

P2 Yes.

P1 And that was my next question. Can you tell me how you remembered to wear the monitor each day?

P2 Yes, it was just like you just kind of remembered, because it was like part of what you do in the morning. It was like, get washed, get dressed, put your monitor on, go downstairs and get your bag sorted and all that, so I just remembered.

P1 Yes. Ah, brilliant. And you said that you agreed that other people noticed that you were wearing a pedometer. What did people say to you?

P2 I don't know, my friend, the small one that you could see the steps, like, "How many steps have you done now?" and I'd be like, "I don't know".

P1 So they'd ask you how many steps you'd done and things like that.

P2 Yes.

P1 What did you think about what they said?

P2 I don't know. It was like, "How many steps have you done?" I was just like, "Oh, I'll check, Two thousand and four". Yes.

P1 How did it make you feel when they were asking you?

P2 All right. Like it didn't bother me or anything.

P1 So it didn't bother you. Thanks. And did you notice a change in the amount of active activities you normally do as a family, while you were wearing the physical activity monitors?

P2 No.

P1 No? And how about with your friends? Did how your friends are involved in you being active change?

P2 No.

Phase 3 – P1

P1 No? So did the way that you play with your friends change while you were wearing the monitors?

P2 Well, I don't know really. No, not really, because we usually just sit there and talk.

P1 Yes. On the feedback questionnaire you said that disagreed that other people like friends or family kept asking how much activity you did while you were wearing the pedometer. What do you think about that?

P2 What do you mean?

P1 So you know on those feedback questionnaires, I think there's a question there, and it asks whether friends or family kept asking you how much activity you did while you were wearing the pedometer. I was just wondering what you thought about that, whether you've got any thoughts?

P2 Not really.

P1 No, not really? How about for the Actigraph? You agreed that other people like friends and family kept asking how much activity you did while you were wearing it. What do you think about that?

P2 Well, it was like, "How many steps have you done?" and I was like, "I don't know. You know you can't look on that one".

P1 And what do you think it was about the Actigraph compared to the pedometer that made friends and family keep asking you how much physical activity you were doing?

P2 Well, on the small one you could check, like you could see, so they asked me a bit more on that one, because you couldn't check. It was like, "There's no point asking, because we can't see".

P1 Aaah, I see. So it was more the other way round, people asked you when you were on the pedometer, rather than the Actigraph?

P2 Yes.

P1 Yes, so the Actigraph was the one that went round the waist.

P2 Yes.

P1 OK, thanks for that. OK, thank you for your answers. I'm learning a lot about what wearing the activity monitors was like for you. In this last section I'm going to explore how wearing the physical activity monitor may or may not have affected the activities that you do. Do you think that wearing a physical activity monitor encouraged you to be more active?

P2 Well, not really like. Well, maybe. I don't know. I just did like natural things I do.

P1 You just did the things that you normally do.

P2 Yes.

Phase 3 – P1

P1 OK. That's great. and how about other young people with CF? Do you think wearing the Actigraph and pedometer would encourage them to be more active?

P2 Yes, probably.

P1 Probably? Can you tell me a little bit more about that?

P2 Maybe because they want to know how many steps they've done in a day. So yes.

P1 And which device or which monitor do you think would be better for other people with CF out of the ones that you tried?

P2 Maybe like how I started. I did it with the small one first, so maybe that one, and then if you want to, you can do the strap one.

P1 OK, so maybe start off with the pedometer, and then if you wanted a little bit more information you could go onto the Actigraph.

P2 Yes.

P1 OK. And how do you think using the Actigraph and pedometer could help you to be more active?

P2 Maybe, say on Monday you do on the small ones, and you do one thousand steps, like you could say, "Tomorrow I want to get two thousand steps," and like push you to get a goal.

P1 Oh yes, so like pushing you on to do like bigger goals or do more steps.

P2 Yes.

P1 OK, that's brilliant. And how about the Actigraph? How do you think that could help you to be more active?

P2 I don't know.

P1 That's fine. You're doing really well. Thanks. Can you tell me how you think using the Actigraph and pedometer was useful or not useful at encouraging physical activity?

P2 I don't know.

P1 OK. What was it about the Actigraph and pedometer that you feel encouraged you to be more active?

P2 Well, I still did like the natural things I did, stuff like that.

P1 Yes. Ah, so maybe it didn't encourage you to be more active. You just did what you normally do.

P2 Yes.

P1 That's great. And what was it about the Actigraph and pedometer you feel stopped you from being active?

P2 Well, it didn't really stop me. I still went to dance and stuff with it on.

P1 Yes.

P2 And everything, so it didn't stop me.

P1 Yes. Ah, that's brilliant. Thanks for those answers. You can have a little rest. I'm just going to ask your Mum a couple of questions if that's all right.

P3 Yes, that's fine.

P1 I'm just going to ask you some questions if that's ok. What do you think the benefits of wearing a physical activity monitor are for P2?

P3 I think she's curious about, she knows that she's really active, but she is quite curious. P2 likes measuring things, and she likes challenging herself, so like she said with the pedometer, you did that, didn't you? If you did so many steps you'd go, "I'm going to see if I can do more tomorrow", and so I think that was good.

P1 Yes, so it'd be like goal setting, and things like that.

P3 Yes.

P1 And what do you think the negatives of wearing a physical activity monitor are for P2?

P3 I think only just remembering, and we only forgot once, I think. It's like for tablets, you know, and all the rest that goes with CF. You forget it very occasionally, because you're human, but I think once it becomes part of your normal routine, then it's just something you do every day, so I suppose that's the only negative, that you do have to remember it, but other than that, I don't there were.

P1 That's brilliant. Model participant.

P3 Yes, she is.

P1 And thinking about P2, do you feel that the activity monitors encouraged her to be more active?

P3 Yes. Well, she is really active anyway, like I say, so it's hard, but yes, I do think they did. I think if she was getting to the point where she was a little less eager to, you know, a bit more reluctant with her exercise, it would give her the push, that she'd have a look and go, "Well, I'm not doing that much, so yes, let's do a little bit more" Yes.

P1 Yes. Ah, thanks. And what is it about the activity monitors that P2 tried out that you feel encouraged her to be more active? So she tried the pedometer and the Actigraph.

P3 Yes. I think the visual of the pedometer's good, and being able to see, and being able to measure for herself. I think that was good. I think the other one, if there's a way, if it was a regular thing wearing it, if there was a way of getting feedback, that would be different, but I think you haven't got the visual straight away, the immediate visuals, so...

P1 Yes. So with the Actigraph you haven't got that visual, whereas with the pedometer you have.

P3 Yes.

P1 Yes? And do you think it's feasible that for P2, wearing a physical activity monitor might influence the amount or type of activity that she does?

P3 Yes, I think so. I think if it was something that she did, if she had to do all the time, it would definitely influence, because you'd know when, I think as well, if she's had a lazy day in the holidays, and sat watching TV quite a lot, I know she'd look and say, "Ooh, I haven't done much today", so in the evening she'd be out and about playing football and doing extra stuff, just to make up for that, so yes.

P1 Sounds like your saying it'd act as a bit of a prompt.

P3 Yes, I think it would, yes.

P1 Ah, that's brilliant. Thanks very much. Back to you, P2, if that's ok. Last few questions now. You're doing really, really well. Thank you very much. So the Actigraph shows that on average you did a hundred and eighty-nine minutes, which is three hours and nine minutes of moderate to vigorous activity each day. It's recommended that children and young people should do sixty minutes of moderate to vigorous activity each day to stay healthy. You did more than this. What do you think about that?

P2 I don't know.

P1 I can see your face.

P2 Yes, because some people are just lazy.

P1 Some people are just lazy?

P2 They should admire me.

P1 I can see a big smile on your face when I said how many minutes you did. How did it feel hearing that?

P2 I don't know. Proud.

P1 Proud? You should be proud. You've done really, really well. How did you find doing that much activity?

P2 It was all right. Like good.

P3 I think what's really interesting in that is that that was in holiday time.

P1 Yes, it was.

P3 So if you put it on in school time I think you'd be like...She never keeps still in school.

P1 Yes.

P3 So I think it's really interesting that that was in holiday time.

P1 Yes. Oh, so that's a good indicator really, isn't it, that you're still doing triple the recommended amount even in holiday time.

P3 And I think it's good for us to be able, sorry interrupting on your questions, P2. I think it's good, because we can correlate with some hard evidence. We do believe, and the team believe, that P2 is well because of her physical activity levels, but that's given her the evidence that her physical activity levels are way up there, and that probably is the most influencing factor.

P1 How do you feel, hearing what your Mum said? What do you think about that?

P2 Good.

P1 Good? Do you think your Mum's right, or do you think your Mum's wrong?

P2 I think my Mum's right.

P1 Yes? So it sounds like you maybe agree with what your Mum's saying?

P2 Yes.

P1 Brilliant. So the Actigraph shows that you did the most moderate to vigorous activity on Wednesday. What do you think helped you to be more active on this day?

P3 That's quite a while ago.

P2 Yes. I don't really know.

P1 You don't really know. So it was the school holidays. I think it might have been half term when you wore the Actigraph. Did you do anything different on that day than you normally do?

P2 I can't remember.

P1 No. That's fine. I don't think I could remember either.

P3 Do you know what, from what I remember, we didn't either. It wasn't particularly a... We didn't go anywhere special or do anything special. It was just a normal day.

P2 Wednesday. Dance!

P3 No dance, with them being off.

P1 So no dance during the holidays. You only do dance during term time. OK. So the Actigraph shows that on average you spent four hundred and eleven minutes, which is six hours fifty-one minutes, being sedentary, which means doing things while you're still, like sitting and watching TV. what do you think about that?

P2 I don't really know. Yes.

Phase 3 – P1

P1 Yes. So being sedentary means being still and well, I was just thinking, you've done on average about six hours fifty-one minutes, which is fine, and I was wondering what you thought about that. Have you got any thoughts?

P2 No, not really.

P1 Not really? That's fine. And the Actigraph shows that you were more sedentary on Tuesday and Thursday. What do you think about that?

P2 Does sedentary mean still?

P1 It means just like you could be sitting watching telly, or having a little rest, lying down, playing on computer games, that type of thing.

P2 Yes. I don't know. I was probably playing FIFA or something.

P1 Yes.

P2 Like I always do.

P1 Do you like FIFA?

P2 Yes. As soon as I wake up in the morning you do it. She comes down at probably about seven and does FIFA.

P1 Oh, so is FIFA your favourite game?

P2 I like Minecraft.

P1 Oh, I've heard about Minecraft. I don't really know what it is, though. Is it good?

P2 It's a game. It's good.

P1 Is it? And what was different about Tuesday and Thursday compared to Wednesday when you were most active?

P2 Well, on Tuesday and Thursday I was kind of still, but on Wednesday I was like hypo. I don't know. I must have just done an easy day, and then I must have pushed myself at Wednesday.

P1 Yes. Oh no, that's fair enough. That's the school holidays. Thanks, P2. Did you change the amount of activity you normally do while you were wearing the physical activity monitors?

P2 No, not really. I think one of them I had to stop dance, because it was like for half term.

P1 Oh, yes.

P2 Yes, but not really, though.

P1 So you stopped dance, but apart from that, not really. Can you give me an example of how the physical activity monitor made a difference to the activities you do?

P2 What do you mean?

Phase 3 – P1

P1 So I was just wondering did wearing the physical activity monitors make a difference to how much activity you did?

P2 No, not really.

P1 No? Ah, thanks. And on the feedback questionnaire you said that you disagreed that you tried new activities out while you wore the activity monitors. Can you tell me a bit more about that?

P2 Well, I do drama and modern on a Monday, and nothing on Tuesday, and I do tap and jazz on a Wednesday, nothing on a Thursday, training on Friday, and then the season's stopped now. Like I don't have matches on a Saturday any more training on Friday, and then the season's stopped now. Like I don't have matches on a Saturday any more, because the season's stopped, but yes, because I just kept like that, I didn't do any more activities or anything like that.

P1 You've got a routine that you stick to, no matter what.

P2 Yes.

P1 Ah, that's brilliant. And did you notice a change in how strenuous the activities you typically do are, like how fast you ran when you were playing football?

P2 No, not really. Well, I get to the line as fast as I ran.

P1 Yes. Brilliant. And can you tell me how it was for you seeing how many steps you did each day, you know, when you were using the pedometer?

P2 Yes, it was good, like surprising, because you were like, "Oh, have I really done that many steps?" But yes.

P1 So it was good and surprising. What was good about it?

P2 It was like, maybe you've set a goal, and then you've done it, you would be like, "Oh, yes. OK, tomorrow I'm going to do something else. Yeah, yeah, yeah, beat my goals" like that.

P1 And what was surprising about it?

P2 Like maybe if you've just been sitting on the couch all day, and then maybe in the afternoon I'll go out and play footie with my sister or something, you'd be like, "Did I really do that many steps just by running round in like the garden?"

P1 Sounds like that surprised you a little bit, how much you can do just by running round.

P2 Yes.

P1 Ah, that's brilliant, P2. And what did you like about seeing how many steps you did each day while you were using the pedometer?

P2 Well, I liked it because it was maybe like interesting, because I'd just keep sitting there looking at it, seeing how many I do, and then you'd just be running round, you'd just be like, "Two thousand and ninety-nine, and I'd be like, "Three hundred and ninety-six". I just kept looking at it, and yes, just interesting.

P1 Really interesting. And what did you dislike about seeing how many steps you did each day?

P2 I didn't really dislike anything.

P1 No. And what would you do to make the pedometer better if you could do anything at all?

P2 Not much.

P1 Not much? And what other information would you like to see on the pedometer apart from the number of steps that you do?

P2 The small one?

P1 The small one, yes.

P2 I don't know. Maybe it could do, like when you sit down, like how long you've sat down for, and then how long you stand up for, and then how long, like if you go running, obviously if I say I do three hundred steps a day, I'm not going to kept running non-stop until I've got to that number. I've obviously had breaks and just did what I mainly do, so yes.

P1 So you're saying that it might be good if you had...

P2 If it said like how long you've been running for, like one section. Do you get what I mean?

P1 Oh, yes. So like every time you ran, like how long you ran for and how many steps you did in that run, rather than the total amount of steps that you did in one day.

P2 Yes.

P1 I see. So maybe break it down a little bit.

P2 Yes. So like you'd see how many rests you had, and how long you sat on the couch for, and stuff like that.

P1 Oh, that's a really good idea. Thanks, yes. Brilliant. And how about the Actigraph, you know, the one round your waist? What would you do to change that if you could do anything?

P2 I don't know. Maybe you could like open it, if you get what I mean. Like on it, because it's like that thick, maybe you could have like another little layer on top that you could just open, and it tells you like how many steps, like all the stuff it did, like you know how it turned, it told you like that on it.

P1 Yes. So that you could actually see the information, rather than it be kept secret?

P2 Yes.

P1 Brilliant. And you mentioned on the feedback questionnaire that you agreed that getting some information each day would encourage you to do more activity. Can you tell me a bit more about that?

P2 Maybe like seeing it, you can set like your goal, which encourages you, like seeing it, so like the one that you couldn't see, it's like you don't know if you've set your goal or anything, and you

don't know if you're doing good or bad. But on the thingy one, you could be like, "Oh, I'm not doing good today", but something like that, if you get what I mean. It encourages you, because you can actually see it.

P1 Yes.

P2 Do you know what I mean?

P1 So it's not like the other one, the Actigraph. You don't get to see it, so you don't know what you're doing, do you? So on the pedometer it counts what you're doing.

P2 And you can set your goals.

P1 Ah, I see, so maybe make yourself do more.

P2 Yes.

P1 Or do less, if you're doing too much.

P2 Yes.

P1 That's brilliant. And what was it about getting some information that tells you how active you are each day you feel would encourage you to be more active? So you've actually answered that, haven't you? I don't really need to ask that question, because you were saying by setting your goals.

P2 Yes.

P1 Is there anything more you'd like to add to that one?

P2 No, not really.

P1 No? And you said that you'd like to wear an Actigraph and a pedometer again. Can you tell me a bit more about that?

P2 It was just interesting, like seeing how much I actually did and everything. Yes. And because some people might want, it might make other people better, like healthier type of thing.

P1 OK.

P2 Just like running, and trying to set goals and stuff like that.

P1 So it might make other people healthier as well.

P2 Yes.

P1 Can you tell me a bit more about that?

P2 It was just interesting, like seeing how much I actually did and everything. Yes. And because some people might want, it might make other people better, like healthier type of thing.

P1 OK.

P2 Just like running, trying to set goals and stuff like that.

P1 So it might make other people healthier as well.

P2 Yes.

P1 Can you tell me a bit more about that?

P2 Like if they did it.

P1 Ah, so if they used it.

P2 Yes.

P1 And how do you think it would make them more healthy?

P2 Because if they were into like set goals.

P1 Ah, I see.

P2 Yes.

P1 Ah, so for goal setting it might encourage people to be more active.

P2 Yes.

P1 Yes? Oh, that's brilliant. Thanks very much. If you could wear either one of those activity monitors again, which one would you prefer to wear?

P2 I like the little small one.

P1 The pedometer?

P2 Yes.

P1 Yes. OK. Last question now. If you could ask the clinicians, so the people at Alder Hey in the CF team, in the CF team, anything, what would you like to ask them?

P2 I don't know. Maybe... Like, anything?

P1 Anything at all.

P2 Well, I think I've already asked when I went to clinic last time I asked them can anyone take a Creon that didn't need it.

P1 Oh, right. And what was the answer?

P2 Yes.

P1 Oh, really?

P2 It wouldn't do any harm to them.

P1 Oh, I see. It's good to know these things though, isn't it? Is there anything else you'd like to ask them?

P2 No, not really.

P1 Not really? That's absolutely fine. You've been absolutely wonderful. Thank you very much, P2. Thank you for speaking with me today. I've really enjoyed hearing what you thought about wearing physical activity monitors. That's the end of the interview now. Is there anything more you'd like to add?

P2 No, not really.

P1 Maybe something I've missed? No? How about you, Mum?

P3 No, I don't think so. I'm quite happy with it. I know she enjoyed being part of it, didn't you, P2?

P2 Yes.

P3 She enjoys being part of any research. We do all sorts of research.

P2 Yes.

P3 Weeing in pots, blowing in bags.

P1 Ah, you are a star. Thank you very much, P2. I really appreciate that. I'll end the interview now.

End of Interview

Phase 3 - P2

P1 So hi, P1. If you remember from the last time we met, I spoke to you about when you're active and when you're not active, and then I asked you to try out two different activity monitors. One was an Actigraph, and the other was a Nike Fitbit. I'm here again today to ask you twenty-eight questions about what you thought about the devices you tried out, like what you liked about it and what you disliked about them. There's no right or wrong answers, so don't worry. I'm interested in what you have to say, and may I ask you for some examples if you can, because this will help me to understand your experiences better. If you need to think for a little while before answering a question that's ok. We won't try and answer for you. We'll just wait until you feel ready to answer, or if we move past a question and then you remember something that you wanted to say, that's fine too. You can add things at the end. That's not to exclude you, Mum. There are some specific questions for you also, so watch out for these. It should take around forty minutes, depending on your answers, and I'll be recording our conversation on the Dictaphone just to help me remember everything we say. Also I'll be ticking off the questions that I ask as we go along, just so I don't say the same question twice. If you or I happen to mention someone's name as we go through the questions, or anything that might let someone else know who you are or other people, I'll be taking this out of the transcript from our conversation, and this is just a document where we write out our conversation. So if you're happy to begin.

P2 Yes.

P1 OK. In this first section I'm going to ask you about the times and places that you wore the activity monitors we asked you to wear. So on the feedback questionnaire that I asked you to complete after wearing each device, you've said that you didn't agree or disagree that you enjoyed wearing the Actigraph. Can you tell me a bit about that?

P2 Well, I liked to wear the wrist one because it was a lot comfier than the one round my waist, and the one that was on my wrist, I just preferred that more than I preferred the waistband one.

P1 OK. So you said that you preferred the Fitbit over the Actigraph one, which was the one round your waist. What did you like about wearing those activity monitors, the Actigraph and the Fitbit?

P2 Well, I liked wearing the wrist one because I just liked it more than the other.

P1 OK, so you liked it a bit more than the other.

P2 Because I knew what I was doing with that one more than with the other one.

P1 And what was it about the other one that you didn't know what you were doing?

P2 Well, it didn't have anything for me to do on it, but it kept falling down at my waist and then making my waist really itchy and stuff, so that's why I didn't really like that one.

P1 OK. You didn't really like that because of that. Was there anything about the Actigraph that you did like?

P2 No.

P1 That's ok. That's absolutely fine. And what was it about the Fitbit that you liked?

P2 I can't really think, because I just preferred it more than I did with the waist one, because when I was wearing the watch one I knew how I was doing, because the first time I used it I came in, and it had already said on the computer how many steps I had done, and when I tap it, it would already just tell me what I've done and stuff, how many I've done.

P1 Ah, I see. So you liked the way it gave you some feedback on what you were doing.

P2 Yes.

P1 You've kind of answered my next question also, which was what physical activity monitor did you enjoy wearing the most, and would I be right saying that was the Fitbit monitor?

P2 Yes.

P1 OK. And what was it about the Fitbit that you enjoyed, compared to the Actigraph?

P2 Sorry, could you say that again?

P1 Yes, course. What was it about the Fitbit that you enjoyed, compared to the Actigraph?

P2 Well, the Fitbit, I enjoyed that because it would give me the feedback of what I've been doing, but with the Actigraph I couldn't see anything what I was doing, and it would just keep falling down.

P1 OK. Thank you very much, P1. On the feedback questionnaire you said that you disagreed that the Actigraph was comfy to wear, and that you didn't agree or disagree that the Fitbit was comfy to wear. What was it about the Actigraph and Fitbit that you felt made it not comfy to wear?

P2 Well, the Fitbit, the reason I don't really like that one is just it kept falling around, but it was a lot more better than the waist one. And with the waist one, I didn't like it because that kept falling down on me, and whenever I was running or anything, it'd just keep getting in the way and kept moving about.

P1 OK. So the Fitbit, it sounds it was a little bit loose.

P2 Yes. But it was a lot better than wearing the Actigraph.

P1 OK. Thank you very much. Oh well, you've kind of answered my next question as well, which was what was it about the Fitbit compared to the Actigraph that made it more comfy to wear, and was it just that it was not as loose as the Actigraph?

P2 Yes.

P1 OK, thank you. You said that you agreed that the Actigraph got in the way of what you were doing, whereas the Fitbit did not get in the way of what you were doing.

P2 Yes.

P1 Can you tell me a bit more about that? You've touched on it a little bit, but if you could tell me a little bit more.

P2 Well, whenever I was doing my sports or whatever in school, that kept on getting in the way, whereas wearing the Fitbit is basically just like wearing a watch or whatever, but with the, I've forgot the name if it...

P1 So the Actigraph.

P2 Yes, the Actigraph. I didn't like wearing that because whenever I'd be on the field doing PE or whatever, it'd always get in my way, because I'd always have to keep pulling it up and stuff.

P1 Oh, right, so you couldn't adjust it. OK. Can you give me an example of when the monitor, so the Actigraph, got in the way of what you were doing?

P2 Well, when I was running about, it kept twisting, so it'd be from being here to in the very front, and I kept having to twist it, and it was getting in the way of what I was really doing, because if I was running that'd stop me, because I have to keep twisting it and that.

P1 So did you have to actually stop your running just to fix it again?

P2 Yes.

P1 How was that for you, doing that?

P2 Well, it was a bit annoying at times, but I mean, I was pretty fine with it.

P1 Thank you very much. And where were you when you were doing the activities and it was getting in your way?

P2 Well, with the Fitbit I was just out and about with my friends, and then with the Actigraph, I was in school doing my PE, and it kept getting in the way.

P1 So did the Fitbit get in your way also, when you were doing your activities?

P2 No. The Fitbit was basically just like wearing a watch, but the Actigraph kept getting in the way of when I'd be doing my PE and stuff.

P1 OK. Thank you. What would you do to change the Actigraph and the Fitbit, if you could do anything to make it better?

P2 I don't know.

P1 So would there be anything that you would change? So maybe if we think about the Actigraph first. Would there be anything about the Actigraph that you'd change to make it better for you?

P2 Just get a smaller one, but not have it too tight or too loose around my waist. And then with the Fitbit, just tighten that up, but whenever I'd tighten it, it'd leave marks around. But then with that on, I could never turn it around, because with the Fitbit, when it was on my wrist, I kept twisting it round, because whenever I was doing running I would keep getting sweaty, and then it would be getting really itchy, so I'd have to keep twisting it round.

P1 Ah, so what do you think was making you itchy with the Fitbit? So it sounds like you're saying that when you were exercising, you either had to have it a little bit loose so it didn't stick and dig in you ...

P2 Yes, that's it, but when I was out everywhere else, like on my bike or whatever, I'd just have it not too tight and not too loose, so it wouldn't keep twisting about and then getting in the way.

P1 OK. Thanks very much for that. Did you ever have to be told to remove the monitor, either the Actigraph or the Fitbit?

P2 No.

P1 OK. So next I'd like to talk about other people, like friends and family, and how they may have influenced you to wear the physical activity monitor. Did you ever forget to put the physical activity monitor on?

P2 Yes.

P1 Yes? Could you tell me a bit more about that?

P2 Well, at times I'd get up in the morning, but when I'd still be a bit tired I would keep forgetting to put it on and stuff.

P1 Yes.

P2 Whereas with the Fitbit, it would basically just be like wearing a watch, so I'd always remember that, but with the other one, I don't know why, but I just kept not remembering to put it on.

P1 Ah right, ok. And on the feedback questionnaire you said that you disagreed that you kept forgetting to put the Actigraph on, but agreed that you kept forgetting to put the Fitbit on. How was it for you, remembering to wear the monitor each day?

P2 As in how?

P1 Just, you know, did you have a specific way that you remembered to put it on, for example, or...?

P2 No.

P1 No? Well tried. Thank you. Can you tell me how you remembered to wear the monitor each day?

P2 Well, after I'd worn it for about a few days, before the time was finished when I had finished them, it just kept getting used to me, so I'd stop forgetting to put it on, and then it would just always come to me.

P1 Ah, so it sounds like it became like part of your morning routine or what have you.

P2 Yes.

P1 Can I just go back to that last question, when I said how was it for you remembering to wear the monitor each day? I kind of phrased that a little bit wrong. I was just wondering how it felt for you having to remember to wear the monitor each day.

P2 Well, sometimes I would forget to remember to put it on, sometimes I'd just come down and get it.

P1 OK. You said that you agreed that other people noticed that you were wearing a Fitbit. What did people say to you?

P2 Nothing really, because one of my other mates had one as well, because when we were in our drama lesson, he had one on as well as a watch. But I was like, "That's the exact same as what I've got", but he'd always tap it as well, so we were basically the same.

P1 Did you notice a change in the amount of active activities you normally do as a family while you were wearing the physical activity monitors?

P2 No.

P1 No, ok. And how about your friends? Has how your friends are involved in you being active changed?

P2 No.

P1 No? OK. Did the way you play with your friends change while you were wearing the monitor?

P2 No, I'd just be careful and not be too lively. I'd just, I can't really describe it, but if it was just a little bit mad, then I wouldn't really do it.

P1 And was that while you were wearing both the Actigraph and the Fitbit?

P2 Yes, just so I wouldn't damage them.

P1 Ah, oh well, thank you for that. That was very considerate of you. That's really kind of you. Can you give me an example of when that might have happened?

P2 Well, usually, when we go on the field, we always just run after each other, like taking each other out, and basically sparing each other down, but I wouldn't go too mad on doing that compared to what I normally do. I just always do that, and then if I take someone down, I won't just damage whatever it is.

P1 What's sparing down?

P2 Basically rugby tackling.

P1 Oh, right!

P2 Basically just rugby tackling people to the ground.

P1 OK. Can I just go back to that question? What did you dislike about changing the way that you played, or got involved with your friends?

P2 Nothing, really.

P1 You know, the way you said that you had to use a little bit of caution and that. How was that for you? You know, how does that make you feel?

P2 All right. I wasn't too fussed about it.

P1 OK, thanks. On the feedback questionnaire you said that you disagreed that other people like friends or family kept asking how much activity you were doing while you were wearing the physical activity monitor. What do you think about that?

P2 Well, I was fine with it. I was fine with wearing it, or whatever.

P1 Yes. Was there anything that you wished people had said to you different?

P2 No, because they all knew in the first place I was getting it, because I let them know.

P1 Yes, ok. Thank you. OK, thank you for your answers. I'm learning a lot about what wearing physical activity monitors was like for you. In this last section I'm going to explore how wearing a physical activity monitor may or may not have affected the activities you do. Do you think that wearing a physical activity monitor encouraged you to be more active?

P2 No, I just kept doing the normal sports I was doing, and that was keeping me active.

P1 OK. Sorry, what's that last thing you said?

P2 Well, the sports I'm normally doing are the ones I'm always doing, and when I had the Fitbit and the others on, then nothing changed. I was just doing the sports I normally do, but being a little bit careful with what I was doing.

P1 And how about some young people with CF. Do you think wearing an Actigraph and/or a Fitbit would encourage them to be more active?

P2 Well, for my opinion, I'd just say if they knew everything about what it was, because at first I didn't really get to know much about it until we found out more stuff, and then I was just doing a bit more each time with them, and do good for it. And with me, I would have just said just try doing different stuff each time, so it'll be better for you, and then you'll be learning new stuff.

P1 Ah, so you'll say to other young people with CF just to maybe try different things?

P2 Yes.

P1 And would that be while they were wearing an activity monitor?

P2 Well, whenever really, just finding out new sports to do and stuff.

P1 Ah, so just general advice.

P2 Yes.

P1 And do you think that wearing a physical activity monitor would encourage other young people with CF to be more active?

P2 It depends really on what they thought about it, but I thought that it was all right to wear. I weren't really too bothered wearing it, because I knew why I have to wear it and stuff, but with other kids and stuff, until they actually got to know a lot more about it they'd be like, "Why do I have to wear this?" and everything, but as they knew more about what the Actigraph and Fitbit were about, then I think they'd start doing a lot more sport, and getting it.

P1 You just mentioned there that you didn't know much about it. I was wondering what it was that you remember about that.

P2 Well, at first I didn't really understand about what it was until around I knew that it is checking the sports and how active I am, and knew that was what they was both for. And then when I wore the Fitbit the first day I came back in with it, I just found out how many steps I had done and stuff.

P1 Ah, right.

p2 And then I was even for getting them all on, like all the little line numbers, and I did.

P1 Ah, so maybe you didn't know really much about the functions and things.

P2 Yes.

P1 And was that about the Fitbit or the Actigraph, or both?

P2 Both, to be honest. I mean, when with the Actigraph I didn't see what that was measuring or whatever it was showing up what I was doing, but with the Fitbit I always knew how well I was doing, because whenever I'd just tap it, it'd tell me.

P1 Ah, thank you.

P2 But I always knew if I had done good on the Fitbit, because when it would come up it'd always vibrate.

P1 And how did that feel for you when you got that sort of vibrate?

P2 Well, I just knew I was doing it correctly, and that's it really.

P1 How did it feel, knowing you were doing it correctly?

P2 Great.

P2 Ah, thank you. OK. And how do you think using the Actigraph and Fitbit could help you to be more active?

P2 I don't know really, because all the sports I do, I think are quite active for me and stuff.

P1 Can you tell me a bit more about that? You say all the sports you're doing are active.

P2 Well, I wouldn't really change with my sport, because the sports I'm doing now are just normal sports, like going out with my friends, running about, doing PE and so on.

P1 Ah, so you wouldn't change anything. OK, thanks. Can you tell me how you think using the Actigraph and Fitbit were useful or not useful at encouraging physical activity?

P2 As in how?

P2 So what was it about the Actigraph and Fitbit that you feel encouraged you to be more active?

P2 Nothing really, because all the activities I was doing keep me active.

P1 OK. What was it about the Actigraph and Fitbit that you feel stopped you from being active?

P2 The Actigraph just kept getting in the way, not getting in the way of what I was doing, but kept getting on my nerves when it kept slipping down and moving places. But with the Fitbit, I was fine with that.

P1 Did it happen often, that, when it was slipping down, the Actigraph?

P2 Yes, because it'd keep slipping, because when I'd be getting sweaty it'd just keep slipping down and making it really itchy for me and annoying, so I'd have to keep pulling it, and then just letting in some air. So that's all.

P1 Ah, thanks for that. OK, so you can have a little breather. I'm just going to ask your Mum some questions if that's ok. What do you think the benefits of wearing a physical activity monitor are for P2?

P3 I don't know if it was much benefit at all with the Actigraph, but as I said with the Fitbit, he knew when it vibrated. He knew, and I think it encouraged him a bit more, and he liked it when he came in and the computer picked it up straight away, and he'd look on the screen and it made me feel bad of how active he was.

P1 But he did do fantastic. So it sounds like you're saying that feedback.

P3 Yes. It was definitely the feedback made him want to do it more, yes.

P1 And did you notice a difference in that? So you say that he wanted to do more. Was there anything in particular?

P3 Well, I know he's always an active child. I think it really assured me how active he is when he's away from me, you know, so that was encouraging to me really, to see that, but sometimes with the Fitbit, because he'd come in and pore over his picture, then occasionally the downside I'd be shouting, "Come back and get your Fitbit", because I'd spotted it on the...., whereas with the Actigraph, it was always on the table and he could see it flashing, so you'd just think, "Oh, he's got to put that on".

P1 So you said that it was encouraging to you to see the feedback.

P3 Yes.

P1 Can you tell me a little bit more about that?

P3 Well, I just know that being active is really good for his chest, and I think so long as he's working that and he's managing to cough up, then it's all positive. You know, it's really good for him, so it's encouraging to see that he's not just going somewhere and sitting in his friend's house and doing nothing.

P1 OK. What do you think the negatives of wearing a physical activity monitor are?

P3 Well, he complained about the Actigraph. He said it was uncomfortable for him. With the Fitbit, he was good, but he was a bit wary of damaging it.

P1 Aaah!

P3 I think if it'd actually been his own, he wouldn't have been as wary, you know. I'd probably have to keep replacing it. But I think the fact that he knew it didn't belong to him was a little bit limiting.

P1 Aww, you were too good. So you say it was a little bit limiting. In what way?

P3 Just in a case of damaging it, you know, because I know he likes to climb trees, as much as I'm saying don't, I know he does, you know, so I know things like that he maybe thought twice of. But I'm sure he'd been active as much, finding something else to do.

P1 So thinking about P2, do you feel that the activity monitors encouraged him to be more active?

P3 With the Fitbit maybe, yes. Yes, just as we say, because we used to get the feedback.

P1 OK. And what is it about the Fitbit that P2 tried, you feel encouraged him to be more active?

P3 Just the fact that. as he said, it used to vibrate, and he'd see the lights going up and down. So it was more visual to him.

P1 And do you think it's feasible that your child, or rather P2, wearing a physical activity monitor might influence the amount or type of activity that he does?

P3 I don't think as much, because as I say, he is a really active child, so I don't think he'd notice it as much, just occasionally. I think when he felt the vibration he's go, "Ooh, I'm doing well." He's got the attention span of a fish, so he'd just be, "Wheee! Off to play a game."

P1 It's because he's so busy, isn't it, doing the activities?

P3 So I think it'd be a passing thought, and then he'd carry on doing what he's doing.

P1 Yes, so do you feel that's a reflection of P2's active lifestyle as it is?

P3 Yes, definitely.

P1 Thanks very much. Back to you, P2. Just a few more questions, if that's ok. The Actigraph shows that on average you did a hundred and thirty-two minutes, which is two hours twelve minutes, of moderate to vigorous activity each day. It's recommended that children and young people should do sixty minutes of moderate to vigorous activity each day to stay healthy. You did more than this. What do you think about that?

P2 Wow! I'm pretty happy, because it's more than I thought I could possibly do, but when I was wearing them I was just like really trying, so I'd do good.

P1 So you said wow, it makes you feel happy. Can you tell me a little bit more about that?

P2 Well, I really didn't actually think that I could do that good, but when I was wearing them, in the end I knew what it was for, so I started doing more stuff, like doing more walking and running and stuff.

P1 When you said there that you didn't think that you could do that well, can you tell me a bit more about that?

P2 Well, the only reason I didn't think I could do that well is because I usually lose

my breath and get a stitch, and then I have to take time out, but when I had that on I was just like taking it easy, and I'm like, "I'm doing too much", but I was always trying my best so I'd do it good.

P1 Sounds like you always try your best. OK. The Actigraph shows that you did the most moderate to vigorous activity on Wednesday. What do you think helped you to be more active on this day?

P2 Well, I just went up to the Astroturf to play footie with my mates, and then I went running.

P1 When you went running, where did you go running?

P2 Just all around Ware Wall.

P1 All around?

P2 Ware Wall.

P1 Oh, is that round by where you live?

P2 Yes, over there, kind of.

P1 And who were you with?

P2 Two of my friends that only live over the close.

P1 So you went running a little run. How long did you run for?

P2 I can't remember, because it was when I had the Fitbit.

P1 Ah, that's quite a while ago, isn’t it?

P2 Yes.

P1 Thanks for that. The Actigraph shows that on average you spent four hundred and seventy-eight minutes, which is seven hours fifty-eight minutes being sedentary, and sedentary means doing things while you're sitting still, like sitting and watching TV or being in school even. What do you think about that?

P2 Not much, really.

P1 Not much. Is there anything that springs to mind when I said that to you, that you're sedentary for seven hours and fifty-eight minutes?

P2 Well, I could have just done a bit of activities while I was just sitting down, like.

P1 So what do you think that you could have done while you were sitting down?

P2 Well, I don't know really. Just not really wasting as much time sitting down, and done more sports and whatever, rather than just sitting down and resting.

P1 And what sports would you do?

P2 Normal really, just go out and play football with my mates, and go running and then go out on my bike or whatever.

P1 OK. The Actigraph shows that you were more sedentary on Friday and Wednesday. What do you think about that?

P2 I don't know.

P1 OK. What was different about those days compared to Wednesday when you were most active?

P2 Well, all of them days I didn't do as much as Wednesday, because Wednesday was when I actually found out that's going to be measuring and telling me how good I've been doing with it all, and that's when I've done good.

P1 Ah, right. So having found out that you were going to get some feedback off the activities.

P2 Yes.

P1 What went through your mind when you found out that you were going to get some feedback on how much activity you did?

P2 Nothing really, just like to stay active.

P1 OK. Did you change the amount of activity that you normally do while you were wearing the physical activity monitors?

P2 No.

P1 No? OK. Can you tell me a bit more about that? You say no, you didn't really change how much activity you did.

P2 No.

P1 Can you tell me more about that?

P2 Well, I just didn't change the activities I was doing. I just stayed with the activities

I was doing, because the activities I was doing were the ones that were helping me get the high ones on the Fitbit.

P1 Did the physical activity monitors make a difference to the activities that you do?

P2 No.

P1 I think you've said that there when I said the question, but oh no, you've already answered that. Sorry. You stated that you disagreed that you tried new activities while you wore the activity monitors. Can you tell me a bit more about that?

P2 Well, I just didn't really change the activities I was doing, because all the ones I was doing I was just keeping to them ones.

P1 OK. Would there ever be a time when you would change the activities that you do?

P2 Not unless I was somewhere like big, and I had more space to do what I want, so not really.

P1 No? OK. Did you notice a change in how strenuous the activities you typically do are? Like how fast you're trying to run, or when you're playing footie with your mates?

P2 Well, I just try and run as fast as I can really, and then just try doing good stuff.

P1 Do you always try running as fast as you can?

P2 Well yes, because if I'm playing rugby or football or whatever, then I always have to run quite fast so I can get them.

P1 Yes, that's true. I suppose that's true. By the sounds of it, it sounds like you're really good at tackling.

P2 Yes.

P1 OK. Can you tell me about how you would use the Fitbits?

P2 Well, I just used it by every so often I would just tap it to see how good I'm doing and so on.

P1 OK. Was there like what settings on the Fitbit you used, or anything like that?

P2 No, not really.

P1 So apart from when you tapped it, did you used any other settings on the Fitbit?

P2 No, just kept tapping it to see how well I was doing and stuff. That's all.

P1 How about the dashboard? What settings did you use on the dashboard? The dashboard, you know the computer dashboard, so when you log on. What settings did you use on that?

P2 On what, the computer?

P1 Yes.

P2 Oh, right. Well, I didn't really look. It was just when I'd either come in and it got on the computer when my Mum was on it, and then I'd just see how well I was doing and stuff.

P1 And how often did you do that, do you think?

P2 Well, mostly every day when I'd come in from playing out I'd just always see how good I was doing.

P1 And when you say you'd see how good you were doing, what did that mean?

P2 Like see how much I've done in that day, and then it'd show you how many steps or whatever I've done.

P1 OK. And so you'd look at your steps. Was there anything else as well?

P2 No, not really.

P1 OK. And what was it that you liked most about the Fitbit settings? So like the step counter.

P2 Nothing really, because I didn't really look at it apart from daily when I came in, and then I'd just go up.

P1 OK. And what did you dislike about the Fitbit dashboard?

P2 Nothing, because I didn't really know much about it.

P1 And if there was some information then you would like to find out about it, what would that be?

P2 Just to see every day how good I've been able to stop.

P1 Yes. And so on the Fitbit you got your steps. What other information would you like to find out?

P2 What sports I've been doing, and how active I am and stuff.

P1 When you say how active you are, what type of information?

P2 Like just basically showing me not just the steps I've been doing, but showing me how well I've been doing on the activators and whatever I've been doing.

P1 Yes. OK. You mentioned on the feedback questionnaire that you disagreed that getting some information each day would encourage you to do more activity. Can you tell me a bit more about that?

P2 I can't think.

P1 You can't think. Has your brain seized?

P2 Yes. I can't think of anything.

P1 OK. So maybe thinking back to when you filled the feedback questionnaire, can you tell me what prompted you to circle or indicate that you disagreed that receiving information on how much activity you do would encourage you to do more activity? What was going through your mind when you filled that out?

P2 Nothing, really. I was just fine with it.

P1 You were fine with it. That's fine. Thank you. OK, so you said that you would not like to wear an Actigraph again, but that you didn't agree or disagree that you would like to wear a Fitbit again. Can you tell me a bit more about that?

P2 Well, I wouldn't like to wear an Actigraph again because it kept falling down, but the Fitbit I'd be perfectly fine wearing it, because it's just basically like wearing a watch, so I'd be fine with wearing one again.

P1 Yes. That's the end of the interview. Thank you very much for speaking to me today. I've really enjoyed hearing your thoughts about wearing physical activity monitors. Like I say, that's the end of the interview now. Is there anything more you'd like to add?

P2 No, not that I can think of.

P1 Something I've missed?

P2 No.

P1 How about you, Mum?

P3 No, no, I'm fine. That's interesting to hear the details of the Actigraph.

P1 Yes. I've got sheets here as well, so ... OK, I'll end the interview now. Thank you very much for your time and speaking with me today.

End of Interview

Phase 3 – P4

P1 Hi, P2. If you remember the last time we met, I spoke to you about then you're active and when you're not active, and then I asked you to try out two different activity monitors. One was the GenieActive and the other was a Fitbit. I'm here again today to ask you thirty short questions about what you thought about the devices you've tried out, like what you liked about them and what you disliked about them. There's no right or wrong answers, so don't worry. I'm interested in what you have to say, and may I ask for some examples if you can, because this'll help me to understand your experiences better. If you need to think for a little bit before answering a question that's ok. We won't try and answer for you. We'll just wait until you feel you feel ready to answer, or if we move past a question and then you remember something that you wanted to day, that's fine too. You can add things at the end. That's not to exclude you, Mum. There are some specific questions for you also, so watch out for these. It should take around forty minutes, depending on your answers, and I'll be recording our conversation on the Dictaphone just to help me remember everything we say. Also I'll be ticking off the questions as we go along just so I don't say the same question twice. If you or I happen to mention someone's name as we go through the question, or anything that might let someone else know who you are or other people, I'll be taking this out of the transcript of our conversation, and this is just a document where we write out our conversation. So if you're happy to begin? In this first section I'm going to ask you about the times and places that you wore the activity monitors we asked you to wear. So on the feedback questionnaire that you completed after wearing each physical activity monitor, you said that you strongly agreed that you enjoyed wearing the physical activity monitor. Can you tell me a bit more about that?

P2 It was fun to know that I could look back and see how much I was doing and my routinely day of what I would normally do.

P1 Yes. And which device was that for?

P2 It was for the Fitbit, so that I could look back on what I was doing, how many calories I’ve burned, how many steps I took, which was good.

I OK. And what did you like about wearing the physical activity monitors?

P2 That it didn't get in the way of me doing anything, or didn't prevent me from doing anything either, and as I said, it would allow me to see how much I was doing in a day.

P1 Yes. What did you dislike about the physical activity monitors?

P2 Nothing, really. There was nothing wrong with them.

P1 OK. And what physical activity monitor did you enjoy wearing most?

P2 I suppose it'd be the Fitbit, because that's the one that allowed me to look back at what I did.

P1 OK. So what is it about the Fitbit that you enjoyed, compared to the GenieActive, which was the watch one?

P2 The fact that I could look back on what I did, and I suppose it's a bit smaller than the GenieActives.

P1 Yes, you say it was a bit smaller than the GenieActive. Did that make a difference to you?

P2 It didn't make much of a difference, no, just I suppose if I was going to be picky that'd be one of the things.

P1 That's one of the things you'd say. OK. On the feedback questionnaire you said that you agreed that both devices were comfy to wear. What was it about the GenieActive and Fitbit that you felt made them comfy to wear?

P2 You could change how tight the straps were, so it was nice and snug, and they didn't rattle around at all. And it was a nice plastic, so it wasn't scratchy or anything.

P1 And was that for both devices, the nice plastic?

P2 Yes.

P1 OK. And was there anything that made them uncomfortable to wear?

P2 No.

P1 No? You said that you disagreed that the GenieActive and the Fitbit got in the way of what you were doing. Can you tell me a bit more about that?

P2 They didn't stop me from doing anything I would usually do. I did everything exactly the same. I couldn't not do anything I usually would.

P1 Ah. And when you say usually would, what would that be in reference to?

P2 Because I play table tennis, it wouldn't stop me from doing anything. I would put it on the hand I don't use, and it wouldn't affect anything.

P1 Yes. Ah, that's good. So it sounds like you're saying it wouldn't really get in your way of your activities that you did OK. What would you do to change the GenieActive and the Fitbit, if you could do anything to make it better?

P2 The only thing I would say is probably, if I was going to be fussy again, the GenieActive had some kind of way of telling you what you were doing, as it doesn't tell you what you've been doing sort of thing, where with the Fitbit, you can go on the internet and have a look at everything you've done.

P1 Yes. So you say that you'd prefer the GenieActive if it had something that sort of told you what you were doing. How would you like to see that information?

P2 Similar to the Fitbit, like go on the internet and it instantly synchs up like that.

P1 Yes. And what functions would you like to see on, if you could go on and see stuff?

P2 Like being able to see how many calories you've burnt. Be able to set goals and things like that.

P1 OK. And if you could set goals on it, what type of goals would you set?

P2 Amount of calories you burn, like set an amount that you would like to burn in a day or a week.

P1 Yes. Ah, thanks for that, P2. Did you ever have to be told to remove the monitor?

P2 No.

P1 No, ok. So next I'd like to talk about other people, like friends and family, and of how they may have influenced you wearing the physical activity monitor. Did you ever forget to put the physical activity monitors on?

P2 No.

P1 OK.

P3 You missed a day.

P2 Did I?

P3 Yes, you did.

P1 On the feedback questionnaire you said that you disagreed that you kept forgetting you put the physical activity monitor on. How was it for you remembering to wear the physical activity monitor each day?

P2 Well, I kept it next to where I sleep, so as soon as I woke up I'd put it on.

P1 Ah, yes. So it was like a prompt for you.

P2 Yes.

P1 You've kind of answered my next question actually, which was can you tell me

how you remembered to wear the monitor each day. So did you do that each time?

P2 Yes. With both of them, yes.

P1 Yes. Ah, thanks. You said that you agreed that other people noticed that you were wearing a physical activity monitor. What did people say to you?

P2 They just asked what it was, and I'd say that it was a monitor for a study, and that was about it.

P1 Yes. Did they ask you lots of questions?

P2 No. They didn't ask many questions at all.

P1 And when you said that to them, what did they say back to you?

P2 They just said ok, and that was that, really.

P1 And who was it who asked you about the devices that you were wearing?

P2 Just a few of my friends from school, really. That was about it.

P1 Yes. How did that make you feel, them asking you?

P2 It didn't really influence anything.

P1 Did it, you know, have an effect on how you were feeling at the time? Did you...?

P2 No, it didn't embarrass me or anything, if that's what...?

P1 Yes? Ah, that's brilliant. Thanks. Did you notice a change in the amount of the active activities you normally do as a family, while you were wearing the physical activity monitors?

P2 No, not really. It was life as usual. Nothing really changed.

P1 Life as usual. Aah, thanks, P2. how about your friends? Did how your friends are involved in you being active change?

P2 No. Nothing changed, no.

P1 No? OK. And on the feedback questionnaire you said that you disagreed that other people like friends and family kept asking how much activity you do while you were wearing the physical activity monitor. What do you think about that?

P2 I don't know.

P1 Sounds like nothing comes to mind.

P2 No, not really.

P1 No? Did you talk to anybody about what was happening, or anything like that? Did you sort of volunteer information, as it were?

P2 I mean, I told my family about when I was using the Fitbit and got to see how many calories I was burning and all that. I told them how much, and showed them how much I was doing and that.

P1 What did they say when you showed them?

P2 A bit surprised about how many steps I did in a day, walking back and to school and around, a bit surprising.

P1 Yes. Ah, thanks for that. OK, so thank you for your answers. I'm learning a lot about what wearing physical activity monitors was like for you. In this last section I'm going to explore how wearing a physical activity monitor may or may not have affected the activities you do. Do you think wearing a physical activity monitor encouraged you to be more active?

P2 No.

P1 No? How about other young people with CF? Do you think wearing the GenieActive and Fitbit would encourage them to be more active?

P2 It could do, to see what benefits it could do for them.

P1 Yes. And how do you think other young people, because you said that it could help them or encourage them to be more active, the GenieActive and the Fitbit, how do you think that would be for them?

P2 What do you mean?

P1 Oh right, sorry. I was just wondering if you could tell me a bit more the thoughts behind what you said. You know, you said it could be beneficial to them.

P2 Well, it could, say if someone didn't do an awful lot of exercise, and they use this and did everything they would normally do, and they saw that they weren't doing a lot, it may encourage them to do more activities, I suppose.

P1 Yes. So maybe seeing how much they don't do, and then seeing how much they do do. OK, thanks. How do you think using the GenieActive and the Fitbit could help you to be more active?

P2 I'm not sure. I don't know. I'm not sure.

P1 You're not sure. Oh no, that's absolutely fine. Can you tell me how you think using the GenieActive and the Fitbit was useful or not useful at encouraging physical activity?

P2 It didn't really encourage me to do any more, because I already do quite a bit, as I play table tennis most days in the week, so if was going to encourage me to do anything, I wouldn't have other days in the week to do anything.

P1 Yes.

P3 What it might be a good guide to, if you were unwell, comparing it to when you were well, to see whether it does actually affect how much you do, if you see what I mean, so you've got a longterm benefit from having it, so when you were well you had it on and you did like, say, two thousand steps in a day, but when you weren't well and you miss a week as in like table tennis, because you're not feeling too good, and then at the end of the week you probably feel like, "Oh, I don't feel good", and then you think, "Well, when I'm well I'd do that much, and when I'm not well I do this much", and you can weigh up, you know, the health side of it, I suppose, and then he might maybe think, "Well, if I did actually do that extra table tennis, would I feel better at the end of the week?" You know, so you could use it as a longterm guide, couldn't you, I suppose?

P1 Yes, that's a really good idea. What do you think about that, P2? You know, what your Mum said, using it as like a guide? Do you think that's something you'd do?

P2 I mean, yes, I'd wear it and then I'd see how much I do when I'm well, and how much I do when I'm not well, and then see how much it's affecting how much exercise I do.

P1 Yes. OK. Thanks. So when you say see how much it's affecting, do you mean how much being unwell affects your exercise?

P2 Yes.

P1 OK, thanks. What was it about the GenieActive and the Fitbit that you feel encouraged you to be more active?

P2 It didn't really encourage me to do much more exercise, because, as I've said, I already do quite a bit.

P1 Yes, that's true. OK. And what was it about the GenieActive and Fitbit you feel stopped you from being active?

P2 They didn't stop me from being active.

P1 OK.

P2 I did everything usual.

P1 Yes. Kept to your usual routine.

P2 Yes.

P1 OK. You can have a little rest now, P2. These are a few questions for you, Mum. What do you think the benefits of wearing a physical activity monitor are for P2?

P3 I think it's a joint benefit, because it'd benefit me, knowing how active he is, and that at certain times when he's not active because he's either unwell or he's off school. I mean, if he's off school, he's got a week off school, he's not going to be as active as if when he's in school, so it'd give us a guide to how much activity he should be doing, even when he's not in school.

P1 Yes.

P3 Because like half-terms, there's not table tennis on, and he doesn't walk back and to school, so it's a good idea, and you can see how much activity he probably needs to do to maintain a steady health, really.

P1 Yes. Ah, thanks for that. What do you think the negatives of wearing a physical activity monitor are for P2?

P3 If we're talking longterm, it's whether he would wear it all the time, and remembering to wear it all the time, I think.

P1 Yes.

P3 I think it's remembering to have it on him, yes.

P1 Do you think that was burdensome for P2?

P3 No, not for the short time. It was like, was it two weeks?

P1 Yes, it was a week.

P3 I don't think short term it was an effort for him to put it on. It wasn't a major issue, but if you're talking of putting it on for say six months, it's whether he would put it on for most part.

P1 Yes.

P3	I think that's the only thing, really. It's something he's got to maybe commit to if it's something he wants to do.

P1	Yes. And what do you think would stop P2 committing to that, if there was anything?

P3	I think it's more being forgetful than anything else, forgetting to put it on.

P1	OK. So thinking about P2, do you feel the activity monitors encouraged him to be more active?

P3	I don't think it's encouraged him more. I think it's made him realise how active he is, and opened his eyes a bit, and ours really, to how much he does do. Sometimes if you haven't got an active child, or whether you have got an active child, sometimes you don't realise how much you do or not do, until you put something like that on, really.

P1	And how did it feel, knowing how much P2 was doing?

P3	Well, I was made up for him really. I was proud that he was actually over-succeeding what they expected him to do, and he was made up with the Fitbit, because he kept getting the gold star at the end of the day. How fabulous! We'd look on the internet saying, what did it say? You're a...

P2	You're ace.

P3	You're ace, yes.

P1	That's good.

P3	I thought that was good.

P1	Yes. Aww! Do you think it's feasible that your child, or P2, wearing a physical activity monitor, might influence the amount or type of activity that he does?

P3	Yes, I think it is a good thing to have, a good tool to, yes. I do think it's really good.

P1	How much do you think it'd affect the type of activity that P2 does, or how much that he does?

P3	I think if he ever did give up table tennis, for instance, and he wasn't doing as much activity, it might encourage him then maybe to do something else, because he'd probably that, "Ooh, I'm not doing as much as I used to do", and he'd probably think about changing and doing a different sport or a different activity to maintain, because sometimes when, I know myself, that if you start playing a sport and you stop it, and you'll, "I can't be bothered doing that any more", and then you think, " Oh heck, I need to

do something now", by which time six months has gone. But no, I think it is a handy thing to have, yes, definitely.

P1 OK. Thanks very much. Back to you, P2. Just a few more questions now. The GenieActive shows that on average you did fifty-five minutes of moderate to vigorous activity each day. It's recommended that children and young people should do sixty minutes of moderate to vigorous activity each day to stay healthy. It is a little bit less than this. What do you think about that?

P2 I don't know.

P1 Surprised?

P2 Yes, I suppose. I don't know.

P1 You're not too sure? Ah, that's ok. So the GenieActive also shows that you did the most moderate to vigorous activity on Tuesday, when you did sixty-nine minutes. What do you think helped you to be more active on this day?

P2 Table tennis.

P1 Table tennis. Is that a day you do table tennis on? OK. And how do you think doing table tennis is different to other days for you, with regards to the moderate to vigorous activity?

P2 On the days that obviously I don't do table tennis, it's going to be different to the days when I do. I still walk to school, but it's not going to be as much as playing table tennis.

P1 Ah, ok. And what's the difference between walking to school and table tennis?

P2 Table tennis is like more ....

P3 Physical?

P2 Yes.

P1 More physical? OK. The GenieActive shows that on average you spend five hundred and thirty-six minutes, which is eight hours fifty-six minutes being sedentary, and this means doing things like when you're still like sitting and watching TV. What do you think about that?

P3 Eight hours a day?

P1 Yes.

P3 Does that include sleep?

P1 No, it would be when P2 was wearing the activity monitor.

P3 He's sat in school for six.

P2 Well, yes.

P1 Is there anything that goes through your head?

P2 That would be for school, wouldn't it?

P1 Yes.

P2 I can't really do anything about that, can I?

P1 I think your teachers might be a little bit upset if you start doing star jumps. So do you think school may have been am influence?

P2 Yes, that's probably with school.

P1 Aah, ok. And the GenieActive shows that you were more sedentary on Tuesday and Friday. What do you think about that?

P2 I've no idea.

P3 No idea? You don't do anything on Friday night, do you? Tuesday?

P2 Table tennis.

P3 On the odd occasion.

P2 And on every Tuesday.

P3 Friday. An odd Friday.

P2 I don't think I did Friday.

P3 On Tuesdays you play table tennis, don't you?

P2 Yes.

P1 OK, what was different about those days compared to Tuesday, when you were most active, which is a bit contradictory?

P3 Yes. That's what I was thinking. You sit still the longest on a Tuesday, but you

play table tennis for two and a half hours. Yes. Funny how it's worked out on the same day, isn't it?

P1 Let me just double check that I've got that right. So, yes. Most sedentary on a Tuesday and Friday, and your most active day was a Tuesday and a Thursday. Yes.

P3 I would have thought Saturday and Sunday would be his most sedentary days.

P2 I do a lot. Apparently being sat down all day is doing a lot.

P1 So is there any thoughts about that? You know, like...

P2 I'm surprised that I'm most active on Tuesday and most sedentary on a Tuesday.

P1 Yes. Surprised. OK.

P2 I even do practical PE on a Tuesday as well.

P1 Oh, right. Yes.

P3 So you have double.

P2 So that's another hour.

P3 Double, yes.

P1 Yes. OK. Don't think about it too much. It's just numbers. Did you change the amount of activity that you normally do while you were wearing the physical activity monitors?

P2 No. I did the exact same.

P1 You did the exact same. You disagreed that you tried new activities out while you wore the activity monitors. Can you tell me a bit more about that?

P2 Well, I wanted to try and keep the data that you got as accurate as possible to all I usually do, so...

P1 Yes.

P2 I didn't want to influence the data by doing more activity than I usually would do.

P1 Yes.

P2 To obviously keep it accurate.

P1 Yes. So you did what you normally do.

P2 Yes.

P1 Thank you. Did you notice a change in how strenuous the activities you typically do are, while you were wearing the monitors? Like how fast you run, or when you were playing table tennis and that?

P2 No.

P1 Can you tell me how you used the Fitbit? You know, like what settings on the Fitbit you used.

P2 I didn't change anything. All that was in was how I was given it.

P1 Yes.

P2 I didn't change anything.

P1 OK. So when you wore it of a day, did you do anything, or when you came home did you do anything, you know, look at anything?

P2 When I came home from school I would have a look at how many steps I did and how many calories I'd burned, and all that.

P1 Yes.

P2 I'd see how I did.

P1 So you looked at calories and steps. Was there anything else that you looked at?

P2 Every now and then I'd look at how much sleep I got.

P1 Yes, And what did that tell you?

P2 That apparently I sleep all through the night.

P1 That's reassuring, anyway.

P3 You sleep like a baby.

P1 Aaah! It's all that table tennis, that's what that is. OK, so you what is it that you liked most about looking at the sleep counter, looking at the steps and looking at the calories?

P2 I suppose it gives you something to be proud about when you get your most

amount of calories in a day, or you walk the most amount of steps you've ever done in a day. I suppose it's a good indicator of how much that you are actually getting, so then you can see if you need to try and get more sleep, or go to bed earlier or something like that.

P1 So how did you feel when you've seen all that information?

P2 I felt reassured that I was doing quite a bit of activity, rather than hardly any.

P1 Yes. And what did it mean to you seeing that you were doing lots of activity rather than hardly any? What did it mean to you, what was the significance to you of seeing that information?

P2 I suppose it taught me that what I was doing, I was doing it right. Like playing table tennis, I was actually doing the right amount of activity that I should be.

P1 Aaah, thanks for that, P2. And what did you dislike most about the Fitbit dashboard?

P2 Nothing, really.

P1 Nothing. OK. And you mentioned on the feedback questionnaire that you agreed that getting some information each day would encourage you to do more activity. Can you tell me a bit more about that?

P2 It would encourage me to continue on doing it, knowing that I'm doing something right, and that I'm helping myself by doing the amount of activity whereby it would tell me that I need to do more.

P1 Yes.

P2 That I shouldn't stop doing what I'm doing, and that I should carry on doing what I'm doing.

P1 OK. And what is it about getting some information that tells you how active you are each day, you feel would encourage you to be more active?

P2 I suppose knowing that I'm burning more calories, I'm doing a lot of steps, would make. I don't what I'm saying.

P1 You've mentioned calories quite a lot of times, you know, about burning calories and that. What does that mean to you when you've see that you've burnt lots of calories? What went through your head?

P2 What it means to me is that .....

P3 Even though you've got CF, you've got to maintain your weight, because you have been overweight in the past, haven't you?

P2 Yes.

P3 So where like with CF, isn't it, it's a struggle, or most patients struggle to put weight on, P2 has never had that problem, so he needs to keep an eye on how much he's eating, and how much he's burning, to maintain...

P2 So it helps me.

P3 So it helps you with your heart and your lungs, doesn't it? Because too much weight puts too much pressure on his lungs, which isn't good for his condition.

P1 Yes.

P3 So even though he has to keep active for his chest, he also has to keep active to maintain his weight, so he doesn't put weight on.

P1 Oh, yes. Ah, thanks for that. I know that was a difficult question, so thank you for that. OK. You said you would like to wear a Fitbit again, but you didn't agree or disagree that you would like to wear a GenieActive again. Can you tell me a bit more about that? Oh, have I done it the wrong way round? I do apologise.

P2 Yes, I agree for both.

P1 Oh yes, so you agree for both. I do apologise. It's just as well you noticed that. OK, so said that you'd like to wear both of these devices again. Can you tell me a bit more about that?

P2 I enjoyed wearing them, because I find out how much I'm doing, how much activities I'm doing and all that, so...

P1 Yes. So getting that information back. OK. And last question. What would you like to ask the clinicians? So if you could ask the clinicians in the CF team anything, what would you like to ask them?

P2 When's the next opportunity to wear one?

P1 The next opportunity to wear one? Aah, that's a brilliant answer. Thank you very much. And if you could choose one, which one would you wear?

P2 The Fitbit.

P1 The Fitbit.

P2 Because you get the feedback.

P1 Yes. Aah, thanks for that, P2. That's brilliant. Thank you very much for speaking with me today. I really enjoyed hearing what you thoughts about wearing the physical activity monitors. That's the end of the interview now. Is there anything more you'd like to add?

P2 No.

P1 No? Maybe something I've missed?

P2 No.

P1 How about you, Mum?

P3 No.

P1 Aah, that's brilliant.

P3 Do we get a final overview of the whole study?

P1 Yes, we will do. We'll do a report at the end for the hospital. We're hoping to publish in a medical journal, and also an evaluation report for participants and parents together as well.

P3 Oh, good. That'll be nice.

P1 OK, I'll end the interview now. Thank you very much for your time, and for speaking to me today.

End of Interview.

Phase 3 – P5

P1 So hi, P2. If you remember the last time we met, I spoke to you about when you were active and when you weren't active, or not active, and then I asked you to try out two different activity monitors, and one was the Actigraph, and that was the first one you wore, and that went round your waist, and then the other one was a pedometer, and that was the one which you couldn't really wear, because it clipped on. so I'm here today to ask you twenty-five short questions about what you thought about the devices you tried out, like what you liked and what you disliked about them. There's no right or wrong answers, so don't worry. I'm interested in what you have to say, and may I ask for some examples if you can, because this will help me to understand your experiences better. If you need to think for a little bit before answering a question that's ok. We won't try and answer for you. We'll just wait until you feel ready to answer. Or if we move past a question, and then you remember something that you wanted to say, that's fine too. You can add things at the end. that's not to exclude you, Mum. There are some specific questions for you also, so watch out for these. It should take probably about thirty minutes, depending on your answers, and I'll be recording the conversation on the Dictaphone, just to help me remember everything we say. Also I'll be ticking off the questions that I ask as we go along, just so I don't ask or say the same question twice. If you or I happen to mention someone's name as we go through the questions, or anything that might let someone else know who you are or other people, I'll be taking this out of the transcript of our conversation. And this is just a document where we write everything out. So if you're happy to begin? Is that ok?

P2 Yes.

P1 In this first section I'm going to ask you about the times and places that you wore the activity monitors we asked you to wear. I hear that you were unable to wear the pedometer, and that's ok, because I'm interested in hearing all about what it was like for you trying to use the devices, even when you couldn't wear them. OK. So to begin with, can you tell me about what stopped you from wearing the pedometer?

P2 We lost it for a couple of days, and then we found it.

P1 Yes?

P2 We found it. Because we always put stuff on the top of the fridge, and I looked up there to get some sweets, and I found it, but my Mum found it, though, and she left it there.

P1 OK. And did you ever try to put it on at all?

P2 Yes.

P1 How was it for you when you put it on?

P2 It was comfortable.

P1 Was it comfortable? OK. and when did you wear it?

P2 I wore it when I was playing out.

P1 When you were playing out. And when did you not wear it?

P2 I didn't wear it often after school.

P1 After school, yes. And how was that for you, when you did wear it and when you didn't wear it?

P2 Er....

P1 It's ok. Can you tell me anything about what you liked about it, or what you did not like about it?

P2 I liked that everyone kept asking about it, what it was.

P1 And what did they keep on asking you?

P2 They kept saying, "What's that?" And I said, "It's a monitor", and my friend said, "Why do you need a monitor?" and I said.....

P1 It's ok. It's fine. So P1, your friends asked you, and you said it was a monitor.

P2 Yes. It was a monitor, and it's for if I do loads of exercise, and I know what to say for it, but I just had doing exercise in my head.

P1 Ah, yes. And how was it for you when she asked about it?

P2 She said, "Oh, that's cool. Can I wear it?" and I was like, "No, I've got to keep it on".

P1 Aww, yes. So I know your Mum said that you did have some trouble wearing it, you know, when you had your school uniform and that on, and I was wondering what did you think it would be like to wear the pedometer, which was the one that clips on?

P2 I thought it was fine.

P1 You thought it was fine. That's absolutely great. Thank you very much, P2. I'm now going to talk about the Actigraph. And the Actigraph was the one that I first brought you. Remember when, ages and ages ago, you were in hospital, and it clipped round your waist. OK? So on the feedback questionnaire, which is this, that you completed, you said after wearing each physical activity monitor, you said that you disagreed that you enjoyed wearing the Actigraph. Can you tell me about that?

P2 It's because, do you know when you put it on the side, it hurts.

P1 Oh, did it hurt? What did it hurt?

P2 Do you know where you...?

P1 Oh, so like your hipbone? Is that what you mean?

P2 Yes. Every time I run.

P1 Oh dear. and was that all the time that it hurt?

P2 No, not all the time.

P1 Can you tell me about a time when it did hurt?

P2 It did hurt when I was doing some jogging, and I was starting to run a bit when I was doing jogging, and it kept hurting. I asked my Mum if I could take it off, and she said, "Later", because she wanted me to wear it, because I never wore it that much.

P1 OK. And what did you like about wearing the Actigraph?

P2 I loved it when everyone kept asking about it.

P1 And is that when H said to you that time?

P2 Yes. OK, so just to go back earlier, were you thinking when you answered that first question, was that to do with the Actigraph, that H asked about?

P2 Yes.

P1 Ah, I understand. So you liked it when people asked you about it. Can you tell me a bit more about that? What was it you liked people asking about?

P2 I liked when people asked, because I liked to explain to them.

P1 So you liked explaining to them. Ah, that's brilliant. and what did you dislike about wearing the Actigraph?

P2 When it kept hurting.

P1 When it kept hurting. It's not very nice if it hurts, is it, really? That's not very good. Thank you for trying it, anyway. So on the feedback questionnaire you said that you disagreed that the Actigraph was comfy to wear. What was it about the Actigraph you felt was comfy to wear? What was comfy about it?

P2 The feel of it when I was not running, and I kept forgetting about it, and I'd just get along with it.

P1 OK. And what was uncomfy about it?

P2 When it hurts.

P1 When it hurt your hips. Ah, right, I see. And you said that you didn't agree or disagree that the Actigraph got in the way of what you were doing. Can you tell me about that?

P2 It didn't?

P1 Yes, you said that you neither agree or disagree, so it was kind of in between, that the Actigraph got in the way of what you did, or what you were doing.

P2 Oh, I couldn't be able to do roll overs, because do you know when it digs in?

P1 Ah, yes. so you weren't able to do roll overs? Can you give me an example of when that happened?

P2 Do you know when you're starting, and then you land on your hip?

P1 Ah, so you kind of swish over. Is that what you mean?

P2 Yes.

P1 Oh dear. And where were you when you were doing roll overs?

P2 In mine, but not in the house. Like outside. And do you know the end house that you came to?

P1 Yes.

P2 That one. That side by the gates. There, on the Astroturf grass.

P1 Ah, right.

P2 And every time I kept doing roll overs it kept hurting me, and hurting.

P1 Oh dear. and what would you do to change the Actigraph, if you could do anything to make it better? So is there anything that you would change about it? No? OK. And what would you do to change the pedometer, so the one that you couldn't really wear? What would you do to make that better?

P2 Nothing.

P1 Nothing? That's great. and did you ever have to be told to remove the Actigraph, like take it off?

P2 No.

P1 No? OK. So next I'd like to talk about other people, like friends and family, and how they may have influenced you wearing the physical activity monitor. Did you ever forget to put the Actigraph on?

P2 Yes.

P1 Can you tell me a bit more about that?

P2 It was when I was playing out, because I'm rushing to play out.

P1 Because you couldn't wait to play out? That's fair enough, isn't it? I think that's far more fun. and on the feedback questionnaire you said that you disagreed that you can forget to put the Actigraph on. How was it for you remembering to put the Actigraph on each day? Can you remember how you remembered to put it on?

P2 I can't remember.

P1 That's ok. It was such a long time ago, wasn't it? And you said that you agreed that other people noticed you wearing an Actigraph. What did people say to you?

P2 People said, they said, "What is that?" And I said, "It's a monitor that shows your exercise."

P1 Very good answer. And what did you think about what they said?

P2 I thought it was good what they said.

P1 And how did it make you feel when people were asking you about it?

P2 Happy.

P1 Happy. Can you tell me a little bit more about what happy means to you, when people were asking you about it?

P2 Happy smile.

P1 Happy smile. Aah, you've got a lovely smile as well. That's a really good answer. Thank you very much. Did you notice a change in the amount of active activities you normally do as a family while you were wearing the activity monitors?

P2 No.

P1 And how about with your friends? Did how your friends are involved in you being active change?

P2 Yes.

P1 Yes? Can you tell me about that? So did the way you play with your friends change while you were wearing the activity monitor?

P2 No.

P1 No. OK. And on the feedback questionnaire you said that you disagreed that other people like friends and family kept asking how much activity you did while you were wearing the Actigraph. What do you think about that?

P2 I don't know.

P1 You don't know. That's fine, That's absolutely fine, P2. You're doing great. Thank you for your answers. I'm learning a lot about what wearing a physical activity monitor was like for you. In this last section I'm going to explore how wearing a physical activity monitor may or may not have affected the active activities that you do. Do you think that wearing a physical activity monitor encouraged you to be more active? Do you think that it encouraged you to do more, like play out more, or do more sport?

P2 I mean yes.

P1 Can you tell me how?

P2 How wearing the activity monitor encouraged you to do that?

P2 I've forgot.

P1 That's absolutely fine. so how about the other young people with CF? Do you think wearing an Actigraph and a pedometer would encourage them to be more active?

P2 Yes.

P1 Yes? and how do you think using the Actigraph and the pedometer could help you to be more active?

P2 It's good.

P1 Because it's good? and what's good about it?

P2 That I do more exercise than I used to do.

P1 And do you know why you do more exercise?

P2 Because I want to make Mummy happy.

P1 Aww, that's really nice. Aah, that's so sweet. That's a nice answer. And how about other children and young people with CF? How do you think wearing an Actigraph and pedometer could help them to be more active?

P2 Good.

P1 You think it'd be good for them? And what would be good for them about wearing the Actigraph?

P2 They could do more activities.

P1 Yes? And do you know how it would help them do more activities?

P2 I've forgotten.

P1 That's ok. You're doing really, really well, P2. And can you tell me how you think using the Actigraph was useful or not useful at encouraging physical activity?

P2 Useful.

P1 It was useful? What was it about the Actigraph that you think might have made it useful at encouraging you to be more active? So thinking about the Actigraph, that was the one that clipped on, how do you think it encouraged you to be more active?

P2 No.

P1 No, ok. And what was it about the Actigraph that you feel stopped you from being active?

P2 Never.

P1 Never? It never did. OK. So just a few questions for your Mum now, and then I'll come back to you. You're doing really well. What do you think the benefits of wearing a physical activity monitor are for P2?

P3 It doesn't stop her, and it doesn't gain anything for her.

P1 Yes. And can you tell me a little bit more about why that might be for P2?

P3 It's just because she does, like every day she's active, so she's not more active, or she's not less active. She's just...

P1 Just really active.

P3 Yes, she's just active all the time, so I don't think she'd benefit from it, because she is always active.

P1 Yes.

P3 Maybe if she was poorly, or she just stays in, but she's out 24/7, so I just don't think. It wouldn't impact on her life, I don't think.

P1 Yes. Ah, not for P2. And what do you think the negatives of wearing a physical activity monitor are for P2?

P3 I don't think there is, to be honest. It doesn't get in the way. It doesn't stop her from doing anything, so it doesn't really...

P1 Yes. Ah, that's great. and thinking about P2, do you feel the activity monitors encourage her to be more active?

P3 Not really. I think she'd forget, what was it, the first one?

P1 The Actigraph.

P3 The Actigraph. She didn't really know that was there, so she just played.

P1 Just carried on as normal.

P3 Yes. And she showed off in front of her mates.

P1 That's fair enough. Well, you've got to do something, haven't you? And do you think it's feasible that your child P2, that wearing a physical activity monitor might influence the type or amount of activity that they do?

P3 What do you mean, sorry?

P1 So do you think it's feasible that wearing a physical activity monitor, or P2 wearing a physical activity monitor, that it might influence how much activity or the type of activity that she does?

P3 See, P2, she's the same person, so she's not...Not really, no, to be honest. She is just the same.

P1 Sounds like your saying it wouldn't necessarily influence her.

P3 It just wouldn't influence her, no. She just does it anyway. She is an active person, so it wouldn't really make her, like she wouldn't go, "Oh, I need to put that on to make me go and do exercise", because she'd just go when she's outdoors anyway, but she does quite like swimming, so I just wouldn't think it would make an impact.

P1 Ah, thanks for that. That's brilliant. Ah, good shot, P2. I've got a few more questions to ask you if that's ok.

P2 Yes.

P1 OK. Did you change the amount of activity that you normally do while you were wearing the Actigraph? No? And can you tell me a bit more about that? You say it didn't change how much you did.

P2 I just stayed normal.

P1 You just stayed normal. So when you say you stayed normal, what does that mean?

P2 Like I do a lot of exercise and stuff.

P1 Ah, so you do a lot of exercise anyway.

P2 Yes.

P1 And on the feedback questionnaire you said that you disagreed that you tried new activities out while you wore the Actigraph. Can you tell me a bit more about that?

P2 I don't know. I've forgot.

P1 You've forgot. That 's absolutely fine, P2. and did you notice a change in how strenuous the activities you typically do are? No? And you mentioned on the feedback questionnaire that you agreed that getting some information each day would encourage you to be more active or do more activity. Can you tell me a bit more about that? So what is it? It's absolutely fine. That's absolutely fine, as long as you don't mind me chatting. So what was it about getting some information that tells you how active you are each day do you think would have encouraged you to be more active? So what information, if anyone could give you any information about how active you are, what information would you like to hear? So thinking about when you play out, when you do your things like swimming, and riding your bike, or anything like that, what information would you like to hear? What would you like somebody to tell you about when you do your activities?

P2 What do you do?

P1 What do you do? So thinking about when you go swimming, for example, what would you like somebody to tell you when you go swimming? You know, about how active you are, like how fast you go...

P2 You're very fast.

P1 So you're very fast. And how about when you're running? what would you like people to tell you?

P2 I'm very fast.

P1 OK. So thinking about all the different things that you do, it sounds like you're saying that maybe you'd like to find out how fast you go? Is that what you're trying to say?

P2 Yes.

P1 OK. And you said that you didn't agree or disagree that you would like to wear the Actigraph again. Can you tell me about that? So the Actigraph's the one that went round your waist, on like the elastic belt.

P2 Disagree. What's disagree?

P1 Disagree's when you kind of don't like it or something.

P2 Oh.

P1 Agree is kind of like saying yes, and disagree is kind of like saying no. So you kind of didn't say yes, or you didn't say no. You kind of said in between.

P2 Maybe.

P1 Yes, like a maybe. So you said like you'd maybe like to wear an Actigraph again. Can you tell me about that?

P2 Because it's nice.

P1 Because it's nice? And what's nice about it?

P2 That it helps me do a bit more exercise.

P1 And how does it help you do a bit more exercise?

P2 Because I want to make Mummy happy.

P1 Aaah!

P2 Again and again and again.

P1 Aaah! OK. And if you could ask the clinicians, so all the doctors, and the nurses, and the physios in the CF team at Alder Hey anything, what would you ask them? So you could ask them any question in the whole wide world.

P2 Am I good?

P1 Are you good? And what does good mean to you, P2?

P2 Active. Am I good at doing exercise?

P1 Are you good at doing exercise? Can I just go back to that very first question that I asked you? Because I heard that you were unable to wear the pedometer. So that was the clip-on one, so not the one on the belt, but the clip-on one, and that's ok, because I'm interested in hearing about all of it.

P2 The clip-on one I never really wore that one that much.

P1 OK. Can you tell me about that? Like what stopped you from wearing the pedometer?

P2 Because I just wanted to play out.

P1 Because you just wanted to play out. Well, that's fine. you're allowed to play out. And when did you not wear it?

P2 I didn't wear it today.

P1 Today. And when did you wear it?

P2 I wore it a couple of weeks ago, didn't I, Mum? The clip-on one, I tried it, remember? And it didn't stick.

P1 And what did it not stick on?

P2 On my knickers.

P1 Ah. Did it keep on falling off, or dropping down?

P2 Dropping down.

P1 OK. Well, P2, that's brilliant. That's all the questions that I've got to ask you, so thank you for speaking with me today. I've really enjoyed hearing what you thought about wearing the activity monitors. That's the end of the interview now. Is there anything more you'd like to add, like maybe something I've missed?

P2 No.

P1 No? How about you, Mum?

P3 No.

P1 No? OK, I'll end the interview now. Thank you for your time, and speaking with me today.

End of Interview

Phase 3 – P6

P1 So hi, P2. If you remember, the last time we met I spoke to you about when you're active and when you're not active, and then I asked you to trial two different activity monitors. One was a GenieActive, and the other was the Moves app. I'm here today to ask you thirty-one short questions about what you thought about the devices you tried out, like what you like about them and what you disliked about them. there's no right or wrong answers, so don't worry. I'm interested in what you have to say, and may I ask for some examples if you can, because this will help me to understand your experiences better. If you need to think for a little bit before answering a question that's ok. We won't try and answer for you. We'll just wait until you feel ready to answer, or if we move past a question, then you remember something that you wanted to say, that's fine too. You can add things at the end. That's not to exclude your Mum. There are some specific questions for her as well, so watch out for these. It should take around forty minutes, depending on your answers, and I'll be recording our conversation on the Dictaphone, just to help me remember everything we say. Also I'll be ticking off the questions I ask as I go along, just so I don't say the same thing twice. If you or I happen to mention someone's name as we go through the questions, or anything that might let someone else know who you are or other people, I will be taking this out of the transcript of our conversation, and this is just a document where we write out our conversation. So if you're happy to begin?

P2 Yes.

P1 OK. In this first section I'm going to ask you about the times and places that you wore the activity monitors, or used the activity monitors we asked you to wear. On the feedback questionnaire you completed after using each physical activity monitor, you said you strongly agreed that you enjoyed wearing the GenieActive. Can you tell me a bit more about that?

P2 Yes. It was comfy. It didn't get in the way, and it stayed nice and tight on my arm, so it weren't bobbing around getting in the way, and then there was a couple of times where I actually fell asleep wearing the watch, because I couldn't notice it.

P1 Oh right, ok. So what was it you liked about wearing the GenieActive watch?

P2 It was just the way it didn't get in the way. It was comfy.

P2 What did you dislike about wearing it?

P2 I didn't really dislike anything. It was just good. It was all right. It just stayed on nice.

P1 Ah, thanks. And for the Moves app, you said that you strongly agreed that you enjoyed using the Moves app. Can you tell me a bit more about that?

P2 Yes. I liked at the end of the day, looking at how many steps I'd got or where I'd ran, or things like that, going on my bike, because it was good to see how many steps I'd taken in a day.

P2 And what did you dislike about the app?

P2 There wasn't really anything I disliked about it really.

P1 Ah, thanks for that, P2. What physical activity monitor did you enjoy using the most?

P2 I don't know. I liked the Moves app.

P1 Yes.

P2 Because you could monitor everything yourself.

P1 Ah, yes. And when you say monitor everything yourself, can you tell me what you mean, please?

P2 With the watch you couldn't see what you'd done, but when you look at the phone you could see how many steps, where you'd ran, how far you went, how long you'd been walking for, and things like that.

P1 Ah, thanks for that. On the feedback questionnaire you said that you agreed that the GenieActive was comfy to wear. What was it about the GenieActive that you felt was comfy to wear?

P2 It was a nice, tight fit, and it wouldn't did into you anywhere, and it just stayed.

P1 OK. And what was it about the GenieActive that was not comfy to wear?

P2 I don't really think there was anything. I don't know.

P1 No? That's fine. And you strongly agreed that the Moves app was easy to use. Can you tell me a bit more about that?

P2 Yes. It was very simple, because as soon as you turned it on it shows you everything, and then just to see the other things all you have to do is tap the green circle with everything in, and it shows you everything.

P1 Ah, yes. So it was quite simple to use and all that.

P2 Yes.

P1 And what was not easy about using the Moves app?

P2 I don't think there was anything. I think it was very good.

P1 So do you think even someone like me could use it? I need things to be simple. You said that you strongly disagreed that the GenieActive got in the way of what you were doing. Can you tell me a bit more about that?

P2 Yes, it was just you put it on, and then you'd forget you was wearing it. Like I'd be playing football with it, it'd go under my sleeve, and then it wouldn't get caught on anything or get in the way.

P1 Ah, that's good. So it sounds like it wasn't like a distraction for you.

P2 Yes.

P1 And can you give me an example of when the monitor got in the way of what you were doing?

P2 I don't think it did really. No, I think it just, it was like a watch. It just didn't get in the way really.

P1 Ah, thanks. And what would you do to change the GenieActive, if you could do anything to make it better?

P3 That's ironic. I think once the weather's nice, where his friends'd ask him he was going to parties, and he'd feel a little bit embarrassed, wouldn't you, when people'd come up and say, What time is it?" and he'd have to say, "Oh, it's broke".

P1 Oh, yes.

P3 So if it had the time on, that'd probably be good.

P1 Yes. So your Mum there said that you felt a little bit embarrassed. Can you tell me a bit more about that?

P2 I don't know what she's going on about.

P3 Remember at parties. I remember you came home on two occasions and said it.

P1 So is there anything that you'd change to make it better, the GenieActive?

P2 It'd be good to see like what it was monitoring.

P1 Yes.

P2 And maybe like a little screen on the top, and you could see what you'd been doing.

P1 OK, so maybe some feedback that you can see there and then.

P2 Yes.

P1 Brilliant. And how about the Moves app? What would you do to change the Moves app, if you do anything to make it better?

P2 I think that was very good. It showed you everything you needed.

P1 Yes.

P2 I don't really think there was anything. It was very good.

P1 Ah, so there's nothing that you'd like to add to it, or change?

P2 No.

P1 Thanks. Did you ever have to be told to move the monitor or that?

P2 No.

P1 No? OK. Next I'd like to talk about other people like friends and family, and how they may have influenced you to wear the physical activity monitors, or use the physical activity monitor. Did you ever forget to put the physical activity monitor on?

P2 There was a couple of times in the morning where I'd wake up and I'd just forget to put it on, and my Mum'd send me back to my room to put it on and write down on the paper the time and everything.

P1 And how about the Moves app? How was it for you, you know, did you ever forget to sort of carry your phone on you, or anything like that?

P2 Not really. It was just when I'd be doing the sport I couldn't take my phone with me, because it'd be like swimming or football, something like that.

P1 Yes. OK. And on the feedback questionnaire you said that you strongly disagreed that you kept forgetting to put the GenieActive on. How was it for you remembering to wear the monitor each day?

P2 Well, because it was something I'd never really done, I'd get up in the morning, and when I didn't forget to put it on I'd be enthusiastic to put it on just for the research and for what is it, the stuff that you monitor.

P2 Oh yes, the physical activity.

P2 Yes.

P1 Ah, thanks for that. That's good. So it sounds like you were quite thoughtful about it.

P2 Yes.

P1 Thank you. And for the Moves app you didn't agree or disagree that you kept forgetting to look at the Moves app. How was it for you remembering to look at it?

P2 I'd look at it, but then sometimes I'd be talking to my mates, and where I'd look at it, I'd just like forget, and I'd just carry on talking, or sometimes I might leave my phone somewhere and not pick it up to look because I've forgotten, just things like that.

P1 Yes. Was it something that you thought about every day, you know?

P2 Yes, I liked to look at it to see how far I'd walked, and things like that.

P1 And was there a time of day that you particularly looked at it?

P2 I'd look at it after I'd maybe walked the dogs or gone out with my friends or something like that. I'd like to look at it to see what we've done.

P1 Yes. Ah, right. And can you tell me how you remembered to wear the GenieActive each day?

P2 Well, because I was enthusiastic about it, I'd have it next to me when I'd wake up, so I could just put it on, and if I did forget about it, my Mum would just tell me, so that's why I'd remember to put it back on.

P1 Ah, brilliant. Thanks for that. And how about the Moves app? Can you tell me how you remembered to look at the Moves app each day?

P2 Well, I'd be looking at my phone, and then I'd notice the little icon, like the GPS icon, and I'd be like, "Oh, that's for the Moves app. I'll have a look how I've gone on".

P1 Yes. So just like part and parcel of your normal day, it sounds like you're saying.

P2 Yes. And then sometimes I'd just remember to look at it, or I'd want to see how I'd done.

P1 OK. Oh, you mentioned actually, didn't you, after walking the dogs and things like that?

P2 Yes.

P1 You said that you disagreed that other people noticed you were wearing the physical activity monitor, and/or using the Moves app. What do you think about that?

P2 It was all right, because like my Mum said, I went to a few parties, and no one'd notice it was actually like a monitor. They'd just ask for the time, and I'd get my phone out, and I'd be like, "Why didn't you just use your watch?" I'd be like, "Because it's this monitor thing for the hospital", or like my Mum said, "It's broke".

P1 Yes. And how did it feel when people were asking about that?

P2 It was all right. It was mostly just my mates, so I could tell them.

P1 Yes. And what did you expect might happen while you were wearing and using the physical activity monitors?

P2 I didn't really know what was going to happen, because I'd never used it before.

P1 Yes. And did you notice a change in the amount of active activities you normally do as a family, while you were wearing the monitors or using the Moves app?

P2 I know my Mum took us to the baths a bit more.

P1 Yes.

P2 Like just for information on it to show you. I know I'd want to get our more, so you could have more to research, and things like that.

P1 Yes. Ah, thank you for thinking about that. And can you tell me how the active activities you do as a family changed while you were wearing the physical activity monitors?

P2 Well, usually we'll go to the baths like once a month, but then because it was in the two weeks' holidays as well, and there really weren't that much to do, and all my school sports and that weren't on, my Mum'd take us to the baths, and because I could keep it on, because it was waterproof as well, it was all right to swim in, and things like that.

P1 Yes. And what do you think about that? You know, that went to swimming rather than maybe doing your school things as it was the school holidays.

P2 It was fun, because it was better to be around everyone as well.

P1 Ah, yes. OK. And how about with your friends? Did how your friends are involved in you being active change?

P2 I know I'd be like I'd ask them do you want to have a game of footie or something where you could go out, because I'd want to try and do as much sport as possible for the monitor.

P1 Yes. So it sounds like you're saying because you were wearing the monitor you were a little bit conscious about being active.

P2 Yes.

P1 OK. And what do you thing about that? You know, how that made you feel, for example, what you liked about it, what you disliked about it?

P2 Nice. I found it all right. It didn't really affect, I don't know. I just wanted to get out more, so I didn't look like I was dead lazy or anything like that.

P1 On the feedback questionnaire you said that you agreed that other people like friends and family kept asking how much activity you do while you were using the Moves app, but that you didn't agree or disagree that other people like friends and family kept asking how much activity you do while you were wearing the GenieActive. What do you think about that?

P2 With the watch, because it didn't show how much I'd done, no one'd really ask about that. They'd just be like, "Let's see your phone". Because I know my cousin stayed over when I had the watch on, so he was like, "Let's see your phone. Let's see how much you've walked" or we went out or something like that.

P1 Ah yes. So more for the Moves app, because I suppose with the GenieActive you can't really tell anyone anything, can you? And what do you think it was about the Moves app compared to the GenieActive that prompted friends and family to ask how much activity you were doing?

P2 It was the fact that you could see on the Moves app how much activity you'd done, and because they're actually showing us where you've been as well, it'd say like I've walked to the shops, and then I've walked home, and then my Mum's driven me to somewhere, and it'd show you everywhere where you've gone, and because it'd show you, they were like, "Let's see".

P1 Yes, let's have a little nose.

P2 Yes.

P1 OK. Thank you for your answers. I'm learning a lot about what wearing physical activity monitors was like for you. In this last section I'm going to explore how wearing a physical activity monitor may or may not have affected the activities you do. Do you think that wearing a physical activity monitor, or using the physical activity monitor, encouraged you to be more active?

P2 Yes, because I wanted it to show up that I'd been doing more activity as well, so you could see, for the research.

P1 Yes. Ah, thanks. and how about other young people with CF? Do you think wearing the GenieActive and using the Moves app would encourage them to be more active?

P2 Yes. If they were already active, and they put it on, I think it'd encourage them as well, to be more active like I was, so it'd show up for the research, to show how the monitor was.

P1 Yes. And how do you think using the GenieActive and Moves app could help you to be more active?

P2 Well, I'd go for a jog, I'd jog to football, and I'd like to see how far I'd jogged, and how many steps it'd taken me to get to football, and things like that.

P1 Ah, yes. So like the feedback side of things.

P2 Yes.

P1 OK. Can you tell me how you think using the GenieActive and Moves app was useful or not useful at encouraging physical activity?

P2 I find it was useful because the information. You'd be happy if you'd seen that you'd done a lot, because I know there was some days where it'd just be like you've done like four hundred steps, and you'd be like, "Oh, it's not really that good. Usually I'm doing like twelve thousand", and things like that.

P1 Aah. And what would you do when you found out that you'd maybe done four hundred steps?

P2 I'd be a bit like I've been a bit lazy today. I haven't really done much.

P1 Yes. So a bit lazy. So that's what'd go through your mind.

P2 Yes.

P1 OK. And what was it about the GenieActive and/or the Moves app that you felt stopped you from being active?

P2 I don't really think there was anything that stopped me from being active, because if anything it encouraged me to see how I'd done.

P1 Yes. OK. So this is just a question for you if that's ok, P3.

P3 OK.

P1 What do you think the benefits of wearing a physical activity monitor are for P2?

P3 I think, because of the time that it was given to him, it was given to him in the half=term, I think with it being half-term, his activity would have decreased, but it did encourage him to be a bit more active, and I think because he likes to help people with research, I think that also encouraged him to get up and go, simply because he wanted to see the outcome, the results. I don't really think there would have been anything else that would have encouraged you to get up and get out, would it? But using the activity equipment did encourage him to get out and about.

P1 Thanks. And what do you think the negatives of wearing a physical activity monitor, or using a physical activity monitor are for P2?

P3 I don't think there were any negatives, to be honest with you. It was all quite simple, you know, the devices. You had one on your wrist, and you can use that any time you want, the advantage that it was waterproof and quite resistant to any damage. I think that made it a lot easier for somebody of P2's age to use, and with also having that Moves app, P2's quite technical with his phone and everything. He likes to have his phone on him all the time, so I think that also helped as well. I think using the two of them together as a combination if kids are of an age where INAUDIBLE.

P1 Yes. And thinking about P2, do you feel that the activity monitors encouraged him to be more active?

P3 I don't know. I think P2's generally quite active anyway. I think if it had been J's as normal, J's normal school term, there may have been a similar level of activity. I don't think if it was given to him in J's school term, I don't think it would have altered his activity. I don't think it would have increased any level of activity. If you're looking for an answer to would it encourage others to be a bit more active, I'd be a little bit optimistic. I think it's a case of whether the child's already active, and if they want to improve, to people that are active. Because I think that's what it was with P2. He didn't want to show a lower level of activity in comparison to what he normally does. So I think it was good for P2 to have on a trial basis, but I think over a long term, I don't think it'd encourage him to do any more activity than normal.

P1 Yes.

P3 I think it was just a good time to catch him.

P1 Yes.

P3 Because it was half-term, and his level of activity did decrease because he was normally walking to school and walking back, and I think he just likes to show that he is active. I don't know how that'd affect anybody else in the long term.

P1 Brilliant. Ah, thanks for that. And do you think it's feasible for P2, wearing a physical activity monitor might influence the amount or type of activity that he does?

P3 In the long term?

P1 Yes.

P3 In the long term I couldn't see it increasing his activity. Because he is generally an active kid, I think he'd just maintain the level of activity, and I think because of the age that he is, although he's happy to impress at the moment, I think he could get to a point where it's, "Oh, I'm not doing this, and I'm not doing that". I think for a certain level of time he'd like to conform to prove high activity, but I don't think it'd encourage him in the longterm.

P1 I suppose, like you're saying, of obviously, you know, the conformity. I suppose that's a reflection of just being that age, isn't it?

P3 Yes. Well, I think because he's in that teenage stage as well now, it is an awkward age, you know. He's going through a stage where he can't be bothered doing things, and yet at other times he's got all this energy, and he does want to get out and burn some energy off, but I think on a

longterm basis, I don't think it'd encourage him to do any more than what he already does, or what he's used to.

P1 Yes. Ah, thanks for that. Back to you, P2, if that's ok. So the GenieActive shows that on average you did two hundred and sixty-three minutes, which is four hours twenty-three minutes, of moderate to vigorous activity each day. It's recommended that children and young people should do sixty minutes of moderate to vigorous activity each day to stay healthy. You did more than this. What do you think about that?

P2 I'm kind of impressed with myself for doing four hours a day, and yes, I'm pretty impressed with myself.

P1 Yes. Is that something that you expected?

P2 No.

P1 What were you thinking? If you had to put a number on it, what number were you thinking?

P2 I'd say about an hour.

P1 What makes you say that?

P2 Because it was the holidays I didn't feel I had done as much as what I would do in school.

P1 Yes. Thanks for that. And the GenieActive shows that you did the most moderate to vigorous activity on Saturday. What do you think helped you to be more active on this day?

P2 I think it was because, yes, there was a party.

P1 There was a party? Aww, what type of party was it?

P2 My mate's girlfriend had a party.

P1 Ah, yes.

P2 Like loads of people went, and it was like, there was a DJ and that, you know.

P3 He was dancing till two in the morning.

P1 Oh, were you? Aww, dancing till late in the morning. So do you think that may have contributed to the activity?

P2 Yes.

P1 And the GenieActive shows that on average you spent three hundred and fifty-four minutes, which is five hours fifty-four minutes, being sedentary, which means doing things while you're still, like sitting and watching TV. What do you think about that?

P2 Is that a day?

P1 That was just on average, yes.

P2 That's actually, I don't know. It was probably because I was taking it off late at night as well. I was taking it off about eleven o'clock, so I was probably, I don't know.

P3 I think if you look at the positive of that, you exercise almost as much as what you did rest.

P1 And I think as well, you've got to look at, especially during the holidays, you get up later, which is understandable, because you're up at the crack of dawn otherwise, and like you say, you go to bed a little bit later, so you can't really be out on the streets late at night, so you almost have no choice but to be inside, do you?

P2 And then there's a few times where I fell asleep, so that would have added to the average as well.

P1 OK, thanks for that. And the GenieActive shows that you were more sedentary on a Sunday and Monday. What do you think about that?

P2 Sunday and Monday?

P1 Sunday and Monday. And what was different about those days compared to Saturday, when you were most active?

P2 Usually it's on a Saturday that I'll do something with my mates, and I think Sunday would have been because I had a banging headache.

P1 Aah, yes.

P2 And I couldn't hear nothing or my throat was hurting really bad, so that would have been Sunday.

P1 Yes. After the party?

P2 Yes. And Monday. Was R, was he there on Monday? It might have been because my cousin was there.

P1 And did you change the amount of activity that you normally do while you were wearing the physical activity monitors?

P2 Yes, because...

P3 There was a lot less activity than normal, because he'd normally walk to school, does PE lessons, walk home from school, come home, run to football, play football, and then three times a week to Thai boxing as well, so I think he was conscious that he wasn't doing any of that, plus walking the dogs, wasn't he?

P1 Yes. And I was going to say can you tell me about the difference wearing a physical activity monitor made to you?

P2 I was encouraged to do more activity so there's more research for how the monitor monitors.

P1 And what was going through your mind, you know, your Mum mentioned that you were on holiday? Is that something that you were thinking about?

P2 Yes, because, like my Mum said, I'd usually do a lot like to routine. Because it was the holidays and everything, it just got thrown out of the window. I didn't want it to look like I just do nothing.

P1 Yes. Oh no, you're on holiday. You're entitled to a break. And on the feedback questionnaire you said that you didn't agree or disagree that you tried new activities out while you wore the GenieActive and used the Moves app. Can you tell me a bit more about that?

P2 Yes, because I just did the usual stuff I've done, as well as I went to the baths and things like that, because there was nothing that I could really introduce.

P1 Yes.

P2 Because I do all the sports that are available to me, because I can't go and do like sky-diving or ....

P1 Aww. You could try, but can you imagine, trying to fit that in with your homework?

P2 Yes. I was actually meant to be going for my birthday.

P1 Oh, really?

P2 But then they put an age limit on it.

P1 Aww! Oh, well maybe in a few more years' time.

P2 Yes.

P1 Nearly close to the end now. Did you notice a change in how strenuous the activities you typically do are, like how fast you run, or when you were doing your Thai boxing?

P2 I felt like I was just going at what I usually go, but it was around that time as well that, because I've started doing like even more activity, I felt like I had more energy to do more, so that might have been I used more energy in the sports.

P1 Yes. And can you tell me about how you used the Moves app?

P2 Well, what I'd do, say I'd go out, and once I came back in and I'd go to sit down or something, I'd look at my phone to see what I'd done, and then I'd have a look where I'd been as well, so it was fun to look where I'd been.

P1 Ah yes, course. Ah, go on. Sorry, you were saying. So I'd asked you can you tell me how you used the Moves app.

P2 Yes. So I'd come in from doing what I was doing, and then I was about to sit down or I just came in and didn't have anything else to do, I'd look at my phone to see where I'd been and how long I'd been walking, and the distance, and how many steps I'd taken.

P1 Yes. And so apart from the step counter, did you use any of the other settings on the Moves app?

P2 Yes, I used the bicycle one and the running one.

P1 Yes. And what did you like most about the bicycle and the running?

P2 I liked how it showed you, I think it was the average speed, I think it was, and the distance you'd gone.

P1 Ah yes. And what did you dislike about the Moves app?

P2 I didn't really dislike anything, because it was so simple, and it showed you what you wanted to see.

P1 Yes.

P2 It was good to see how you'd done.

P1 Yes. And how did you find having to have the phone on you when you were being active, you know, so it would pick up how much activity you did?

P2 Well, usually I carry my phone everywhere anyway, so it didn't really affect me.

P1 Ah, that's good to hear. Yes, that's good to hear. Did you ever have to not have your phone on you when you were active?

P2 There was maybe a couple of times where I'd leave my phone in the house by accident, where we'd go to the shops or something.

P1 Yes.

P2 There was times like that.

P1 OK. And apart from that, sorry, I was going to say what was it you were doing, but you've just told me, haven't you? You were going to the shops. Just ignore me. And you mentioned on the feedback questionnaire that you strongly agreed that getting some information each day would encourage you to do more activity. Can you tell me a bit more about that?

P2 Yes. To see how much activity you'd done in a day, like I said, say you do like four hundred steps, you'd be like encouraged the next day to do even more, to show to yourself that you're not being lazy all the time, and I know there was a few times where I'd get like ten thousand, and the next day I'd be like, "Well, I'll try and get like twelve thousand", to see how many I could get.

P1 Yes. So like a little competition with yourself. Brilliant.

P2 Yes.

P1 And what was it about getting some information that tells you how active you are each day you feel would encourage you to be more active?

P2	I felt, because it was showing me how much I was doing, it was making me feel better with myself, so then it'd encourage me to do even more, and to keep it up.

P1	So you say it made you feel a bit better in yourself. What type of things did it make you feel?

P2	Yes. It made me know that I'm not being lazy in the holidays, just like all my mates.

P1	Ah, thanks. Nearly close to the end now. You didn't agree or disagree that you would like to wear a GenieActive again, but you said that you would like to use the Moves app again. Can you tell me a bit more about that?

P2	I must have left the use it again, because I would use it again.

P1	Would you? Ah, right, yes.

P2	Yes. I would use it again, and the Moves app, I still use it.

P1	Yes. And which one, if you were given the choice of the two, which one would you choose first to use?

P2	The Moves app.

P1	Yes. OK. and if you could ask the clinicians in the CF team at Alder Hey anything, what would you ask them?

P2	I don't know.

P1	Anything in the world you could ask them, absolutely anything.

P2	I don't think there is anything, really.

P1	No? Ah, that's fine, then. Thank you very much for speaking with me today. I've really enjoyed hearing about what you thought about wearing the physical activity monitors. That's the end of the interview now. Is there anything more you'd like to add?

P2	No. That's it, then.

P1	Maybe anything I've missed?

P2	No.

P1	No? OK, then. I'll end the interview now. Thank you very much for speaking with me today.

End of Interview

Phase 3 – P7

P1 So hi, P2. If you remember, the last time we met I spoke to you about when you're active and when you're not active, and then I asked you to try out two different activity monitors. One was a GenieActive, and the other one was a Nike Fitbit. I'm here again to ask you thirty short questions about what you thought about the devices you tried out, like what you liked about them and what you disliked about them. There's no right or wrong answers, so don't worry. I'm interested in what you have to say, and may I ask for examples if you can, because this'll help me to understand your experiences better. If you need to think for a little bit before answering the question, that's ok. We won't try and answer for you. We'll just wait until you feel ready to answer, or we move past a question and then you remember something that you wanted to say, that's fine too. You can add things at the end. That's not to exclude you, Mum. There are some specific questions in there for you also, so watch out for these. It should take around forty minutes, depending on your answers, and I'll be recording our conversation on the Dictaphone, and just to help me remember everything we say. Also I'll be ticking off the questions that I ask as we go along, just so I don't say the same thing twice. If you or I happen to mention someone else's name as we go through the questions, or anything that might let someone else know who you or other people are, I'll be taking this out of the transcript of our conversation, and this is just a document where we write out our conversation. So if you're happy to begin? OK. So in this first section I'm going to ask you about the times and places that you wore the activity monitors we asked you to wear. So on the feedback questionnaire you said that you completed after wearing each physical activity monitor, you said that you agreed that you enjoyed wearing the physical activity monitors. Can you tell me a bit more about that? So what did you like about wearing the physical activity monitors?

P2 With the GenieActive, it looked more like a watch, so people didn't say something, where with the Fitbit, you could put it, because it was like slim and that, you could put it on your ankle, it would go round your sock, it would go inside your sock, so you could still do like basketball and other sports with that on.

P1 OK. And what did you dislike about wearing the activity monitors?

P2 The GenieActive was a bit too big, so it was hard to ignore that it was there, whereas with the Fitbit you could just ignore it was there. The Fitbit you couldn't exactly tell whether it was on or it was off, because you have to double tap it, tap it once or something. It's just a bit complicated.

P1 Ah, right. So that was something that you disliked about it? OK. And what physical activity monitor did you enjoy wearing most?

P2 The Fitbit.

P1 The Fitbit. And what was it about the Fitbit that you enjoyed, compared to the GenieActive?

P2 Because it was smaller, like relevant to wear for sport and that, because you

could just hide it.

P1 Ah, right. So when you say relevant, could you tell me a little bit more about that?

P2 It was more, I can't think of the word. So I think it was better to wear for sport, because it was smaller and more....

P1 Ah, right. And that was something that you could wear round your ankle, you said earlier.

P2 Yes.

P1 OK. Thanks for explaining that to me. On the feedback questionnaire you said that you agreed that the Fitbit was comfy to wear, and you didn't agree or disagree that the GenieActive was comfy to wear. What was it about the Fitbit and the GenieActive that you felt made it comfy to wear?

P2 The Fitbit was more comfortable material, because it was like a rubbery plastic, and it was smaller, so it was easier to wear on your wrist or your ankle, and you'd just forget it was there, and then you don't have to take it off and take it on when you go to bed and when you go out, whereas the GenieActive was bigger, and it looked more like a big old-fashioned clunky watch. Well, not old-fashioned, but like a big thingy watch, and you'd have to keep taking it off and on, so it'd be a bit complicated. You'd have to remember to take it off and remember to put it back on.

P1 OK. Thanks for that. And what was it about the Fitbit and GenieActive that made it uncomfy to wear?

P2 The GenieActive was quite clunky.

P1 Or chunky.

P2 Yes, chunky. That's the word. And it was like uncomfortable to wear, because like I say, if you were running, you couldn't exactly wear it, whereas the Fitbit, there wasn't really much uncomfortable about the Fitbit. Apart from sometimes it might dig in, so it was hard to put the holes into the thing.

P1 Oh, on the clasp? Yes. So you found that a little bit difficult.

P2 Yes. You'd have to try and dig it in so it would hurt to put it on.

P1 Aah, ok. Thanks for explaining that to me. You said that you disagreed that the Fitbit got in the way of what you were doing., whereas you didn't agree or disagree that the GenieActive got in the way of what you were doing. Can you tell me a bit more about that?

P2 The Fitbit never got in the way, because you could just hide it in your sock, or because it was quite small, again you could forget that you had it on, but the GenieActive again you couldn't put it on your ankle. You had to put it on your wrist, so you couldn't wear it, so it would get in the way, because I wasn't able to wear it.

P1 Yes. Aah, thanks for that. And can you give me an example of when the monitor got in the way of what you were doing?

P2 I couldn't wear it for basketball. It was seen as like a jewellery, so like I say if you were marking someone you might hurt them, because it's quite big.

P1 Ah. And what one was that, that you couldn't wear?

P2 The GenieActive.

P1 OK. And where were you when you were doing basketball?

P2 At the sports centre across the road.

P1 OK. Thanks for that. What would you do to change the GenieActive and the Fitbit, if you could do anything to make them better?

P2 Make the GenieActive be a bit smaller, and maybe have a time on it so people could go and just a watch, instead of having to explain to people that it's to help, it's to show about physical activity and all that. And the Fitbit, maybe have it in more neutral colours, so it's harder to notice, so you don't have to explain yourself, say grey or something, because if it's bright pink, they'd go, "Oh, what's that?"

P1 Oh, yes. I see what you're saying there. So some colours and some features that make it not as noticeable to other people.

P2 Yes.

P1 OK. Did you ever have to be told to remove the monitor?

P2 I didn't have to for the Fitbit, because you could hide it in your sock, or in gymnastics they were fine about it, because the material, it could just stay in my ankle, but I had to take it off, the GenieActive, for gymnastics and basketball, because it was quite big. It was on my wrist, so you couldn't wear it for activities. You might hit someone else.

P1 And who told you to remove the device?

P2 The coach and instructor.

P1 And what reasons did they give you for having to remove the device?

P2 Because you might hurt someone else, so you actually might hurt someone else, and you might hurt yourself in gymnastics.

P1 Ah, so it's quite important that you don't hurt yourself or anyone else, isn't it? Thanks for that. Next I'd like to talk about other people like friends and family, and how they may have influenced you to wear the activity monitors. Did you ever forget to put the physical activity monitor on?

P2 I didn't forget with the Fitbit, but I could just keep it on all the time. I’d take it off for the shower, so I think I forgot once. I think I forgot a few times with the GenieActive, because you have to take it off at night, so I might forget to put it on in the morning after an activity.

P1 Yes. And on the feedback questionnaire you said that you agreed that you kept forgetting to put the GenieActive on, but that you didn't agree or disagree that you kept forgetting to put the Fitbit on. How was it for you, remembering to put the monitor on each day?

P2 Wit the Fitbit, again you could just keep it on, so it's easier just to leave it, and then you take it off and put it on in the shower, but the GenieActive was like you had to take it off at night, so then you'd have to put it back on in the morning, and sometimes I'd forget, and say if I'd gone out late I might be rushing around, so then I'd forget to put it on for the day.

P1 Yes. Can you think of an example, or give me an example of a day when that may have happened?

P2 I think I forgot to put it on on Sunday, because again, I think I got up late and had to get ready quick, and I think I just forgot to put it on.

P1 That's fair enough. Thanks. Can you tell me how you remembered to wear the monitor each day?

P2 I put the GenieActive next to my bed, so that when I got up I could see it, but again, the Fitbit I'd just keep on.

P1 Ah, so you didn't need to remember to put it on. Ah, thanks for that. You said that you agreed that other people noticed that you were wearing a physical activity monitor. What did people say to you?

P2 They just asked what it was and what it did, and someone asked what the time was with the GenieActive, and I had to say, "Oh no, it's not a watch, it's a blah, blah, blah", but the thing with it, because again with the Fitbit, it was more the bright colours, so people would be like "What's that on your arm? You're not allowed to wear bracelets for gymnastics", but then you'd have to explain, whereas again with the GenieActive you

had to explain that it's not a watch, it's a physical activity monitor.

P1 How did it make you feel when people asked you about it?

P2 I didn't mind much, because they're just asking.

P1 Ah, so it wasn't so bad?

P2 No.

P1 Ah, thanks for that. Did you notice a change in the amount of active activities you normally do as a family while you were wearing the physical activity monitors?

P2 Not as much with the GenieActive, because you couldn't tell how much you were doing and stuff like that, but with the Fitbit you could see on the computer your stuff that you've done, so how it is I'd go on the trampoline more, or I'd do stuff. I think I did more on the treadmill.

P1 Ah, so can you tell me a bit more about that? You said that you could see on the computer on the dashboard how much activity you did.

P2 It does like a graph and it also tells you how much you've done that day, and how much you've got to do to beat the day before and stuff.

P1 And what went through your mind when you read that to sort of...?

P2 Well, I'm quite competitive, so I had to beat it.

P1 And what did you like about that?

P2 It can give you a goal to beat it, so that you could do better than you did the day before.

P1 And what did you dislike about it?

P2 At first it was a bit complicated to set up. It wasn't set up properly, so I had a day's thing that I did, and that wasn't recorded. And the sleep monitor thing, where you double tapped it and then double tapped it didn't work as well, and it kind of confuses me with that. It's like I think the website had a bit too much on it, because it said too much.

P1 Ah, so was it the too much information that confused you?

P2 Yes. Sometimes I had to double tap it, and then tap it once, and then tap it again or something, so it was a bit, all the tapping, it was a bit confusing.

P1 Yes. What would you have preferred rather than the tapping?

P2 Maybe like a little not touch screen, but like a few buttons, and maybe a little screen, so that you could see what you were doing instead of just pressing it blindly, and then thinking, "Oh, I might just press it again. Do I need to press it one more time?"

P1 Oh, I see. So something a little bit clearer that you could see here and there?

P2 Yes.

P1 Thanks. How about your friends? Did how your friends are involved in you being active change?

P2 Not really, because the stuff that I go to is more of a club thing, so they'd have to join in, and they'd have to keep going with it, so it'd be a big thing just to start like for a few weeks and then stop after I'd done it, because they couldn't exactly stop, because all the payments and stuff.

P1 Ah, right. So just so I can understand, did the way that you're active with your friends change, or did that sty the same?

P2 It just stayed the same.

P1 Ah, I see. Brilliant. And on the feedback questionnaire you said that you disagreed that other people like friends or family kept asking how much activity you did while you were wearing the physical activity monitor. What do you think about that?

P2 It's good, because I don't like too much attention being drawn to what I do and why I do it and stuff like that, so they kind of just let me get on with it instead of talking about it all the time.

P1 Yes. Thanks. Thank you for your answers. I'm learning a lot about wearing a physical activity monitor was like for you. In this last section I'm going to explore how wearing the physical activity may or may not have affected the activities you do. Do you think that wearing a physical activity monitor encouraged you to be more active?

P2 I think it did, because with the Fitbit you could see what you were doing, so you'd see what you've done and stuff like that, whereas with the GenieActive you had to do it like blind, so you couldn't tell whether you've beaten the day before or how many steps you've done and stuff like that, so with the Genie, not really, but then with the Fitbit, yes.

P1 Yes. How about other young people with CF? Do you think wearing the Fitbit and/or the GenieActive would encourage them to be more active?

P2 I think the Fitbit would, because if they were competitive they'd be able to see how much they're doing, and if they weren't they might realise they're not doing enough

for that week or for that day, so they might take it upon themselves to start being more active and stuff, but I think the GenieActive would be a bit complicated, because you have to remember to put it on in the morning and take it off, and stuff like that.

P1 Yes. Ah, thanks. And how do you think using the Fitbit and GenieActive could help you to be more active?

P2 I don't think I could be any more active than I was, because you have to have days off, but I think the Fitbit would, say if I went on the trampoline more, or I decided to start running or something, that could, because then you could see how many steps and stuff, and if I had space I would, but I don't think the GenieActive would help that, because it's too complicated, not complicated, but you can't see what you've done, whereas with the Fitbit you can.

P1 So maybe if you had space, maybe the Fitbit would, but the GenieActive wouldn't, because you don't get any feedback. Brilliant. And can you tell me how you think using the GenieActive and Fitbit was useful or not useful at encouraging physical activity?

P2 Yes, with the Fitbit, you could see what you were doing and how well you were doing it, and whether you should be doing more, but again, with the GenieActive, you couldn't see what you were doing, and it might get in the way of things, so you might not want to go to things that you were doing, because you'd probably have to take it off, and then people would ask what it was and stuff.

P1 Yes. And how about the Fitbit? How do you feel that encouraged you to be active? You've kind of touched on it a little bit earlier, but I was just wondering if you could tell me.

P2 It might encourage me to be more active because you can see what you're doing, and see how well you're doing and stuff like that.

P1 Yes. And what was it about the Fitbit that you feel stopped you from being active?

P2 I don't think it did stop me from being more active. There's no way of me being any more active than I am.

P1 Yes. And how about the GenieActive? How do you feel that stopped you from being active?

P2 Again, it couldn't exactly stop me, but maybe from being a bit too big, like you can't wear it for most sports.

P1 Yes.

P2 I think it would get in the way and stuff just doing activities.

P1 Yes. So the actual clunkiness of it would get in the way.

P2 Yes.

P1 Right, so a little rest for you. I'm going to ask your Mum some questions now. What do you think the benefits of wearing a physical activity monitor are for P2?

P3 Again what she said about she's very competitive, so I think the Fitbit made her see how exactly what she was doing, when some days she'd go. "Oh, we'll beat that", and so I think the benefit of it is to show her what she's doing, either doing enough or not, so there might be days when she thinks she's done well done enough, but I think it can show her that no actually, you need to do a little bit more today, you haven't done enough.

P1 Yes. And what do you think the negatives of wearing a physical activity monitor are for P2?

P3 She doesn't like attention being drawn to her, so for people who don't know she's got CF, you know they might say, "Well, what do you need to know how much activity you've done for?" And she might feel that she has to explain to them that she's got CF, and she doesn't want to.

P1 Yes. Which, you know, P2 shouldn't feel the need to have to, should she?

P3 And she's young, so she wants things to be fashionable and up-to-date, so I think a drawback for that is from a fashion point of view. One of them is all right, it's quite trendy. The other one, the....

P1 The GenieActive.

P3 GenieActive, yes. Which is like she says, she feels it looks a bit old-fashioned, and so that's a drawback just from that respect.

P1 Yes. And thinking about P2, do you feel that the activity monitors encouraged her to be more active?

P3 The Fitbit did, for sure. Oh actually, I think they probably both did a little bit initially, because, even though she couldn't see with the GenieActive what she was doing, I think she wanted to make sure you knew she was really active.

P1 Yes.

P3 That was recorded, and so I think just having that on, initially would have made her feel like doing more just to prove to other people and to herself that that's what she

was doing. But the Fitbit one definitely did, because she went, "Look what I've done today!" Oh, and she was back out on the trampoline doing a bit more, just to say, "Oh, I'm going to do more tomorrow" or whatever.

P1 Yes. So it spurred her on a little bit more, to beat her earlier results.

P3 Yes.

P1 OK. And what is it about the activity monitors that P2 tried, you feel encouraged her to be more active? You've kind of answered that already.

P3 Yes. Just the fact that she could see the, what's the dark one?

P1 GenieActive. No, I forget as well.

P3 GenieActive. It didn't help, because she didn't know how much she was doing, but the Fitbit showed her exactly what it was she was doing, and it encouraged her.

P1 Ah, thanks. And do you think it's feasible that for your child P2, wearing a physical activity monitor might influence the amount or type of activity that they do, that P2 does?

P3 I don't know if it'd influence the type, but it might influence the amount, just by the fact that it records what she does. Just knowing what she's doing, I think, just encourages her, because it's proved to us, so it's stopped us nagging, saying, "You haven't done enough". She can say, "Well actually, here you are. I have", or I can argue with her and say, "Well, actually, you haven't".

P1 Yes. So it sounds like you're saying it's the amount of physical activity P2 does.

P3 Yes, definitely. Yes.

P1 Ah, thanks for that. Back to you, P2. Just on the last bit now. So the GenieActive shows that on average you did seventy-nine minutes, which is one hour nineteen minutes of moderate to vigorous activity each day, and it's recommended that children and young people should do sixty minutes of moderate to vigorous activity each day to stay healthy. You did more than this. What do you think about that?

P2 I think it shows that, I think it's good, because I'm young.

P1 Yes. So it's good that it shows that you're doing enough.

P2 Yes.

P1 Yes. Can you tell me a bit more about that, what you mean by it shows that you're doing enough?

P2 It shows that the amount I'm doing, the amount of exercise and that, is enough to keep me healthy and well and that.

P1 Yes. And how does it feel knowing that you do that much, you know, you do enough?

P2 I think it is good, because, I don't know. It's hard to explain.

P1 It's hard to explain. That's all right. So the GenieActive also shows that you did the most moderate to vigorous activity on Wednesday. What do you think helped you to be more active on this day?

P2 That's the day that I do nothing, well not nothing, but the day that I don't have any planned sports, so I think I might have went on the trampoline more, or without realising how long I'd been on it for.

P1 Yes.

P2 I think it's the ones that aren't planned, like not effective, but in a way kind of, because you don't realise how much you're doing on it, because you're just kind of enjoying yourself. Not saying I don't enjoy the other ones. It's just because it's not a must. It's more that you've chosen.

P1 Yes. So you're taking it maybe you're doing it as you want to do it, rather than being instructed, if you like.

P2 Yes.

P1 OK. So the GenieActive shows that on average you spent four hundred and thirty-nine minutes, which is seven hours nineteen minutes, being sedentary, which means doing things while you're still, like sitting and watching TV. What do you think about that?

P2 That's kind of like a lot, but it's like over the amount that's supposed to be, well not supposed to be, but like average or whatever, of a normal person, child, and it kind of shows, kind of like working hard to do, that's supposed to make more.

P1 So you say working hard to do the amount that you should. Can you tell me a little bit more about that?

P2 It's like having like structured and planned things there that I go to each week so that I can stay healthy in that way.

P1 OK. And the GenieActive shows that you were more sedentary on Thursday and Saturday. What do you think about that?

P2 It's a bit weird, because I do gymnastics on a Saturday, and dance on a Thursday, so I don't know if that's one of the days that, I know with gymnastics I will have had to take it off, and if maybe on Thursday I forgot to put it on, so...

P1 So maybe we haven't had an opportunity to record those things.

P2 Yes.

P1 Oh, I see. And I was going to say, what was different about those days compared to Wednesday when you were most active?

P2 It's weird, because I do more sport on those days than I do on Wednesday, because I don't have anything planned on Wednesdays, so that's a bit weird. Well, not weird, but like shocking, kind of.

P1 Yes. So you weren't expecting that.

P2 Yes.

P1 Yes. OK. And you've kind of touched on it a little bit why you were surprised by that maybe, but I was wondering what was so unexpected about that?

P2 Because I do quite a lot of dance on Thursdays, because like a few hours, and I do gymnastics as well on Saturday, but the only thing with that, I had to take it off for Saturday, so I'm kind of not surprised, but am, by the Saturday, but I am surprised about the Thursday, but I think I might have either forgot to put it on, or when I put it on it was too late, because I'd already done the activities.

P1 Yes. Ah, thanks for explaining that to me. Did you change the amount of activity that you normally do, while you were wearing the physical activity monitors?

P2 I think if anything I went on the trampoline a bit more, and I might have gone in the street. It's like a game or something. I don't know, I might have. I think I kind of did, but it wasn't anything structured, so I didn't go, "Oh, I've got to running club today, because I've got the monitor on", but obviously because I don't really have much time to do any other activities, and some days you just need to have a break.

P1 Yes. So you say that you might have done a little bit more like trampolining or playing out and doing games outside. How often do you reckon you would have done that?

P2 Only like once a week maybe. Well, I think I went on the trampoline not more than once a week, but I'm not sure. I don't think I went on it on the Wednesday, because I didn't do anything, and I might have not done anything, but I don't know.

P1 OK. Can you give me an example of how wearing the monitors may have made a difference to the activities you do?

P2 Is this just the GenieActive or the....?

P1 The black one's the GenieActive, and that one's like the clunky one like a watch.

P2 Yes, I mean is this about both of them, or...?

P1 Yes, just both of them, really.

P2 Yes, with the Fitbit you can see what you've done, and I think with the GenieActive it's trying to do more, in a way so you can prove that you've done something. But then in a way you're kind of not, it makes you not want to do the thingy with the, not not want to do it, but not do as much, because you can't see that you've done that, or how much activity you've done straight away, with the GenieActive.

P1 Yes. Ah, thanks for explaining that to me. On the feedback questionnaire you said that you disagreed that you tried new activities out while you wore the activity monitors. Can you tell me a bit more about that?

P2 Well again, I didn't really have much time, because most of my days are well, not taken up, but you know, with the other stuff that I do, so I can't do any more than I'm doing at the minute.

P1 Ah, yes. Thanks for that. And did you notice a change in how strenuous the activities you typically do are?

P2 Not really, because I can tell what's more, I was going to say more hard, what's harder, and what's not as hard, so I think it's kind of just the same since. They never emphasised to me how strenuously. I just kept it the same.

P1 Oh, so you kept your activity level the same? Is that what you mean?

P2 Yes.

P1 OK. And can you tell me about how you used the Fitbit?

P2 I put it on my ankle, because again you could put it under your sock, and you can like hide it, in like basketball, and it's easier on your wrist than your ankle, and gymnastics, rather than on your wrist, because if you're doing bar work, it might get in the way, because you have to use your wrists with most of the pressure's on your wrists, so if you've got something on there, you might hurt yourself, so I probably would have had to take it off if it was on my wrist.

P1 Yes. And what settings on the Fitbit, like what settings did you use on the Fitbit?

P2 I tried the day ones, and I tried the night ones, but it kind of confused me, because you don't know whether you've pressed it right, or whether you've done it wrong, so you don't know during the day whether have I got this on night mode or have I got it right? And it's a bit confusing with the amount of taps and stuff.

P1 OK. And apart from the step counter on the dashboard, for example, did you use any other settings on the Fitbit dashboard?

P2 I think it was mainly, I can't remember off the top of my head the other ones that I used, but again I tried to use the sleep one, but the dashboard thing, it said that I didn't do it, so it kind of like made me a bit like I wanted to smash it.

P1 So it sounds like it was just a bit confusing, and kind of contradictive to what it said it had done, when you know it hadn't done it, is that what you're saying?

P2 Yes.

P1 OK. And what was it you liked most, you know on the dashboard, the step counter and the sleep function?

P2 Because you can, with the sleep thing, even though it didn't exactly work, you could kind of, if you wanted to see how well your sleep patterns was and that, and with the step counter, it kind of shows how many steps you've done, so you know much walking you've done and stuff.

P1 OK. And what did you dislike most about the Fitbit dashboard?

P2 It was a bit confusing, with too much going on, so it was hard to use, again the sleep thing, and at first it was hard to get things started with it.

P1 And what would you feel you'd change to make it a little bit easier, so to get it started and to make the sleep function a little bit easier?

P2 Maybe change, not exactly the dashboard, but change the actual wrist thing, so that you could see whether the sleep was on, or whether it was off, or whether it was properly working or not.

P1 Yes. Ah, so maybe change the actual wristband.

P2 Yes.

P1 OK, thanks. So you mentioned on the feedback questionnaire that you agreed that getting some information each day would encourage you to do more activity. Can you tell me a bit more about that?

P2 Say if I didn't do as much on a day that I thought I did, I could go on the trampoline more or maybe start running or something, but it's like showing that information, showing whether I was doing enough or whether there was room to do more.

P1 Yes. Ah, thanks. And what is it about getting some information that tells you how active you are, you feel would encourage you to be more active?

P2 Maybe again, because I'm quite competitive, if that information was there, I could try and beat it.

P1 So you could try and beat it.

P2 Yes.

P1 OK. So you said that you would like to wear a Fitbit again, but you didn't agree or disagree that you would like to wear a GenieActive again. Can you tell me a bit more about that?

P2 Although the GenieActive kind of did the job, it was hard to explain to people that it wasn't a watch, and because it was quite clunky you couldn't wear it for sports and stuff like that, so I think it was a bit, not complicated, but a bit irrelevant kind of, for the sport that I did, because you wouldn't be able to wear it to do the job that was needed, whereas the Fitbit, you could put it in your sock or wear it without it being shown and then still did the same job as what it was supposed to do.

P1 Yes. Ah, thanks for that. And last question. If you could ask the clinicians in the CF team, so like the doctors and nurses and dieticians and everything in the CF team anything, what would you ask them?

P2 I don't really know. I don't have many questions about CF and sport and stuff, because I know that I do the most I can. There's not much, well, there's nothing more that I could fit in, because obviously I need like peace, rest and stuff like that sort of thing. There's not any questions that I'd ask.

P1 That's fair enough. Aah, thank you very much for speaking with me today. I've really enjoyed hearing about what you thought about wearing the physical activity monitors. So that's the end of the interview now. Is there anything more you'd like to add? Anything at all, maybe something I've missed? How about you, Mum and Dad. Anything I've missed?

P3 I can't think of anything. I think she enjoyed wearing it, and I think the Fitbit was a great idea for P2, and she said a few times because of how competitive she is, I think it's a really good idea from the perspective that it gets us off her case as well, because you can prove to us that she's doing what she's supposed to be doing vice versa, but it also gives her a little bit of responsibility for it. She likes to do things for herself, so for

kids who are independent, or want to be more independent, it can take a little bit more ownership of their activities, and then go, "Here we are, look. This is how much I've done", and it'd probably cause less friction in some houses.

P1 Yes. Because it's a hard copy record, isn't it?

P3 Yes, you know, for both, because some parents are like, "You need to do this, you need to do that", and I know of some kids who have their parents pushing them all the time, and I think it'd be really good for some children who can prove to their parents that they're doing, and maybe it'll be a little bit less responsibility for some parents as their kids are getting older, and it'll be a fun way of children taking up some responsibility for how much activity you do.

P1 Yes. How do you think it'd be for parents sort of relinquishing that responsibility in that situation?

P3 Well, for some it'd probably be difficult, but for me personally, I see my role as a parent as my kids are dependent. As a parent I'm supposed to give them skills for life, for them to be able to be self-reliant, and there's stuff that I shouldn't have to do once they get to a certain age. If they're old enough and able to do it, they should be doing it.

P1 Yes.

P3 Some parents just want to keep the kids to themselves, want to be their Mummies and do for them, which that's ok, that's fair enough. That's just a different style of parenting.

P1 Yes. That's fair enough. Thanks very much. OK, I'll end the interview now, but thank you very much for your time, and for speaking to me today.

End of Interview

Phase 3 – P8

I OK, so hi, P2. If you remember the last time we met spoke to you about when you're active and when you're not active, and then I asked you to try out two different activity monitors. One was a GenieActive and the other was a Fitbit. I'm here again today to ask you thirty short questions about what you thought about what you thought about the devices you tried out, like what you liked about them and what you disliked about them. There's no right or wrong answers, so don't worry. I'm interested in what you have to say, and may I ask for some examples if you can, because this'll help me to understand your experiences better. If you need to think for a little bit before answering a question that's ok. We won't try and answer for you. We'll just wait until you feel ready to answer, or if we move past a question and then you remember something that you wants to say, that's fine too. You can add things at the end. That's not to exclude your Dad. There are some specific questions for him also, so watch out for these. It should take around forty minutes depending on your answers, and I'll be recording our conversation on the Dictaphone just to help me remember everything we say. Also I'll be ticking off the questions that I ask as we go along just so I don't say the same question twice. If you or I happen to mention someone's name as we go through the questions, or anything that might let someone else know who are or other people, I'll be taking this out of the transcript of our conversation, and this is just a document where we write out our conversation. So if you're happy to begin?

P2 Yes.

I OK. In this first section I'm going to ask you about the times and places that you wore the activity monitors we asked you to wear. On the feedback questionnaire that I asked you to complete after wearing each device, you said that you agreed that you enjoyed wearing the physical activity monitor. Can you tell me a bit more about that?

P2 It was good to wear, but it stopped me from doing one thing.

I And what was that thing that it stopped you from doing?

P2 A dance lesson.

I Oh, right. Can you tell me a bit more about that?

P2 Well, you're not allowed to wear jewellery in the dance lesson.

I OK.

P2 Yes, that's basically it.

I So what did you like about wearing the physical activity monitors?

P2 It was comfortable. It didn't get in the way that much. Yes, basically it's just like a watch.

I OK. And was that for both of them?

P2 Yes, for both of them.

I And what did you dislike about wearing the physical activity monitors?

P2 You know when you wear it? It pushed against my skin and left a bruise there for a couple of days, and it really hurted.

I Oh dear! And was that for both of the devices?

P2 Yes.

I Was it? Oh dear! And how come do you think that happened?

P2 About every couple of days, and if I kept doing stuff that it pushed against it or do that.

I So if you put your hand up and then if you put your hand out, sometimes that pushed against you a little bit. OK, thanks for that. What physical activity monitor did you enjoy wearing the most?

P2 The second one. The Fitbit.

I The Fitbit, yes? That's right. And what was it about the Fitbit that you enjoyed, compared to the GenieActive, which was the other device?

P2 It was a lot more comfortable, the Fitbit. It looks slicker, it looks smarter than a big bulky thing. You didn't see the insides and all that.

I So it looks slicker, and it was more comfortable.

P2 Yes.

I And was there anything else?

P2 No, not really.

I No? And on the feedback questionnaire you said that you agreed that the GenieActive was comfy to wear, and that you didn't agree or disagree that the Fitbit was comfy to wear. What was it about the GenieActive and Fitbit that you felt made it comfy to wear?

P2 The thing that it was made out of.

I So like the material that it was made out of?

P2 Yes.

I Can you tell me a bit more about that?

P2 It didn't rub against your skin as much as normal leather.

I Yes.

P2 Like leather with things coming out of it, if you get what I mean.

I So like the texture of the leather?

P2 Yes.

I So compared to leather the texture was maybe a bit better for you?

P2 Yes.

I OK. And what was it about the GenieActive and Fitbit that made it uncomfy to wear?

P3 Was that the bigger one, was it?

I The GenieActive was the bigger one.

P2 It was because, you know the metal bit?

I Yes.

P2 It kept hitting there.

I Ah, so it kept hitting your wrist?

P2 Yes, a bit of my wrist. That's basically what it did.

I And was the metal bit on the GenieActive?

P2 Yes. Not on the Fitbit, though.

I Was there any bits about the Fitbit that was uncomfy for you to wear?

P2 No, not really.

I No? And you said that you disagreed that the GenieActive and Fitbit got in the way of what you were doing. Can you tell me a bit more about that?

P2 Only the GenieActive did, because it was classified as jewellery, but I hid it, the Fitbit, because it was smaller, and underneath just looked like a wrist-like thing.

I Ah, right. And when you say the GenieActive got in the way of what you were doing, can you tell me a bit more about that?

P2 When I had to go on the hoop and circus skills, you know like I said before, when I push my hand up, like flex my hand, it hits the bone there.

I Ah, so the bones on your hand?

P2 Yes. It hurts.

I Can you give me an example of when the monitor got in the way of what you were doing?

P2 I'm not really sure, you know. It's just every time I flexed my hand.

I Every time you flexed your hand. And would that matter what you were doing, or would it matter what you were doing?

P2 It wouldn't really matter. It's just every time I flex it.

I OK. And what would you do to change the GenieActive and the Fitbit if you could do anything to make it better?

P2 Make it not a wrist thing.

I OK.

P2 Either like a thing that you put there.

I So sort of behind your ear?

P2 Yes, behind my ear. Or in your pocket.

I Yes.

P2 Yes, and you know like on the Fitbit, it was like the chip, one of them, instead of just like a whole thing.

I Ah right, so the Fitbit's got, like you say, a little chip that goes inside the plastic wristband. You would prefer it if it was just the chip, and you could maybe put that in your pocket or behind your ear.

P2 Yes.

I OK. What about the GenieActive? Would there be anything that you'd change to make that better?

P2 Make it thinner. Make it a bit tighter, because every time it just kept slipping off, and make it an easier way to put it in, because it's just really hard, because it keeps hitting my wrist in between my hand and my wrist.

I OK, so make it a bit easier on your wrist.

P2 Yes.

I OK. Did you ever have to be told to remove the monitor?

P2 Yes.

I Can you tell me about that?

P2 A couple of times in dance.

I OK. And who told you to remove the device?

P2 My teachers.

I And what reasons did they give you?

P2 In case it comes in the way.

I In case it comes in the way?

P2 Yes.

I OK.

P2 I mean gets in the way.

I Gets in the way. OK. Next I'd like to talk about other people like friends and family, and how they may have influenced you to wear the physical activity monitor. Did you ever forget to put the physical activity monitor on?

P2 In total for both of them, seven days.

I And did you ever forget to put it on?

P2 Yes.

I OK. Can you tell me a bit more about that?

P3 In the shower. You kept on leaving it in the bathroom, didn't you?

P2 Yes.

I So you kept on leaving it in the shower after you took it off?

P2 Yes.

P3 There was one of them one you couldn't wear in the shower, wasn't it?

P2 Yes.

P3 Or was it both of them?

P2 No, it was the Fitbit I couldn't wear in the shower.

P3 Is that the big watch?

P2 No, the small one.

P3 I thought it was the big watch you couldn't wear in the shower.

P2 No.

I So is it just the Fitbit that you forgot to put on after the shower?

P2 Yes.

I OK, and the GenieActive, did you leave that on while you were doing water sports or while you were in the shower?

P2 Yes.

I How was it remembering to put that on?

P2 I just wear it like a watch.

I Yes?

P2 Basically you don't take it off.

I OK. And on the feedback questionnaire you said that you disagreed that you kept forgetting to put the Fitbit on, but that you didn't agree or disagree that you kept

forgetting to put the GenieActive on. How was it for you remembering to wear the monitor each day?

P2 Pardon?

I So on the feedback questionnaires you said that you disagreed that you kept forgetting to put the Fitbit on, but that you didn't agree or disagree that you kept forgetting to put the GenieActive on. How was it for you remembering to put the monitor on each day?

P2 Good. I slept in it.

I Yes. and would that be for both?

P2 Yes.

I OK. I was going to say can you tell me how you remembered to wear each monitor, the monitor each day. Is that the way that you remembered?

P2 Yes. I just kept it on all night. That's basically it.

I And you said that you strongly agreed that other people noticed that you were wearing the Fitbit. What did people say to you?

P2 they said, "What's that?" and then I told them.

I And what did you think about what they said?

P2 Totally normal.

I Totally normal?

P2 Well basically it's like a watch.

I Yes?

P2 Yes.

I And what did you say to them when they asked?

P2 I said it's a thing that records how much exercise I do.

I And what did they say back?

P2 Oh, ok.

I So they seemed ok with that?

P2 Yes.

I How did that make you feel?

P2 I didn't have really any feelings at that moment.

I No?

P2 It's just like, you know when you have a plain conversation.

I Yes.

P2 Yes, basically that.

I So it was just a plain conversation.

P2 Yes.

I OK. Did you notice a change in the amount of active activities you normally do as a family while you were wearing the physical activity monitors?

P2 No.

I No? And how about with your friends? Did how your friends are involved in you being active change?

P2 In that time period I didn't go anywhere really. I just stayed in school.

I Yes? In the class. And did the way you play with your friends change while you were wearing the monitors?

P2 I didn't really play with my friends that much.

I No? Is that while you were wearing the monitors?

P2 I was quite ill.

I Oh, right. Can you tell me a bit more about that? You say you weren't well.

P2 I had a virus. I still have it now. I've had it for ages now.

I And did that affect how you played with your friends or the way that you get involved in activity with your friends?

P2 Yes.

I Yes? And can you tell me how it affects how you get involved in activity with your friends?

P2 It's because I was ill. I couldn't go outside, but once I had rosy cheeks, this little thing my Mum makes me go.

I OK. So when you get rosy cheeks, that's when you can go out?

P2 Yes.

I And what makes you get rosy cheeks?

P2 I don't know.

I You're not sure? That's ok. tThanks, P2. On the feedback questionnaire you said that you strongly disagreed that other people like friends or family kept asking you how much activity you did while you were wearing the GenieActive. What do you think about that?
t?

P2 Totally normal. They didn't ask me like, "Are we doing this?" and two, because I'd just ignore them.

I Because you just ignored them?

P2 Yes.

I OK. So that didn't happen for you. And how about for the Fitbit? Or rather, for the Fitbit you agreed that other people like friends and family kept asking you how much activity you did while you were wearing the Fitbit. What do you think about that?

P2 What was the question?

I Yes, course. For the Fitbit you agreed that other people like friends or family kept asking you how much activity you did while you were wearing the Fitbit. What do you think about that?

P2 They didn't ask me. Yes, basically, they didn't ask.

I Ah, so they didn't ask you. And was that for both devices that they didn't ask you?

P2 Yes.

I OK, thank you. Thank you for your answers. I'm learning a lot about what

wearing the physical activity monitors was like for you. In this last section I'm going to explore how wearing a physical activity monitor may or may not have affected the activities you do. So do you think that wearing a physical activity monitor encouraged you to be more active?

P2 No, not really.

I Not really, no? And how about other young people with CF? Do you think wearing the GenieActive and Fitbit would encourage them to be active?

P2 Yes.

I Yes?

P2 Because...

P3 He does like to dance. He does like nearly thirty hours of dance a week, so he's got more activity, so we found that he stopped putting weight on because he's been a bit too active.

I Yes. So it's a balance, then?

P3 Yes.

I Yes.

P3 So when you say has he been more active, I don't think he could be more active than what he is.

I No, that's fair comment, isn't it?

P2 I'd turn into a stick.

I Oh no, well we don't want that, do we? We definitely do not want that.

P2 I'm only four stone.

I` Aaah! Well, you know, you look good. You look good on it. So you said that, when I asked you how about other people with CF, do you think wearing the GenieActive and Fitbit would encourage them to be active, you said yes. Can you tell me a bit more why you think that might be.

P2 Because they have like a goal to reach, you know with the Fitbit. I would recommend it more, because it has like little lights, and when you fill up all the lights, then you can keep checking on the website every day seeing how much you'd done in total, was it?

I	I think it was in total, yes, and it adds it up, doesn't it?

P2	Yes.

I	And how about the GenieActive? Do you think that would encourage young people to be physically active?

P2	No, because it's just a bulky thing that has nothing on it, just... it's like a piece of paper, a plain piece of paper. What would you rather have, plain piece of paper or a coloured paper?

I	Ah, I think that's a no-brainer, isn't it? You'd rather have the coloured paper. And do you think that's true for other people with CF?

P2	Sometimes. People may have different opinions, but that's my opinion.

I	And how do you think using the GenieActive and Fitbit could help you to be more active?

P2	I'm not really sure, you know.

I	Yes.

P3	He couldn't be more active.

I	No. What do you think about that?

P2	I'm not sure. It's like doing loads.

I	You're already doing loads.

P2	Yes.

I	How would it be for you to try and fit more in?

P2	Life sentencing.

I	Life sentencing? What does that mean?

P2	Oh, I thought that was a word. Life sentence, Death Row.

I	Death Row? That's a significant...

P3	He said that he does that he does that much exercise that he can't do any more exercise.

I Yes.

P2 I'll have to eat this house.

I You'd have to eat the house.

P3 The cost of eating the food would be too to replace the calories.

P2 In class, getting a couple of thousand Scandishakes, so I need to keep drinking.

I Aww! And how would it be for you to do a little less activity?

P3 We wouldn't let him do any less activity. He loves it anyway, don't you?

P2 Yes. For CF people, they can either be like, some people take, is it the anorexias that can't eat, that don't, like they think they're fat?

I Yes.

P2 It's like that. They think that sort of, that like they're thin, if you get what I mean, and then you've got the middle that are like I am. They're doing a lot of exercise, but going up and down. And then people who don't do any exercise who have cystic fibrosis, well, they do barely any exercise, and they don't get fat. They like, you know the attributes of getting a bit obese, like you run out of energy a lot faster than the average CF patient who runs out of energy say if they run a mile. They'd probably run out of energy then and have to have a few minute breather.

I OK. So, you know, you said that to do more activity would be life sentencing and Death Row.

P2 Yes.

I And when I asked how would it be for you to do less, it seems like that might not be something that you'd consider. What do you think is the right amount of activity for you to do?

P2 Eighteen hours.

I Eighteen hours?

P2 No, it's six, eight, nine, ten and a half, ten and a half hours of dance each week.

I Yes?

P2 Like exercise, or ten, maybe five, on a daily basis.

I	Yes? So five hours a day, do you mean?

P2	Yes, at least of running around doing stuff, and not being lazy.

I	Ah, so you mean like maybe ten hours of dance a week, and then other activity on top, just running round?

P2	Yes.

I	That adds up to around five hours a day?

P2	Yes.

I	Ah, thanks for that answer, P2. That's brilliant.

P2	That's terrible maths.

I	No, I got what you were getting at. Can you tell me how you think using the GenieActive and Fitbit was useful or not useful at encouraging physical activity?

P2	It was useful to track how much I'd done, because one time, you know when I got the Fitbit, I had three hours, and guess how many steps I did.

I	Is that at dance, how many? Oh, gosh!

P2	Yes. I did, well, I filled it up in three hours.

I	That's fantastic! And was that with the Fitbit?

P2	Yes.

I	OK. And how do you think that using the GenieActive and the Fitbit was useful or not useful for encouraging physical activity?

P2	It wasn't that useful for encouraging me. It may be different for other people, but I'm not really sure with it.

I	OK. So what do you think it was about the GenieActive and Fitbit that may have stopped you from being active?

P2	Like I said before, it gets in the way sometimes, like rarely, but often, like weekly maybe.

I	OK, just weekly. And what was it about the GenieActive and the Fitbit that you feel encouraged you to be more active?

P2 I'd rather have the Fitbit than the GenieActive, because it looks cooler and slick, and has lights, instead of just this thing that you see a chip, and so, "Hi, I'm green".

I And it doesn't really do anything, does it?

P2 It doesn't really do anything.

I Ah, right. So it sounds like you're saying that you preferred the Fitbit, because you can actually see things, whereas the GenieActive...

P2 It was just like on a watch, and a watch that's broken.

I Is that how it felt for you?

P2 Yes.

I Aww, that's brilliant. Thanks, P2. Some questions for you, Dad. Is that ok?

P3 Fine. Go on.

I What do you think the benefits of wearing a physical activity monitor are for P2?

P3 For P2 in particular, none really, because as I said, we're quite on the ball with his exercise.

I Yes.

P3 So it didn't really have any effect, did it? And unfortunately when you did give him it, he was going through a few bouts of illness, so it didn't give a real good impression of what he normally does.

I Yes.

P3 And also remembering to put it on, wasn't it, was the big thing? And then what was the one that needed charging all the time?

I Ah, the Fitbit.

P2 The Fitbit.

P3 Yes, that was, and remembering where you'd left it, because we've got that many USB ports in this house. It was just lie, "Which one is it?"

I So overall it sounds like you're saying you don't feel it was really useful for P2.

P3 It wasn't useful for P2, but maybe for someone who's got targets to hit and that, and had to prove that they were doing exercise, yes, yes, it would probably be a good thing.

I Yes. Well, as you say, P2 does such a lot anyway.

P3 Yes.

I What do you think the negatives of wearing a physical activity monitor are for P2?

P3 Just the comfort side really and the convenience side. As I said, it was a constant having to remember to put it back on.

I Ah, yes.

P3 You know, after being in the shower or having to take it off because he was dancing, and then obviously it was quite hard for him to remember all that, was it, mate?

P2 Yes.

I And thinking about P2, do you feel the activity monitors encouraged him to be more active?

P3 No. We always encourage him to be active, don't we, mate?

P2 You don't make me do stuff.

I That's fair comment. And what goes through your head to make him not stop, if that makes sense, if that question made any sense?

P3 He knows what the ramifications are if he doesn't.

I Yes.

P2 I die. No, I don't die, I get a tube stuck in my stomach and have to get fed every day.

P3 Pipe feeding.

I Pipe feeding. And how does that feel for you?

P3 He doesn't like the idea at all, do you, mate? It's just, pipe feeding is just another way of introducing infections into you, a child whose immune system's down anyway, so it's detriment.

I Sounds like you'd rather avoid that route.

P3 Yes, yes. Yes, please.

I And do you think it's feasible that for your child, i.e. P2, wearing physical activity monitor might influence the amount or type of activity that he does?

P3 No help at all. None at all. As I said, he does what he's got to do.

I Yes.

P3 And he hasn't got much choice about that, have you, mate?

P2 No.

P3 He tries to wag it every day, don't you, babe?

P2 No. I don't know what you're talking about.

P3 The boy who cried wolf, mate.

I Aah! Has P2 always preferred dance? Has that been his preferred activity?

P2 Swimming.

P3 Swimming. He used to go swimming, and then he started dancing, and he's really got a talent for it.

I Yes. Thanks for that.

P3 OK.

I Back to you, P2. So the GenieActive shows that on average you did two hundred and sixty-three minutes, which is four hours twenty-three minutes, of moderate to vigorous activity each day. It's recommended that children and young people should do sixty minutes of moderate to vigorous activity each day to stay healthy. You did more than this. What do you think about that?

P2 Celebration!

I Celebration?

P2 Yes, I think it's good, because say if, say a bodybuilder, they don't want to stay at the certain match. They want to maybe, some want to be bodybuilder champions, they want to reach some goals, I'm trying to speak louder. Well, basically it's like you have a goal, a set time limit, maybe someone wants to go, "Actually, let's not do that. Let's

break the rules." Like, yes.

I So from the activity that we got for you, the levels that we got for you, you do a lot more than sixty minutes. When I asked you what you thought about this you said celebration.

P2 Yes.

I So can you tell me a bit more about that, what celebration means to you, what that means?

P2 Celebrate you've achieved something.

I It feels like you've achieved something?

P2 Yes.

I OK.

P2 Better than the average human.

I Better than average human.

P2 Yes.

I And the GenieActive shows that you did the most moderate to vigorous activity on Saturday. What do you think helped you to be more active on this day?

P3 You've got LAMDA on a Saturday. Which Saturday was it?

I I can't remember, to be honest with you.

P3 It'll be LAMDA, won't it, or something?

P2 Yes, because it wouldn't have been last Saturday.

P3 Or was it when you went swimming with E? You did that thing ages ago, didn't you?

I It would have been, let me think, probably around April time. It would have been a while ago.

P2 Oh, I went to the Yellow Submarine.

I The Yellow Submarine?

P2 Yes.

I OK. So how did going to the Yellow Submarine rather on that day help you to be more active?

P2 Because I was running around crazy.

I Running round crazy. OK. The GenieActive shows that on average you spent three hundred and fifty-four minutes, which is five hours fifty-four minutes, being sedentary, and this means doing things while you're sitting, or while you're still rather, like sitting and watching TV. What do you think about that?

P2 Probably do the handstands.

I Do handstands?

P2 Oh headstands, you know, while I'm sitting round the room.

I How would that change things?

P2 Because I'm moving.

I Because you're moving?

P2 Yes.

I OK. And what would moving more, in that sense, what do you think would be the purpose of that, or the importance of that?

P2 I don't know really. I just do it randomly sometimes. Once I was doing cartwheels.

I Ooh, I was no good at doing cartwheels, I'm afraid.

P2 Well, basically once I did one of them without even concentrating. I didn't even know I was doing one.

I Oh, so it sounds like you say that you tend to be quite active anyway, even when you're not thinking about it.

P2 I multi-task.

I You multi-task, ok. The GenieActive shows that you were more sedentary on Sunday and Monday. What do you think about that?

P2 I've got my day off on Monday.

P3 And he might have been sick on that Sunday. In fact he was probably sick on the Sunday, weren't you?

P2 Yes, I was in April once.

P3 Yes.

I OK. And what was different about those days compared to Saturday when you were most active?

P2 I don't know, you know.

I Are you not sure?

P2 I'm not really sure.

I You're not really sure. That's ok, P2. You're doing really well. Did you change the amount of activity that you normally do while you were wearing the physical activity monitors?

P2 I minused one lesson, and gained one.

I OK, so do you think you kind of did more or do you think you kind of did less?

P2 I did a bit more.

I You did a bit more? And can you tell me about the difference wearing a physical activity monitor made to you?

P2 It felt comfortable. I'm not really sure.

I Can you give me an example of how the physical activity monitor made a difference to the activities you do?

P2 It didn't really make a difference. It didn't make a difference at all. It was just there.

I OK. Do you think it didn't change the amount of activities that you normally do?

P2 No.

I No. OK. On the feedback questionnaire you said that you strongly agreed that you tried new activities out while you were wearing the physical activity monitors. Can you tell me a bit more about that?

P2 I started something else. Like I said before, I did the other lesson.

I And what other lesson was that?

P2 I can't remember.

I You can't remember.

P2 It was an hour lesson, though. I got rid of a half an hour lesson to go to an hour lesson.

I And would it have been dance-based?

P2 Yes.

I Yes. OK. So that's the other thing. And what was it about wearing the GenieActive and Fitbit that encouraged you to try new activities?

P2 Well, it wasn't really that that encouraged me. It's just because we didn't have enough money to do the swimming lessons, so we swapped out.

I OK. That's fair enough. Did you notice a change in how strenuous the activities you typically do are? Like how fast you run, or while you were dancing?

P2 No.

I And can you tell me about how you used the Fitbit? You know, like what settings on the Fitbit you used.

P2 What do you mean?

I So on the Fitbit, like you said, before you got the tablet.

P2 I kept it on normal mode when I was awake, sleeping mode when I was asleep. When I felt I was about to go to sleep I tapped it to go to sleep mode. Yes, that's all.

I So you just kept it as normal, and then for the night you used the sleep mode.

P2 Yes.

I OK. So did you look at the stats counter at all on the dashboard?

P2 Sometimes, yes.

I And did you, apart from the stat counter, did you use any other settings on the Fitbit dashboard?

P2 What do you mean?

I So when you went on the computer, did you use any other settings or look at any of the things on there?

P2 No, I didn't do any of that.

I You didn't? OK.

P2 Because we don't have a computer.

I Ah right, ok.

P3 Well, we've got laptops, but we didn't have a charger at the time.

I Oh yes, yes.

P3 The first thing that goes, isn't it, the battery, and then we've all got iPads now, so...

I Yes.

P3 We tend to use our iPads, as you might have noticed.

I And what was it that you liked most about the Fitbit dashboard, or the Fitbit in general?

P2 It felt comfortable, it looks cool, it wasn't bulky like the other one. Yes.

I Yes. And did you ever get a chance to look at the dashboard? I know your Dad said that you didn't have a computer.

P2 No.

I Ah no, that's fine. And you mentioned on the feedback questionnaire that you strongly agreed that getting some information each day would encourage you to do more activity. Can you tell me a bit more about that?

P2 Did I say that?

I So on the feedback questionnaires, you circled that you agreed, or strongly agreed, that getting some information each day would encourage you to do more activities. Can you tell me a bit more about that?

P2 Well, it's giving, "Oh, you reached this. Do you want to reach this?" Yes, goals, basically.

I To fulfil your goals?

P2 Yes.

I OK. And was that for both of them, or was it just...?

P2 No, just for the second one.

I The second one. Just for the Fitbit. And what was it about getting some information that tells you how active you are each day, do you feel would encourage you to be more active?

P2 Because the goals.

I Because of the goals?

P2 Yes, that you reach.

I It sounds like you're saying that by having goals it gives you something to work towards.

P2 Yes.

I And what goes through your mind when you've got things to work towards like goals?

P2 I don't know.

P2 You don't know. That's ok. Nearly at the end now. Just a couple more questions. You said that you would like to wear a GenieActive and a Fitbit again. Can you tell me a bit more about that?

P2 Can I change something?

I Yes, course, go on.

P2 I wouldn't like to wear it again, but I would recommend it to someone else.

I OK. And would that be for both devices?

P2 No, just for the Fitbit. Just for the Fitbit, because the Fitbit was cooler.

I So just for the Fitbit. Sorry, just so I can understand. So you're saying that you'd like to wear the Fitbit again, or you'd prefer somebody else to do it?

P2 Somebody else wear it. I mean, both of them are good, but I would recommend the Fitbit more.

I The Fitbit more for someone else. And why is it that you'd recommend the Fitbit more, say, than the GenieActive for someone else?

P2 Like I said before about the thing where it's slicker, it doesn't get in the way as much, yes.

I Ah, right, so for all those reasons that you've said before. Thanks, P2. Last question now. If you could ask the clinicians and the CF team at Alder Hey anything, what would you ask them?

P2 About the things?

I Just anything really.

P2 I'm not sure.

I You're not sure? If it was something about the physical activity devices, what would you ask them?

P2 Can you get them in different colours?

I Can you get them in different colours? Just different colours?

P2 Yes.

I OK. Thank you very much for speaking to me today. I've really enjoyed hearing about what you thought about wearing the physical activity monitors. That's the end of the interview now. Is there anything you'd like to ask?

P2 No, not really.

I Maybe something I've missed?

P2 No.

I No? OK then, I'll end the interview now, and thank you very much for your time and for speaking to me today.

P2 Thank you.

I Thank you very much, P2.

End of Interview

Phase 3 - P9

P1 So hi, P2. If you remember, the last time we met I spoke to you about when you're active and when you're niot active, and then I asked you to try out two different activity monitors. One was a GenieActive, snd the other was a Fitbit. I'm here again today to ask you thirty short questions about what you thought about the devices you tried out, like what you liked and what you disliked about them. There's no right or wrong answers, so don't worry. I'm interested in what you have to say, and may I ask for some examples if you can, because this'll help me to understand your experiences better. If you need to think for a little bit before answering a question that's ok. We won't try and answer for you. We'll just wait until you feel ready to answer, or if we move past a question and then you remember something that you wanted to say, that's fine too. You can add things at the end. That's not to exclude you, Mum. there are some specific questions for you also, so watch out for these. It should take around forty minutes depending on your answers, and I'll be recording our conversation on the Dictaphone just to help me to remember everything we say. Also I'll be ticking off the questions I ask as I go along, just so I don't say the same question twice. If you or I happen to mention someone's name as we go through the questions, or anything that might let someone else know who you or other people are, I'll be taking this out of the transcript of our conversation, and this is just a document where we write out our conversation. So if you're happy to begin.

P2 Yes.

P1 In the first section I'm going to ask you about the times and p;laces that you wore the activity monitors we asked you to wear. On the feedback questionnaire that you completed after wearing each physical activity monitor, you said that you disagreed that you enjoyed wearing the GenieActive. Can you tell me a bit more about that?

P2 It was just a bit thick, and it got in my way of doing general activities I do every day, and I had to be careful that I didn't knock it and stuff like that, and it was quite thick. People noticed it a lot more, and was asking me about it, thinking it was a watch, and then when they actually saw it there was no watch, so that was, I was just a bit like, oh...

P1 And I can see you said it got in the way of your general activities. Can you think of an example of when it got in the way?

P2 Like on the trampoline if I'm bouncing and I land on my wrist or something like that, or I'm on some bar, because I've got to be careful that it doesn't dig into me or knock it, and if I'm doing swimming at school, I've got to be careful thst I don't whack it on the wall doing swiming, and stuff like that.

P1 That's brilliant. Thanks, P2. For the Fitbit you said that you agreed that you enjoyed wearing the Fitbit. Can you tell me a bit more about that?

P2 I could check up on what I was doing during the day, and it was a lot smaller. It just looked like a jelly bracelet. It didn't look like nothing more, and it was fun, and my friends were getting involved in it as well, which was really good, because they were asking

questions, which I don't mind what they were asking, like how many steps, and they were encouraging me to do more, so I think that was good as well. I enjoyed wearing that.

P1 OK, so what did you like about wearing the physical activity monitors?

P2 On the Fitbit one, I liked the fact that everybody in my class was getting a bit involved in it, asking me questions, which I liked, because I don't mind answering them and doing steps and that,a nd my first one, the...

P1 GenieActive,

P2 The GenieActive one. I enjoyed wearing that because at the end, I think, I did quite well with it, and I tried to beat my high score every day. Even if I didn't know what it was, I was trying to work out, so I think I enjoyed working out, doing a bit of the maths on it.

P1 OK. And what did you dislike about the GenieActive and Fitbit?

P2 I disliked on the GenieActive because it got in the way, and it was thick, and it looked a bit like a man's watch. And the Fitbit, you couldn't get it wet, so if I was doing swimming I'd have to take it off. If I was going, like we went canoeing with the school, we wore waterproofs, and it ran out of battery, and I thought it broke, so that made me into a bit of a panic, but yes.

P1 Thanks. That's brilliant, P1. What physical activity monitor did you enjoy wearing the most?

P2 The Fitbit one.

P1 The Fitbit. And what was it about the Fitbit that you enjoyed compared to the GenieActive?

P2 The Fitbit was more lighter on my wrist, and I could sleep in it, and I could see my patterns, and we could see where I get my hyperactivity from, and the GenieActive one you couldn't really check up, and you couldn't really sleep in it, and it was chunky, and it just got in the way really.

P1 Ah, thanks. P1. On the feedback questionnaire you said that you disagreed that the GenieActive was comfy to wear, and that you didn't agree or disagree that the Fitbit was comfy to wear. What was it about the GenieActive and Fitbit that made it not comfy to wear?

P2 The GenieActive was quite heavy, and it was thick, and then after I'd taken it off it would leave me a bit of a mark, and the Fitbit one was, well, it wasn't as bad. I would wear it if I have to, and if I had the cholce whether to wear it, I'd wear it now and then, but the only thing was thatI forgot it was there,and that was everything comfort, but I couldn't

remember to take it off, like to get in the bath. My Mum had to remind me, and it would be like in the morning it would be my Mum, "Take your Fitbit off for swimming, take your Fitbit off and put it underneath for canoeing", and that, and I didn't mind doing that, but it's just I'm awful at remembering, that's the only thing.

P1 Aah, well me too. I'm not very good at remembering.

P2 I can't even remember when my birthday is sometimes. I've got to think.

P1 And what was comfy about wearing the Fitbit and the GenieActive, if there was anything, that is.

P2 The GenieActive, there wasn't really anything comfortable with it, but with the Fitbit, it was light, and if there was a couple of days that I didn't have to take it off , and it was only for like the bath, my Mum could go, "Take your Fitbit of". It was fine. It didn't get in the way, and my class sort of reminded me a bit to take it off now and then. Like I had to take it off for PE a bit. It depended what we were doing, because now and then we would be doing judo and stuff where I might break it or land on it, and it might hurt me, and my class were better at remembering than I was. I sit next to this girl in my class, and she's my friend, and she was my reminder. She wasd like, "Remember to take it off", so that was helpful.

P1 Aah.So what device would you say was more comfy to wear out of the Fitbit and GenieActive?

P2 The Fitbit.

P1 And what made the Fitbit more comfy compared to the GenieActive?

P2 It was lighter, it was thinner, and a lot less noticeable, and it was just fun doing it, I think.

P1 Yes. Ah, that's good. You said that you agreed that the GenieActive got in the way of what you were doing, whereas the Fitbit did not get in the way of what you were doing, and you've touched on that a little bit. Can you tell me a bit more about that?

P2 The Fitbit, now and then I'd have to take it off, but thst doesn't really get in the way of what I was doing, and the GenieActive I could keep on at all times, but it was just clumpy and thick, and when I was wearing my PE kit, it's shorts, so it's noticeable, and it's just thick and gets in the way a lot.

P1 Can you give me an example of when it got in the way of what you were doing?

P2 In PE, I could keep it on, because it was the GenieActive one, and it was just I was scared in case someone whackered me there, and it digginhg in, and it was really

chuinky, so the sides of it might dig in, and when I take it off I'd have a little line where it's dug in a bit, and then when I put it on looser I didn't want it to fall off.

P1 Ah, yes. And what were you doing when you were a bit scared that it might dig into you?

P2 We were doing ju jitsu and acrobatics and stuff like that, because we do a lot of PE in our school. It's part of a health programme we do at school which is really good, and we do gymnastics, acrobatics where you can do a bit of cartwheeling and stuff like that, and that's stuff I like to do, because I learned how to do a cartwheel.

P1 Well done you! But what would you do to change the GenieActive and Fitbit if you could do anything to make it better?

P2 The GenieActive one I'd make it a lot thinner and a lot less like a man's watch, and make it for all ages, and for a boy or a girl, or a woman or a man, so it's more just you could pick a colour, so it would be nicer for you to wear.

P1 And how about the Fitbit? What wouldyou change, if you could do anything, to make that better, what would you change?

P2 I would make the Fitbit waterproof. That's the only thing I would change, make it waterproof.

P1 Ah, thanks, P2. Did you ever have to be told to remove the monitors at all?

P2 I got told once when my teacher said, "We're doing ju jitsu, so you might want to take it off", so we just wrote it down on the piece of paper. That was for the Fitbit, and then the GenieActive one, I got told to take it off when I was going to sleep, and the Fitbit my Mum would now and then tell me to take it off. If I was having a bath my Mum'd be like, "Oh, take it off", but I suppose that wasn't much effort for me, because my Mum was the one telling me to take it off. It's not like my teacher, so it's a lot.

I And what reasons did they give you for having to remove it, like so you mentioned there that your teacher in school told you to remove it while you were doing your ju jitsu, and also your Mum now and then told you.

P2 My Mum was telling me because it's not waterproof, so if I was going in water we don't want to break it, and then the GenieActive one, my teacher just didn't want someone to whack it or be messing round a bit, and knock it a bit or me fall to the ground and it dig in, or it falls off if someone stands on it.

P1 So it sounds like for safety reasons.

P2 And my teacher said for other pupils' safety as well, because if I put my hand out

and someone walks into my wrist, it's going to hurt, and it's just for safety reasons really.

P1 Ah, thanks, P2. Next I'd like to talk about other people like friends and family, and how they may have influenced you to wear the physical activity monitors, Did you ever forget to put the physical activity monitor on?

P2 No, not once. Now and then on the thing, it would have like twelve that's on like a Saturday, where I've woken up.

P1 Had a little lie-in?

P2 Yes, have like a long day, but that's not me forgetting to put it on. As soon as I woke up it would be on the kitchen with the sheet, and yes, it would just always be there, and then I'd come in, I'd take it off, put it there, go for my bath, come down, put it back on, come to going upstairs. I'll take it off as soon as I'm ready to go to sleep, give it to my Mum or to my Dad, and put it on the kitchen table.

P1 Ah, well, you've kind of answered the next question, which was obviously in the questionnaire you said that you strongly disagreed that you kept forgetting to put the physical activity monitor on, and how was it for you remembering to wear the monitor each day? How did you find that?

P2 Yes, I found it, every day it didn't take any time at all out of my day, so I was more than happy to put that into a routine, because there's no way to say strongly disagree, because I never once forgot to put it on, and we just had this little routine going on, and it didn't take much time up, so...

P1 Oh, brilliant. Because that was my next question, which was can you tell me how you remembered to wear the monitor each day, anf you told me you left it on the kitchen table, and you just popped it on in the morning. Was that the same for every day for each monitor?

P2 Every day, because, I don't know, every day it just seemed to be like that, or if I was waking up straight away, and my sister'd said, "Let's go swimming", I would before run down, get it off the table, put it on, and go and get dressed and do all of that, and then I'd go and I'd take it off then, because it'll still add steps to my day, so yes.

P1 Ah, thanks. P2. You said that you agreed that other people noticed that you were wearing the physical activity monitor. What did people say to you?

P2 On the Fitbit one, they only recognised it because I told them, but on the GenieActive one, people just generally recognised it because with my cardigan you could see a ginormous lump where it was, and you could see a line where I'd tried to tuck it in a bit and that, so I think it was just a lot more noticeable than the Fitbit one. Like if I would have not told anyone, I don't reckon anybody would notice

P1 No. what did people say to you?

P2 On the GenieActive one, it was more like, "Oh, what's that?" and "Is that a watch?" and stuff like that, and on the Fitbit one it was more like, "How many steps have you done?" and "Do you sleepwalk?" and funny questions like that.

P1 What did you think about what they said?

P2 On the GenieActive one I just said, "Oh, I'm just doing a test for the hospital" and that. I just kind of left it at that one. And then at the Fitbit one, I don't mind explaining it, because, I don't know, it was a lot easier to get people involved, and it was good. It was really good fun.

P1 And how did it make you feel, you know, people asking you about wearing the devices?

P2 On the GenieActive one, at the beginning it wasn't too bad, but then it got halfway through, and I'd told nearly everyone now, and I've had enough of telling people. And then on the Fitbit one, people don't notice what it is really, like a jelly band, and my class weren't constantly asking questions. It was like I'd go, "Oh, I'll tell you how many steps I've done" and so on, come in the next day and then tell them, and stuff like that. I didn't mind that at all.

P1 Oh, I'm glad about that. That's great.

P2 It was nice.

P1 Ah, that's good to hear. Did you notice a change in the amount of active activities you normally do as a family while you were wearing the physical activity monitors?

P2 No, I don't think anything changed, other than the school we were going to the canoeing, but that was the only thing that really changed, in like a daily basis. At the weekend it sort of was like a normal weekend. It was just nice. I went out with my sisters and my Mum and Dad, and we went shopping, and we got food, and it wasn't anything different really.

P1 Ah, so nothing different. Thanks, P2. How about your friends? Did how your friends are involved in you being active change?

P2 Not really, but they were making me run a bit more, like up and down the race track, and I think it was good fun, because why not be more active?

P1 So that made you run more?

P2 Not really. Say like we were doing nothing, we would tend to run or wander round, but if we had nothing to do it would be to make me run round the whole playground.

P1 And what did you think about that?

P2 It was funny, and I don't know, I didn't mind. I wasn't like, "Oh, I can't be bothered". It was just a bit like, "Oh, ok, then". It was fun. I liked it. I thought it was good.

P1 You thought it was good? Ah, that's good. I'm pleased. And what kind of made your friends think, "Let's make P2 run".

P2 Because they knew if I ran, my steps and my energy levels and that would change, and get higher and stuff like that. If I was to wear the Fitbit again, there wouldn't be much difference to it, to like not running, because we do loads of running. That's why.It was just like every day, and it wasn't just like, "Oh, I've increased my running". We were just all like playing games and that. It was just like a fun game to play. It wasn't like for basically the Fitbit. It was just a bit for fun.

P1 Oh, so would it be for just the Fitbit your friends asked you to run, or would it be for both devices?

P2 Just for the Fitbit,because the other device they could see that I wasn't very happy wearing it.

P1 Aah!

P2 I was a bit like that, and I was always like, "Oh, it's too big", and when the teachers ask why I've got it on I'd just be like, "It's my Dad's watch".

P1 Ah, thanks for that. So you've told me how your friends being involved in you being active changed a little bit, told you to run, and I asked you what you thought about that.You said you didn't mind. Is there anything that you did like about it particularly?

P2 The GenieActive one.

P1 Sorry, I didn't really explain that very well. So when your friends told you to run about and that, was there anything that you liked about that, or was there anything that you disliked about that?

P2 There was nothing that I disliked, and I just liked the fact that it was for a game, and it was just a big game for everyone, and it was just loads of fun, I think. There was nothing wrong with that. They weren't like telling me, they were like, "Oh, shall we go and run around and see how high we can get your Fitbit?" and like the dots, and every day I'd get to the highest one and that, and all my friends would be like this, looking over and I'd be like, "Yes, it's nearly there". So yes, I didn't mind at all.

P1 Ah, thanks for that, P2. on the feedback questionnaire you said that you agreed that other people like friends or family kept asking how much activity you do while you were wearing the physical activity monitors. What did you think about that?

P2 On the Fitbit, I liked it, as I told you. I liked it. And on the GenieActive one, I didn't mind family, but friends, they sort of didn't notice it a bit.

P1 And was that just because, as you mentioned, it was clunky, it was a little bit uncomfortable.

P2 Yes. It just looked quite like a man's watch, and I'm more like fashion and style, and football, and stuff like that.

P1 Yes. Thanks. Thank you for your answers. I'm learning a lot about what wearing physical activity monitors was like for you, and in this last section I'm going to explore how wearing a physical activity monitor may or may not have affected the activities that you do. Do you think wearing that wearing a physical activity monitor encouraged you to be more active?

P2 No. I reckon it was just the same, but people were asking questions, but other than that, I didn't think there was anything different.

P1 And how about other young people with CF? Do you think wearing the GenieActive and Fitbit would encourage them to be more active?

P2 It's depending what type of people you surround. Like there could be pushy people, and there could be people who just want you to sort of do your own thing with it, so it's really depending on the whole fact of who you're around.

P1 OK. And how do you think using the GenieActive and Fitbit could help you to be more active?

P2 I reckon if I did go back into hospital, and I lost a lot of weight, and I needed to get more active again, I reckon I'd get one, because it would help me to be more active, and my health would go up again, and I've tried to think what I've done over the last few days so I can tell you, and I reckon I've been thinking what was different, and I couldn't really think of anything.

P1 Oh yes.

P2 I've been trying to keep to what I've been doing with the Fitbit, because I think that, if the Fitbit's saying, and like scientists and that are saying, that's like healthy and good for you, then I should really keep carrying it on.

P1 Ah, thanks, P2. Can I just go back to that other question, when I asked you how other young people with CF do you think wearing the GenieActive and Fitbit would encourage them to be more active? You said something like it depends on them, whether they were a bit pushy or not, and I was just wondering if you could explain that a little bit more to me.

P2 Well, now and then people think, "I've got this. I want to try and be best in everyone else", and it's got to be dead good and high and that. When it does it, all you have to do really is do your normal days, and people go out of their way to change it, or just because they think that being good at this is going to change everything, when it's not. You've got to keep doing healthy activities and eating healthy food and stuff like that. It's not all to do with just the Fitbit.

P1 It sounds ljke you're saying that being healthy involves a lot of different things like eating healthy and keeping active, not just one thing.

P2 Yes. you've got to do a variety of different things, but you've got to remember the fact that you can't just keep to one variety of like fruits and vegetables. You've got to eat different, otherwise really you're just not going to get further than you are, and you're going to either get well below weight or above it, so it just helps a lot to eat different foods and try different things.

P1 That's a really good answer. Thank you. Can you tell me how you think using the GenieActive and Fitbit was useful or not useful at encouraging physical activity?

P2 It was. I knew that it was to compare a CF person's life, so I didn't do anything that would change my normal day, but as I said, if I did go into hospital, I think it would encourage me, because as I was saying about being pushy, I'm not pushy, but I'm a bit competitive, just a bit.

P1 And can you tell me a little bit more about that? So how did wearing the activity monitors would encourage you to be more active? You mentioned you're a little bit competitive

P2 Yes. Well, if there's like a score saying "Well, this is what you should be doing", saying like it's three thousand a day, I would get to three thousand a day, because, I don't know, I would just be like wandering round the room to get to that, because if I do three thousand a day, then that's going to make my health a lot better, and if I choose salad over a chocolate bar, have like the salad and then a bit of a chocolate bar, it's just going to help me a lot more, but it depends on who you're talking to, because different people need different diets, because now and then people listen to one diet, and think that's what I need when it's not. You've got to listen to a variety, like some people might be underweight, some people might be over, and some people might be allergic to most things, so they've got to try and find other stuff to fill in, and some people might be vegetarians, because meat's important as well, so you've got to try and fit all of them

things in to make it work, really.

P1 OK. and what was it about the GenieActive and Fitbit that you feel encouraged you to be more active?

P2 The fact that they were both, like if I was to have them again, and it was to see if I could get to a certain score, they would just make me, I don't know, because if you're not well, and you want to be well again maybe, all you need to do is do a bit more, get a bit more activity into your life, and I think that's encouraging, because if you're competitive like me, and you find a score on the Fitbit that you should be doing, if you try and get to that score, you will eventually if it takes like a week, two weeks, a month. You will get there eventually, so all you need to do is keep trying and stuff like that.

P1 You said there that with the Fitbit you could be a little bit competitive, you could see how much steps you could get to.It sounds like that encouraged you to be a bit more active. How about the GenieActive? How did you feel that would encourage you to be more active?

P2 I think it might encourage me a bit, but I like to see what I've done, but at the end of a day I didn't get to see, so it was just like normal anf that, but if I was to recommend either one, for this reason it would probably be the Fitbit.

P1 OK. And what was it about the GenieActive and /fitbit that you feel stopped you from being active?

P2 The Fitbit wasn't waterproof, and I've said this a milllion times, the GenieActive was big and like a man's watch,and thick.

P1 Thank you. Well, a little rest for you now, P2. It's a good time to ask you some questions, P3.

P3 Yes, fine.

P1 What do you think the benefits of wearing a physical activity monitor are for P2?

P3 Well, I think she was more aware. I think at the end of the day, especially with the, was it the Fitbit, when we eventually got roiund to logging on, and having a liottle look what the information did show you, it was interesting to see where she peaked and, you know how we realised that one day when you were out with your friend, how active you actually were during that short space of time?

P2 Yes, when we walked round rhe park, wasn't it?

P3 I think it was. I think it's a good reminder as well. I mean, I think most parents with CF children are aware of the benefits of exercise anyway, so you're aware of it,and it was

a little another thing to do every morning, like remembering to put it on and just filling it in, but it wasn't too arduous or anything. It was good, really, and I think certainly the Fitbit wes interestiong, because it gave you a lot more information. She was happy to wear it. It was pink for a girl.

P2 I like pink.

P1 And what do you think are the negatives of wearing a physical activity monitor are for P2?

P3 There weren't any, really. I mean, the first one was a bit too big for her wrist. The second one was a perfect fit. So there was no, I mean, they're always wearing watches, children, or bracelets or womething, so there was no negative really.

P1 Ah, thanks. And thinking about P2, do you feel that the activity monitors encouraged her to be more active?

P3 I don't think she was any more active. In fact, some days she was a bit less actrive, because I think you had a few days when you weren't so well, weren't you, I think.

P2 Yes.

P3 And I think the weather was bad a couple of days, so she wouldn't. I think it teemed for a few days during the...

P2 It was like three or something.

P3 I can't remember, so conbsidering the time of the year and the light nights, I think her activity was reduced on those days. But no, it did make you aware. I don't think she did any more than usual, but it made you aware of it. Do you know what I mean?

P1 Yes. Sounds like you're saying it gave her, I suppose a tangible thing to look at.

P3 Yes.

P1 Although you know that P2 is active, very active, you had something...

P3 It made me think, "Oh gosh, she's had a few quieter days.That's not typical ".Do you know what I mean? Or it made me thinking, "Oh, I bet you that one's really ...", like she's on rollerboots. And then interestingly though, I think when we knew it didn't show as a high level, because obviously she's just gliding everywhere. It's only really the bottom half of her body doing the work, so that was interesting as well, to think that maybe all the rollerblading isn't quite as benedicial as going for a swim or doing something else. No, you didn't do any more exercise, did you? But it made me aware of perhaps days that she did a bit less.

Phase 3 - P9

P1 And on those days when PO2 did do a bit less, if you were a little bit more aware of it, how would that make yoyu feel?

P3 Nothing. I was just thinking, "Oh, the results'll be interesting", I was thinking.maybe your results. You know, some days weren't particularly typical for her, but you never know with P", because she could be ill for a week,and it would be drastically reduced, or it could be a good night when she plays out every night, and she does this, this, this and this. So no two weeks are the same, really for P2 really.

P2 I was surprised, because I thought that the Fitbit would react a lot more to the rollerboots, and I thiuyght that the rolletboots would be more energies than running, so I was surprised at the fact that they were a lot, lot, lot less.

P1 A lot less. I wish I could give you a reason for that, to be honest, but Ican;t.

P3 I think it's just low impact. It must be low impact, mustn't it?

P2 Because you're gliding, but...

P1 Sorry. Do you think it's reasonable that for P2 wearing a physical activity monitor might influence the amounbt or type of activity that she;s done?

P3 I thnk it would, you know. Yes, I think it would.

P2 Like in what ways?

P3 Well, I think the fact that she's going up to the high school, and you can measure your steps, the Fitbit in particular, because we were able to visually see what she's been up to and things. I think the next part of her education, she's going to go to high school, she's going to be doing a lot more walking, a bigger campus, walking to get there, walking to her Nan's afterwards. So I do think it would benefit her wearing one as well. I think it would make her more aware. What was the question?

P2 Oh yes, it was just do you think wearing the physical activity monitor might influence the amount or type of activity that P2 does?

P3 Yes, I think it probably would.I think it would make me have a little nose and think about it more, and perhaps, I mean, I don;t know, it might be a novelty to begin with,mightn't it? And then I think you'd just balance out, wouldn't you? But I suppose, I think she's quite active anyway, on good days. I don't knpow whether it would change anything, because I think I already know she should be exercising. But I think it might for her be a good prompt.

P2 A good nudge.

P3 But it was good the way it flashed. I didn't quite understand the way it worked, I mean because we didn't take the time to ready everything, but I realised when it flashed, when you'd done the right amount of steps...

P2 It came up like five flashes.

P3 It was the flashing came up, and things like that. Anyway, somebody in work wears one, you see.

P1 I remember you saying. Ah, thanks for that. That's brilliant. Back to P2 now.

P3 Well, go on, P2.

P2 I'll just move here again, then. All you can hear is probably me clomping round.

P1 Oh, don't worry about that. That's fine. So the GenieActive shows that on average you did three hundred and sixty-three minutes, which is six hours and three minutes of moderate to vigorous activity each day. It's recommended that children and young people should do sixty minutes of moderate to vigorous activity each day to stay healthy. You did more than this. What do you think about that?

P2 I am actually amazed, because I'm dead happy because I really did think that I might have only just got there, but to know that I was past, I reckon it's a good aim to go for, and I reckon other children to try and aim for that as well.

P3 Well, I didn't realise that. So she did a little bit more than the average?

P1 She's done significantly more really, so..

P3 Which one was that? With them both?

P1 Well, the GenieActive is what we could get off recording of P2's movements.

P3 And some of those days you were putting it on a bit later, weren't you, at the weekend and things?

P1 It shows three hundred and sixty-three minutes, and on average they say that children and young people should do sixty minutes of moderate to vigorous activity each day. You've done significantly more.

P3 That's good news, isn't it?

P2 That explains my hyperactivity.

P3 A bit, yes.

P2 I believe you now, Mum.

P1 So it sounds like it msde you happy to hear that.

P2 Really, because I've got no words for it. It's just amazing. I can't believe I've actually got that many, and I reckon if you don't get the normnal amount, I reckon try and keep the Fitbit on for as long as you possible can, and try and aim for a higher score, even if it's by a few minutes or hours or whatever, you're still going to be doing more, and more is better than less.

P1 That's really good. Thanks, P2. The GenieActive shows that you did the most moderate to vigorous activity on Wednesday. What do you think helped you to be more active on this day?

P2 I know. We went canoeing on wednesday, I think.

P1 So while you were wearing the GenieActive, so the black one.

P2 Oh!

P3 Oh, the black one, on a Wednesday.

P1 Yes.

P3 The timetable for school wasn't usual, because it was around SATS time, so I think things were focusing around SATS. Wednesday you don't particularly have anything on Wednesday.

P2 It was probably me shaking.

P3 Swimming's on a Friday, for example.

P2 No, we're talking about the GenieActive one, the one that's waterproof.

P3 But you don't swim on a Wednesday. Maybe, your PE lesson's not on a Wednesday either.

P2 No. I think it was just shaking, the fact that SATS are so close. That's the only thing that I can really...

P1 OK, so the GenieActive reports like movement, so they say tri-axle, so it records movements forwards, sidewards, backwards I think, so it records like little bursts of activity, rather than still. Well, it does record stillness as well, but it wouldn't necessarily

record shakiness, like nerves and all that. So can you think of anything that might have been different around on a Wednesday?

P3 It couldn't have been your rollerskating.

P2 I know, but that wasn't on Wednesday. I know...

P3 We don't know what was on either.

P2 That was on the day that I went to the park with...

P3 No, you wouldn't go to the park on a Wednesday, P2, after school. Unless you were rollerblading, or maybe did youy go on the trampoline that night for a while?

P2 Trampoline.

P3 I don't know.

P2 When I got back it was nice, I think, on that day, and me and my niece was on the trampoline for like two hours.

P3 The trampoline. Or maybe it was your rollerblading, I don't know.

P2 The rollerblading didn't come up, so I reckon...

P3 That was on the pink one, though. P1's talking about the black one.

P2 I didn't do rollerblading on the black one. I reckon it's the trampoline.

P1 Trampolining. Okey dokey. Thanks for that.OK, so the GenieActive shows that on average you spend a hundred and forty-two minutes, which is two hours and twenty-two minutes, being sedentary, and this means doing things while you're still, like sitting and watching TV. what do you think about that?

P2 Quite shocking, actually.

P1 What's shocking about it?

P2 The fact that I'm sitting down for that length of time, and it sounds quite lazy.

P1 It sounds quite lazy, does it, to you?

P2 Does it record...

P1 How would it be for me if I said to you that that was actually quite a low number,

really?

P2 Was it?

P1 Yes.

P2 Oh.

P1 You'd think that was quite low. How would that be for you to hear that?

P2 Better, so I don't sound as lazy as I think I am now. Can I just ask a question?

P1 Course.

P2 You know, say if you're playing on an iPad. Does it record movement of the wrist like that?

P1 As far as I'm aware it doesn't. I don't really know a huge amount about the things. It more records five second spurts of activity.

P2 I don't know what I do when I sit down. The only thing I do is I'll go on my iPad or my phone or something. That's it, though.

P3 We watch a bit of telly of a night, don't we?

P2 Yes, unless it records me changing the channel on the TV. I can't think of a reason for having activity doing sitting down.

P3 In school you're not. You know, in a lesson you're sitting quite well. You're not...

P2 Oh, yes. During doing work, all I do is like swing my feet around or something, or I'd be like taking my shoes off, or I'll be writing. That's all we do.

P1 Does that sound, I was just wondering what you think about that, you know the amount of time that you spent sedentary? So a huindred and forty-two minutes, which is two hours twenty-two minutes. What do you think about that? Do you think that was good, or do you think that was bad?

P2 I don't know. I think everybody's got to have a rest during the day, but..

P1 I think you're right.

P2 Yes. But it doesn't feel like every time I sit down. It's like during the day, if I can put it all together, it doesn't feel like two hours. Like at school I could probably think of two hours, because if we're doing like a writing lesson, we're in our chair like forty-five

minutes, and then if we do a maths lesson that happens again. Yes, and it was around SATS, so yes, I think it was just each test was like an hour you had to do with them, so I can sort of work out now where it's all coming from, but I can't think of what I'll be doing activity-wise, if it doesn't record like hand movements that much.

P1 Thanks for that. The GenieActive shows that you were more sedentary on Friday and Wednesday. What do you think about that?

P2 I think they're the days that I've either gone on the trampoline, or Friday I've been swimming, and I think it's just them two days I've got a lot more time to myself to do activities, so yes.

P1 So it sounds like you've got more time on those days. My next question, it's a little bit contradictory, this, because I was going to say what was different about those days, the Friday and Wednesday, compared to Wednesday, when you were most active? So it's kind of like on the Wednesday you were most sedentary, but also on the Wednesday you were most active. What do you think about that?

P2 Confusing. I can't explain.

P3 The second Wednesday, wasn't that the day that the battery went, that you went on that day, and you had to take it off for the water sports?

P2 Yes. The second Wednesday was when my battery ran out, and...

P1 Ah, so I'm talking about the black one, so the clunky one.

P2 Oh.

P3 Is that when she did a lot or a little bit? A little bit?

P1 So I suppose my question was what was different about those days, Friday and Wednesday.

P3 On the black one?

P1 Yes, on the black one, compared to those days when P2 was most active, which was also a Wednesday.

P2 It was such a long time ago I can't remember what we did.

P1 Oh, that's ok if you don't remember the time. It was a long time ago, and to be fair, I couldn't remember what I did. I can't even remember yesterday, to be honest. I can't even remember this morning.

P2 I know. I can't even remember what I had for my breakfast this morning. I don't know. I can't answer that question fully. The only thing thing I could say was maybe I've gone on the trampoline or something, but because it was like three, two weeks ago, it's hard to answer the question, because I'm trying to think now, but it's just I've been ill, and then there's been days that I've been so active, and then not, so I really, honestly couldn't answer the question.

P1 That's fine, P2. Thanks. Did you change the amount of activity that you normally do while you were wearing the physical activity monitors?

P2 No, but if I was to get one again, for like a different reason like hospital said that I'm low on activities, I'd probably change it then, but doing it for like you and the other people that I join in the activity, no.

P1 OK. So you said that you might change it if you got one at the hospital, and it said that your activity level was low. How would you change your activity levels?

P2 I would try and go out a lot more. I'd start going on the trampoline a lot more, I'd start rollerblading, and I would start playing golf. Stuff that the rollerblading made a little bit, but a little bit can, if I do loads of it, a little bit can turn into more and more and more, and then along with the other activities, they'll help, like swimming and that, so yes, that's basically...

P1 Thanks. On the feedback questionnaire you said that you strongly disagreed that you tried new activities out while you wore the GenieActive, and that you didn't agree or disagree that you tried new activities out while you wore the Fitbit. Can you tell me a bit more about that?

P2 When I was out, like I went out with my friend one day, so I wouldn't have gone out with a friend normally, because that was just like there was nothing to do, but yes, so really that's it. Can you just repeat the first question?

P1 Yes, course. I was going to say, what the question says is, on the feedback questionnaire you said that you strongly disagreed that out new activities while you were on while you wore the GenieActive, and that you didn't agree or disagree that you tried new activities out while you wore the Fitbit, but you said you went out with your friends, and normally you wouldn't necessarily do that. Was that while you wore the Fitbit, or was that while you wore the GenieActive?

P2 It was while I wore the Fitbit, and I also wore the Fitbit as well for canoeing, which that was like a different week, because then two things happened, but it was a bit, one I wouldn't normally do the things that I did when I did them.

P1 And that was activities that you did in school, was it?

P2 No, that was just an activity.

P3 Like an extra-curricular thing.

P1 Oh, right. I see. Thanks, P2. Did you notice a change in how strenuous the activities you typically do are, like how fast you run or went on the trampoline?

P2 No, not really. Not at all, actually.

P1 No? Can you tell me about how you used the Fitbit? Like what settings on the Fitbit you used.

P2 I just kept it on like the day and the normal one. I thought if I wear it at night I'm such a wriggler. It's like a disaster waiting to happen, so I just thought it's easier to just keep it on the day, so if I keep it on the day, and yes.

P1 It sounds like you're saying you kept it on the day. And you've mentioned that you used the step counter, so when you tapped it. I was jsut wondering was there anything else that you used on the dashboard?

P2 Nothing that we didn't know how to use, but when I tapped it, I sort of tapped it quite a bit because I liked the feeling of when it made the buzz. It went, "Bzzzz".

P1 So you liked the buzz.

P2 Yes.

P1 What was it that you liked most about using the Fitbit and the functions that it's got?

P2 With the Fitbit it was just like the whole like you could see how many steps, and it buzzed, and it flashed, and stuff like that, and stuff that, I don't know. You think, "Well, if it does that, that means I've done loads of steps". I don't know, it'd more motivating than the GenieActive.

P1 More motivating than the GenieActive. And what did you dislike most about the Fitbit dashboard?

P2 You had to log in with loads of different like passwords and that, and I think it's a bit confusing for kids, getting them to do it on a computer. It'd be good if they made like an app for like iPads, where you could just constantly be logged on.

P1 Ah, I see. OK. I mean, I think they've got an app for a phone, like a mobile Smartphone, but obviously if you haven't got a mobile Smartphone, that means you haven't got that app, doesn't it?

P2 I know. We asked P, and P said it was iPhones, yes, but I don't always carry mine with me, because if I'm going swimming I'm not going to take it with me to the baths. It's only if I'm going out where I want to take pictures or I'm going out with my friends and my Mum needs to know if she needs to pick me up or where I am or if I get lost. So that's it.

P1 Thanks, P2. You mentioned on the feedback questionnaire that you agreed that getting some information each day would encourage you to do more activity. Can you tell me a bit more about that?

P2 Well, you could see what you're doing wrong during the day. Like if, I don't know, you're on the trampoline and you're only going on it for like say ten minutes, when it would be better if you're going on it for half an hour. You could change it and think, "Well, if I started doing this an odd time, when the trampoline's only got me so far, if I do twice as much it'll get me a lot further".

P1 And what is it about getting some information that tells you how active you are you feel would encourage you to be more active?

P2 You just want to be like the best you can be, and the best you can be is like checking, making sure that you're doing the right amount of steps, and if you're sitting down and you're bored, go and like wander around, or go on the trampoline or go and play outside and play football, something like that, so you're not just wasting your time sitting down. You're going and you're doing stuff, and that's going to help you get more steps in. That's the only thing I could really recommend to people to do.

P1 Thanks, P1. Just two more questions now. You said that you would like to wear a GenieActive and Fitbit again. Can you tell me a bit more about that?

P2 I think that it was fun doing the tests, and it's fun to think that people are actually going to be wearing when I need advice and stuff like that, so it's nice to think that you're helping a bit.

P1 So do you mean as part of the research?

P2 Yes. I liked being a part of the research, and seeing how they worked, and I thought they were really clever.

P1 And how about outside of research? Would you still like to get to wear them again?

P2 Yes. The only thing that I really enjoyed with the GenieActive one was being a part of like helping other people use them and stuff like that, and advice. And the Fitbit one I would wear because it was fun, and it was motivational, and you could see how many steps you've done, and it was just a lot easier to handle and cope with than the GenieActive.

Phase 3 - P9

P1 So outside of research, would you try and do the GenieActive and the Fitbit again, or would you just prefer to use one or the other?

P2 I would just prefer to maybe use the Fitbit.

P1 The Fitbit. Thanks. Last question. If you could ask the clinicians of the CF team at Alder Hey anything, what would you ask them?

P2 What's a clin...

P1 Ah, so you know the doctors and the nurses, and like P, and R, so all the people at Alder Hey in the CF team.

P2 The only thing I'd ask is if I added more into my day, would it be beneficial, or would it not make a difference? That would be the only thing I'd probably ask. And I'd probably ask if there was other choices like the phone app and like a little stick that went into your pocket. So I would probably ask which one would be best to use as a different thing. Like I've tried the GenieActive and the Fitbit, which were both like watches. Should I try like the little box or the phone app? Should I say if they would help more?

P1 And when you say help more, what does that mean to you, help more?

P2 Can you just repeat that question,please?

P1 Yes, sure. So you said that you'd maybe like to try the mobile phone app and the GenieAcitve, the box one, and you said just to see if they're better, that you'd like to ask them which is the best. I was just wondering what that would mean to you, something being the best.

P2 Like increasing my steps maybe, to make me do more. I don't know. Maybe there was a certain thing that counted other, like rollerblading and stuff more, just, I don't know, being more active.

P1 Sounds like you're trying to say maybe that what might be better than the other.

P2 Yes. Maybe there was one that, I tried two like a watch and a bracelet. Maybe if I had more pockets all the time, and maybe the little stick thing might be better for me, and maybe I'd enjoy having that more. Maybe it's more comfortable, maybe it's better for my lifestyle and what I do during the day.

P1 OK. Ah, thanks. That's a really good question. Thank you very much, P2. Thank you for speaking with me today. I've really enjoyed hearing what you thought about wearing the physical activity monitors. That's the end of the interview now. Is there anything more you'd like to add?

P2 No, I don't think there is.

P1 Maybe something I've missed?

P2 Don't forget to not charge the Fitbit, and if you do get a scare if you think you've been in the shower. That's the only thing I could physically add.

P1 Aah, thanks for that. And how about you, Mum? Have I missed anything, or anything that you'd like to add?

P3 No, no. I think it's been a good thing for P2 to be involved with really, because I think it's made her more aware as well, and I think a few of the results you've given then, to me I thought it would have been on the low side. I didn't realise she perhaps could be doing a little bit more than what was expected, because no two days are the same, so the school timetable changes a lot, and the weather.

P1 Yes.

P3 But I think it's been good for P2 to think about it. Because that'd be good for the kids for sure, for the children, and as I say, the Fitbit's like the latest gadget thing, isn't it? So that is going to be interesting, and if I thought I could get away with wearing one, if it'd have any impact on my life. Oh no, no, it's been interesting.

P1 Ah, that's brilliant. OK, I'll end the interview now. Well, thank you very much for your time.
